# Long-Time Dynamics for the Kelvin-Helmholtz Equations Close to Circular Vortex Sheets

**DOI:** 10.1007/s00205-026-02174-8

**Published:** 2026-04-27

**Authors:** Federico Murgante, Emeric Roulley, Stefano Scrobogna

**Affiliations:** 1 Universit Statale di Milano, Milan, Italy; 2 Scuola Internazionale Superiore di Studi Avanzati (SISSA), Trieste, Italy; 3 Universit degli Studi di Trieste, Dipartimento di Matematica, Informatica e Geoscienze, Trieste, Italy

## Abstract

We consider the Kelvin-Helmholtz system describing the evolution of a vortex-sheet near the circular stationary solution. Answering previous numerical conjectures in the 1990s physics literature, we prove an almost global existence result for small-amplitude solutions. We first establish the existence of a linear stability threshold for the Weber number, which represents the ratio between the square of the background velocity jump and the surface tension. Then, we prove that, for almost all values of the Weber number below this threshold, any small solution lives for almost all times, remaining close to the equilibrium. Our analysis reveals a remarkable stabilization phenomenon: the presence of both non-zero background velocity jump and capillarity effects enables us to prevent nonlinear instability phenomena, despite the inherently unstable nature of the classical Kelvin-Helmholtz problem. This long-time existence would not be achievable in a setting where capillarity alone provides linear stabilization, without the richer modulation induced by the velocity jump. Our proof exploits the Hamiltonian nature of the equations. More specifically, we employ Hamiltonian Birkhoff normal form techniques for quasi-linear systems together with a general approach for paralinearization of non-linear singular integral operators. This approach allows us to control resonances and quasi-resonances at arbitrary order, ensuring the desired long-time stability result.

## Presentation of the Problem and Main Result

The Kelvin-Helmholtz (KH) equations are a classic of fluid dynamics, modeling the intricate behavior of vortex sheets at the interface between fluids with different velocities. Since their introduction by Lord Kelvin and Hermann von Helmholtz in the nineteenth century [1–4], these equations have provided crucial insights into fundamental hydrodynamic phenomena, from the formation of ocean waves to atmospheric turbulence. The classic KH problem addresses the instability of a plane vortex sheet, where the jump in tangential velocity across an interface drives the system linearly, generating the well-known instability that defines Kelvin-Helmholtz phenomena. Of particular interest is the interplay between background velocity jump (${\texttt{b}}$) and capillarity effects ($\gamma$), which together determine critical stability thresholds and equilibrium states. This work explores the mathematical structures emerging from these interactions, with special focus on the Weber number $\beta \triangleq {\texttt{b}}^2/\gamma$, which naturally emerges in the equilibrium spectrum and governs the system’s stabilization properties.

We consider a planar Euler system for two irrotational fluids with the same density (constant equal to 1) separated by an interface $\Gamma (t)$ homeomorphic to a circle and parametrized by  This interface divides the plane into two open components $\Omega ^{\pm }(t)$ with $\Omega ^{-}(t)$ bounded and $\Omega ^{+}(t)$ unbounded. Given two functions $f^{\pm }:\Omega ^{\pm }\left( t \right) \rightarrow {\mathbb {R}}$, we define thatThe evolutionary system is thus composed of the following equations:1.1In the above set of equations, the quantities $u^{\pm },p^{\pm }$ are respectively the velocity field and pressure inside the domain $\Omega ^{\pm }.$ The parameter $\gamma \geqslant 0$ is the surface tension coefficient and  is the curvature defined byThe last equation in (1.1) implies that the vorticity distribution $\boldsymbol{\omega }$ is localized on the curve $\Gamma (t)$ at time *t*,  namely1.2In the case where1.3where *I* is a given interval of time, the system (1.1) was recast, in [5], as the *Contour Dynamic Equation (CDE)*1.4with1.5Throughout the document, we use the notation

$$\begin{aligned}\int _{{\mathbb {T}}}f(x)\text {d}x\triangleq \frac{1}{2\pi } \text {p.v.}\int _{-\pi }^{\pi }f(x)\text {d}x.\end{aligned}$$

We refer the reader to [5] for a derivation of (1.4) from (1.1). We also warn the reader about the change of notation for the surface tension and mean vorticity with respect to [5]. Also, here we write the system in a rotating frame with angular velocity $\Omega$, but one can easily follow the changes. An explicit computation shows that

1.6 $$\begin{aligned} \text {for any } {\texttt{b}}\in {\mathbb {R}} , \quad \left( \eta , \omega \right) = \left( 0, {\texttt{b}} \right) \quad \text {is a solution of }(1.4). \end{aligned}$$

Let us define the background velocity jump as

$$\begin{aligned} {\texttt{b}}\triangleq \int _{{\mathbb {T}}} \omega \left( x \right) \text {d}x, \end{aligned}$$

which is time-independent according to (1.4). We can define the invertible change of variables as

1.7 $$\begin{aligned} \psi _x \triangleq \omega - {\texttt{b}},  &   \psi = \partial _x^{-1} \left( \omega - {\texttt{b}} \right) . \end{aligned}$$

The change of variables (1.7) allows us to determine the evolution equation for $\psi$ modulo a real, time-dependent constant, which is1.8Since the system (1.4) depends only on $\left( \eta ,\psi _x \right)$, the projection onto the zeroth mode in (1.8), which includes the constant , does not influence its dynamics. Therefore, we disregard , and impose that $\psi$ belongs to the homogeneous Sobolev space ${\dot{H}}^s\left( {\mathbb {T}};{\mathbb {R}} \right) \triangleq \left. H^s\left( {\mathbb {T}};{\mathbb {R}} \right) / {\mathbb {R}} \right.$, where $H^s\left( {\mathbb {T}};{\mathbb {R}} \right)$ denotes the classical Sobolev space of periodic real-valued functions, see Section 3. We can now rewrite the system (1.4) in terms of the variables $\left( \eta , \psi \right)$ using (1.8) and get1.9 Let us recall that the *Birkhoff-Roth integral*, representing the velocity induced by the vortex sheet via the Biot-Savart law (we refer to [5] for more details on the subject), is defined as1.10 Notice that, using (1.10),1.11Therefore, the first equation of (1.9) preserves the average, and the natural phase space for $\eta$ is

$$\begin{aligned} H^s_0\left( {\mathbb {T}};{\mathbb {R}} \right) \triangleq \left\{ \eta \in H^s(\mathbb {T};\mathbb {R})\quad \text {s.t.}\quad \int _\mathbb {T}\eta (x)\, \textrm{d}x =0 \right\} .\end{aligned}$$

In the absence of capillarity $(\gamma =0)$, it is now understood that the problem is ill-posed in the Sobolev regularity [6–8], but admits weak solutions [9], while one has to require at least analytic regularity on the initial data in order to have a satisfactory local well-posedness theory [10–12]. It is also well-established [13, Chap. 9.3] that the presence of a background velocity jump $({\texttt{b}})$ across the interface drives linear instability in the system, generating the classical Kelvin-Helmholtz phenomenon. In contrast, surface tension $(\gamma )$ exerts a stabilizing effect at the linear level, allowing local-in-time solutions to the full nonlinear equations [14–18]. When stabilization is induced solely by capillarity, the existence time is limited to $T\sim \epsilon ^{-1}$, where $\epsilon$ represents the magnitude of the initial datum. Besides, in the physics literature [19] the authors perform several numerical simulations for (1.4) varying the Weber number *We* which corresponds, up to a period factor of $2\pi$, with the parameter

$$\begin{aligned}\beta \triangleq \frac{{\texttt{b}}^2}{\gamma },\end{aligned}$$

Which captures the interplay between the previous two mentioned opposite phenomena. In particular, despite the slightly different geometry considered, it is numerically conjectured in [19] that the behavior of the system below a critical Weber number, which is very close to (1.12), "*...is quite predictable by linear theory, even over long times...*", [19, p. 1939]. The aim of our work is to give a rigorous mathematical proof of this numerical conjecture. To do this, we exploit the Hamiltonian nature of the quasi-linear system (1.9). In contrast to the classical formulations using the Dirichlet-Neumann operator, here the system is quite explicit and related to singular integral operators; see (1.5). By introducing a novel framework that combines the Hamiltonian Birkhoff normal form procedure for quasi-linear Hamiltonian systems [20] with a generalization of the paralinearization method for singular integral operators developed in [21], we prove that the lifespan extends to $T\sim \epsilon ^{-\left( N+1 \right) }$ for any $N\in {\mathbb {N}}$. This result enables us to establish stability way beyond the classical local lifespan results of [14–18], leading to what we refer to as *almost global well-posedness*. The precise statement is given in the following theorem:

### Theorem 1.1

(Almost global existence of nearly circular vortex-sheets) Let

1.12 $$\begin{aligned} 0<\beta _1<\beta _2<4\left( 2+\sqrt{3} \right) . \end{aligned}$$

There exists a zero measure set $\mathcal {B}\subset \left[ \beta _1,\beta _2 \right]$ such that for any values $\gamma \in (0,\infty )$ of the surface tension and ${\texttt{b}}\in {\mathbb {R}}$ of the background velocity jump with

$$\begin{aligned}\frac{{\texttt{b}}^2}{\gamma }\in \left[ \beta _1,\beta _2 \right] \setminus \mathcal {B},\end{aligned}$$

for any $N\in {\mathbb {N}},$ there exists $s_0>0$ such that for any $s\geqslant s_0$ there exist $\varepsilon _0,c,C>0,$ such that for any $0<\varepsilon <\varepsilon _0$ and any initial datum

$$\begin{aligned} \left( \eta _0,\psi _0 \right) \in H_0^{s+\frac{1}{4}}\left( {\mathbb {T}}; \mathbb {R} \right) \times {\dot{H}}^{s-\frac{1}{4}}\left( {\mathbb {T}};\mathbb {R} \right) ,\qquad \text {with}\qquad \left\| \eta _0 \right\| _{H_0^{s+\frac{1}{4}}\left( {\mathbb {T}};\mathbb {R} \right) }+\left\| \psi _0 \right\| _{{\dot{H}}^{s-\frac{1}{4}}\left( {\mathbb {T}};\mathbb {R} \right) }\leqslant \varepsilon , \end{aligned}$$

the system (1.9) admits a unique classical solution

$$\begin{aligned}\left( \eta ,\psi \right) \in C^{0}\left( \left[ -T_{\varepsilon },T_{\varepsilon } \right] ; \ H_0^{s+\frac{1}{4}}\left( {\mathbb {T}};\mathbb {R} \right) \times {\dot{H}}^{s-\frac{1}{4}}\left( {\mathbb {T}};\mathbb {R} \right) \right) ,\qquad T_{\varepsilon }\geqslant c\varepsilon ^{-\left( N+1 \right) },\end{aligned}$$

with initial datum $\left( \eta _0,\psi _0 \right)$ and size

$$\begin{aligned}\sup _{t\in [-T_{\varepsilon },T_{\varepsilon }]}\left( \left\| \eta \left( t,\cdot \right) \right\| _{H_0^{s+\frac{1}{4}}\left( {\mathbb {T}};\mathbb {R} \right) }+\left\| \psi \left( t,\cdot \right) \right\| _{{\dot{H}}^{s-\frac{1}{4}}\left( {\mathbb {T}};\mathbb {R} \right) } \right) \leqslant C\varepsilon .\end{aligned}$$

### Remark 1.2

Let us make the following remarks about the previous theorem

1. *The almost-global well-posedness result of Theorem* 1.1*cannot* *be achieved in settings where capillarity serves only a stabilizing parameter*, i.e. when $\beta =0$. The Weber number $\beta$, which emerges naturally in the equilibrium spectrum $\left\{ \omega _{\gamma ,{\texttt{b}}}(j) \right\} _{j \in {\mathbb {Z}}^*}$ (see (1.13)), captures the interplay between capillarity stabilization and Kelvin-Helmholtz instability. Remarkably, the incorporation of the (physically relevant) interplay between background velocity jump and capillarity is the key factor that generates stability, in particular we can pass from a Sobolev ill-posed problem ($\gamma =0$) to a almost-global well-posed one when $\gamma$ is arbitrarily small and ${\texttt{b}}^2$ is comparable. The Weber number $\beta$ modulates the linear frequencies $\omega _{\gamma ,{\texttt{b}}}(j)$ in a non-trivial fashion and enables us to exclude resonances
  $$\begin{aligned} \omega _{\gamma ,{\texttt{b}}}(j_1)\pm \ldots \pm \omega _{\gamma ,{\texttt{b}}}(j_N)\not =0, \end{aligned}$$
  taking $\beta$ outside a suitable zero-measure resonant set $\mathcal {B}$. The non-resonance condition is a fundamental requirement for implementing the Hamiltonian Birkhoff normal form procedure in PDEs [20, 22–33].
2. Referring again to the work [19] the authors notice, at page 1939, that

We had hoped to see some repartition of energy from the $k=1$ mode to smaller scales over large times. However, for $We=10.0$ only a very slow increase is observed, if any, of the width of the active spatial spectrum.

This unexpected localization phenomenon is consistent with our analysis. Indeed we prove that, after a suitable change of variables, each Fourier mode $u_j$ can exchange energy only with $u_{-j}$ for very long times, as we shall explain later. This is a consequence of the non-resonance conditions and the Hamiltonian structure of the equation which gives the conservation of super-actions (see (1.20)).

3. We identify a threshold for the linear stability in (1.12). This constraint is imposed to ensure that the spectrum of the linearized equation at the trivial state $\left( \eta ,\psi \right) =\left( 0,0 \right)$ is purely imaginary (see Section 2.2), a necessary condition for implementing the Hamiltonian Birkhoff normal form argument. While this condition can be relaxed by restricting the phase space to $\textbf{m}$-fold solutions for sufficiently large $\textbf{m}$, such a restriction significantly narrows the class of admissible solutions. For more details, we refer the reader to Section 2.2 and [5]. Although one could adapt the following analysis by verifying the $\textbf{m}$-fold preserving properties of the transformations along the scheme, following a similar approach to [34].

4.. The parameter $\Omega$ in (1.4) represents the speed of rotation of the reference frame. Since the Kelvin-Helmholtz problem is invariant under rotations, $\Omega$ can be chosen arbitrarily without altering the shape of the solutions. We choose $\Omega$ as in (2.21). This choice simplifies some computations and does not affect generality.
5.. Global-in-time solutions with specific structures can be constructed, as demonstrated in [5], where we identified families of globally defined, uniformly rotating solutions. We also refer to [5, 35–43] for the construction of families of steady solutions in slightly different settings. However, it remains unclear whether the almost global existence solutions stated in Theorem 1.1 are actually global in time. The quasi-linear structure of the Kelvin-Helmholtz system, combined with the absence of dispersion due to the periodic boundary conditions, makes the global existence of the Cauchy problem currently out of reach.

**Ideas of the proof** While the Dirichlet-Neumann operator approach pioneered by Zakharov, Craig, and Sulem in [44, 45] has become standard for Water-Waves problems-and also employed to the two phase setting [18]- we employ an alternative formulation. Following previous works such as [46, 47], we utilize the Birkhoff-Roth integral operator formulation, which exploits the Dirac-$\delta$ structure of the vorticity (1.2) in conjunction with the Biot-Savart law to express the KH equations as a CDE. A crucial aspect of our approach is establishing that the KH equations thus derived possess a *Hamiltonian* structure (Section 2.1), which is the following

$$\begin{aligned} \begin{bmatrix} \eta _t \\ \psi _t \end{bmatrix} =\boldsymbol{J}\nabla H(\eta ,\psi ),\qquad \boldsymbol{J}=\begin{bmatrix} 0 &  -1\\ 1 &  0 \end{bmatrix}.\end{aligned}$$

We refer the reader to Remark 2.2 below for a comment on the unconventional notation that we use for the Poisson tensor. The Hamiltonian is related to the pseudo kinetic energy , the length  of the free boundary and the angular momentum  throughIn different geometrical contexts the Hamiltonian structure of the KH system was already presented by Benjamin-Bridges [48, 49]. Once this Hamiltonian formulation is established, we are methodologically committed to working with the specific equations that arise from it, as any deviation would compromise the Hamiltonian property, which is essential for our analysis. This Hamiltonian structure is a fundamental requirement for obtaining the almost global well-posedness result Theorem 1.1 using the Hamiltonian Birkhoff normal form of [20]. Since we are looking for a stability result near the trivial state $\left( \eta ,\psi \right) =(0,0),$ a quantity of interest is the linearization at this stationary solution. The linearized KH system there is written as

$$\begin{aligned} \begin{bmatrix} \eta _t \\ \psi _t \end{bmatrix} = {\boldsymbol{L}}_{\gamma , {\texttt{b}}} \left( D \right) \begin{bmatrix} \eta \\ \psi \end{bmatrix} , \qquad {\boldsymbol{L}}_{\gamma , {\texttt{b}}}\left( \xi \right) \triangleq \left[ \begin{array}{cc} 0 &  -\frac{\left| \xi \right| }{2} \\ { \gamma \left| \xi \right| ^2 - \frac{{\texttt{b}}^2}{2} \left| \xi \right| - \left( \gamma -{\texttt{b}}^2 \right) } &  0 \end{array} \right] .\end{aligned}$$

The associated spectrum is given by $\lambda _{\gamma ,{\texttt{b}}}^{\pm }(\xi )=\pm \textrm{i}\omega _{\gamma ,{\texttt{b}}}\left( \xi \right) ,$ with

1.13 $$\begin{aligned} \omega _{\gamma ,{\texttt{b}}}\left( \xi \right) =\sqrt{\frac{\gamma \left| \xi \right| }{2}}\sqrt{\left| \xi \right| ^2-\frac{\beta }{2}\left| \xi \right| +\beta -1},\qquad \beta \triangleq \frac{{\texttt{b}}^2}{\gamma }\cdot \end{aligned}$$

Here we see appearing the parameter $\beta$ that modulates the equilibrium frequencies. This parameter is homogeneous to a wave number (inverse of a length). The modulation is fundamental for avoiding resonances later in the Hamiltonian Birkhoff normal form. Let us mention that the modulation of the linear frequencies by an external or geometrical parameter has been used to avoid resonances and construct quasi-periodic solutions for fluid models, see [34, 50–58]. Observe that $\omega _{\gamma ,{\texttt{b}}}(\xi )$ is real for any $|\xi |\geqslant {1},$ provided that

$$\begin{aligned}0<\beta <4\left( 2+\sqrt{3} \right) .\end{aligned}$$

There emerges our linear stability threshold (see Eqs. (2.17) and (2.18)). This means that one gets linear stability for typically small oscillations at small scales where the stabilizing effects of the surface tension are dominant. Also notice that the asymptotic of the linear frequencies is superlinear, namely, as $\left| \xi \right| \rightarrow \infty$

$$\begin{aligned}\omega _{\gamma ,{\texttt{b}}}(\xi )\sim \sqrt{\frac{\gamma }{2}}\left| \xi \right| ^{\frac{3}{2}}.\end{aligned}$$

Our purpose is to prove a nonlinear stability result near the circular interface corresponding to $\left( \eta ,\psi \right) =(0,0).$ To do so, we are able to obtain a suitable energy estimate of the form, for any $N\in {\mathbb {N}},$

1.14 $$\begin{aligned}&\left\| \left( \eta ,\psi \right) \left( t \right) \right\| _s^2 \leqslant C\left( s \right) \left( \left\| \left( \eta ,\psi \right) \left( 0 \right) \right\| _s^2 + \int _0^t\left\| \left( \eta ,\psi \right) \left( \tau \right) \right\| _{s_0}^{N+1}\left\| \left( \eta ,\psi \right) \left( \tau \right) \right\| _s^2 \text {d}\tau \right) , \nonumber \\&\quad \forall 0< t < T, \end{aligned}$$

where we used the notation

$$\begin{aligned}\left\| \left( \eta ,\psi \right) \right\| _{s}\triangleq \left\| \eta \right\| _{H_0^{s+\frac{1}{4}}\left( {\mathbb {T}};\mathbb {R} \right) }+\left\| \psi \right\| _{{\dot{H}}^{s-\frac{1}{4}}\left( {\mathbb {T}};\mathbb {R} \right) }.\end{aligned}$$

Then, a bootstrap argument allows us to get an existence time of the form $T\geqslant c\varepsilon ^{-N-1}$ where $\varepsilon$ is the size of the initial datum. Notice that the above estimate is highly non-trivial for two reasons:

1. The right-hand side contains the same number of derivatives as the left-hand side, which is particularly delicate given the quasi-linear nature of the equations.
2. The integral term exhibits high homogeneity, a non-trivial property considering the quadratic nonlinearity of the equations.

To address the first obstacle, we perform a paralinearization of the Kelvin-Helmholtz system (1.9) together with a paradifferential reduction procedure to remove the space dependence in the positive order part, which allows to remain at the same level of regularity. The paralinearization result is given in Proposition 4.2 and writes as

1.15 $$\begin{aligned} \begin{aligned} \begin{bmatrix} \eta _t \\ \psi _t \end{bmatrix} =&{{{\,\mathrm {Op^{BW}}\,}}\left( {\boldsymbol{Q}}_{\gamma , {\texttt {b}}}\left( \eta ,\psi ; x, \xi \right) + {\boldsymbol{B}}_{\texttt {b}}\left( \eta ,\psi ; x \right) \left| \xi \right| - \text {i}V_{\texttt {b}}\left( \eta , \psi ;x \right) \text{ Id}_{\mathbb {R}^2}\ \xi \right) }\begin{bmatrix} \eta \\ \psi \end{bmatrix} \\  &+ {{{\,\mathrm {Op^{BW}}\,}}\left( {\boldsymbol{A}}_{\left[ 0 \right] } \left( \eta ,\psi ; x, \xi \right) \right) }\begin{bmatrix} \eta \\ \psi \end{bmatrix} + {\boldsymbol{R}}\left( \eta , \psi \right) \begin{bmatrix} \eta \\ \psi \end{bmatrix} , \end{aligned} \end{aligned}$$

where ${{{\,\mathrm{Op^{BW}}\,}}\left( \cdot \right) }$ denotes the Bony-Weyl quantization in (3.9). Each term of positive order has an explicit formula with1.16The matrix operator ${\boldsymbol{A}}_{[0]}$ is of order zero and ${\boldsymbol{R}}$ is a regularizing matrix operator up to a sufficiently large order. We believe that this paralinearization result is itself of interest for maybe future purposes. In our equations, the Dirichlet-Neumann operator does not explicitly appear, contrary to what occurs in the one-phase flat Water-Waves problem, see [44] and [59, Chap. 1]. Instead, the equation derived in (2.5) features nonlinearities of convolution type with nonlinear singular convolution kernels. The presence of these singular convolution kernels, rather than an explicit Dirichlet-Neumann operator, marks a fundamental structural difference. This requires a specialized paralinearization technique, which we construct in Section 4. Let us now expose our method to paralinearize the KH system. The key insight of our approach is that terms that resist standard paralinearization techniques (i.e. paraproducts, Bony paralinearization formula and composition of paradifferential operators) share a common structure of convolution type in the form

$$\begin{aligned} H \left( \eta \right) g \left( x \right) \triangleq \text {p.v.}\int _{-\pi }^{\pi } K \left( \eta ;x, z \right) \frac{g\left( x-z \right) }{z} \text {d}z , \end{aligned}$$

where the function $K \left( \eta ;x, z \right)$ exhibits regularity at $z=0$ comparable to $\eta$. By Taylor expanding the function $z\mapsto K \left( \eta ;x, z \right)$ at $z=0$ and applying paraproduct expansions, we derive that

1.17a $$\begin{aligned} H \left( \eta \right) g \left( x \right) =&\ \sum _{\textsf{j}=0}^\textsf{J} {{{\,\mathrm{Op^{BW}}\,}}\left( K_\textsf{j}\left( \eta ;x \right) \right) } \ \text {p.v.}\int _{-\pi }^{\pi } z^{\textsf{j}-1} g\left( x-z \right) \text {d}z \end{aligned}$$

1.17b $$\begin{aligned}&\ + \int _{-\pi }^{\pi } {{{\,\mathrm{Op^{BW}}\,}}\left( R\left( \eta ;x, z \right) \right) } g\left( x-z \right) \text {d}z + \text { l.o.t.}, \end{aligned}$$

with $J\in {\mathbb {N}}$, for any $j\in \{0,...,J\},$ $K_j$ being a *z*- independent function of *x* and *R* being a remainder satisfying $\left| R\left( \eta ;x, z \right) \right| =\mathcal {O}\left( z^{\textsf{J}+1} \right)$. This transformation reduces our analysis to two specific categories of terms, namely

- *Terms in the right-hand side of* (1.17a): Classical theory (see [60, p. 355]) establishes that for any $\textsf{j}\in \{0,...,\textsf{J}\},$
  $$\begin{aligned}\text {p.v.}\int _{-\pi }^{\pi } z^{\textsf{j}-1} g\left( x-z \right) \text {d}z = m_\textsf{j}\left( D \right) g,\end{aligned}$$
  where $m_\textsf{j}$ represents a Fourier multiplier of order $\textsf{j}$ which can be explicitly computed using [60, p. 355]. Thus, through composition theorems for paradifferential operators, we obtain
  $$\begin{aligned}&{{{\,\mathrm{Op^{BW}}\,}}\left( K_\textsf{j}\left( \eta ;x \right) \right) } \ \text {p.v.}\int _{-\pi }^{\pi } z^{\textsf{j}-1} g\left( x-z \right) \text {d}z\\&\qquad = {{{\,\mathrm{Op^{BW}}\,}}\left( K_\textsf{j}\left( \eta ;x \right) m_\textsf{j}\left( \xi \right) \right) } g + \text { bounded terms}. \end{aligned}$$
- *Terms in *(1.17b): We leverage the decay properties of *R* as $z\rightarrow 0$ to establish that these terms constitute paradifferential operators of order $-\left( \textsf{J}+1 \right)$ modulo smoothing operators, as detailed in Proposition 3.28.

The methodology outlined above is elaborated in detail in Section 4 and formalized in Proposition 4.2, representing one of the manuscript’s principal contributions. Our work demonstrates that effective paralinearization is possible even when using the Birkhoff-Roth formulation rather than the Dirichlet-Neumann operator approach. The recovered paradifferential structure in (1.15) exhibits similarities with pure-capillarity one-phase Water-Waves equations, which allows us to derive several established results concerning vortex sheets, including the necessity of capillarity for system stabilization (cf. [14]). Furthermore, we identify purely nonlinear, unstable terms characteristic of the KH equations (cf. the term $w_{\texttt{b}}$ in (1.16), which is non-nil even when ${\texttt{b}}=0$), highlighting the enhanced instability of KH compared to Water-Waves systems. For further analysis of these distinctive unstable terms, we direct the interested reader to Remark 4.3.

Once the paralinearization obtained, our goal is to remove the *x*-dependence of the positive order terms in order to run an energy estimate in the same Sobolev space, namely without loss of derivatives. This is done through a classical paradifferential reduction procedure requiring to reformulate the problem with complex variables. Defining the complex coordinates

$$\begin{aligned} U= \begin{bmatrix} u \\ \bar{u} \end{bmatrix} ,  &   u \triangleq \textsf{m}_{\gamma , {\texttt{b}}}(D)^{-1} \eta + \textrm{i}\textsf{m}_{\gamma , {\texttt{b}}}(D) \psi ,  &   \textsf{m}_{\gamma , {\texttt{b}}}\left( {\xi } \right) \triangleq \sqrt{\frac{{\left| \xi \right| }}{2\omega _{\gamma , {\texttt{b}}}\left( {\xi } \right) }}, \end{aligned}$$

the paralinearized KH system is equivalent to the complex Hamiltonian system

1.18 $$\begin{aligned} U_t&= {\boldsymbol{J}}_{\mathbb {C}} \ {{{\,\mathrm{Op^{BW}}\,}}\left( {\boldsymbol{A}}_{\frac{3}{2}}\left( U;x \right) \ \omega _{\gamma , {\texttt{b}}}\left( \xi \right) + {\boldsymbol{A}}_{1}\left( U;x, \xi \right) + {\boldsymbol{A}}_{\frac{1}{2}}\left( U;x \right) \ \left| \xi \right| ^{\frac{1}{2}} + {\boldsymbol{A}}_{\left[ 0 \right] } \left( U;x, \xi \right) \right) } U \nonumber \\&\quad +{\boldsymbol{R}}\left( U \right) U. \end{aligned}$$

In the above system, each ${\boldsymbol{A}}_{m}$ corresponds a matrix of *x*-dependent symbols of order *m*, while $\boldsymbol{R}$ is a matrix of regularizing operators up to any fixed order. Then we follow the Hamiltonian method developed by the first author and collaborators [20] (see also [21, 61–65] for non Hamiltonian approach) which consists to perform a series of transformations:

- **i** we first perform an *Alinhac Good Unknown* transformation — a nilpotent matrix-valued paradifferential change of variable introduced in [66, 67]. This transformation eliminates the unbounded terms ${\boldsymbol{B}}_{\texttt{b}}$ in (1.16), which constitute the only unbounded contributions in the one-phase gravity water wave system, thus proving essential for developing local well-posedness theory in the pure gravity setting;
- **ii** we then *diagonalize* and *reduce to constant coefficients* the resulting system at arbitrary order, modulo smoothing operators (whose regularizing effects depend on the initial data’s regularity). This technique, initially developed in [61], has become standard for implementing normal-form techniques in quasi-linear systems [20, 65, 68]. The method involves conjugation with flows generated by paradifferential operators, where generators are selected based on desired cancellations. We reduce the equation to a diagonal, paradifferential constant-coefficient form by iterative application on the degrees of the paradifferential operators.

At the end of such a procedure we are able to define a transformed, equivalent (in Sobolev) variable

$$\begin{aligned} W \triangleq \boldsymbol{B}(U)U,  &   W = \begin{bmatrix} w \\ \bar{w} \end{bmatrix} \, \end{aligned}$$

that satisfies a *constant-coefficient*, *scalar* equation given by1.19up to a smoothing remainder $\pmb {\textsf{R}}$ (see Proposition 6.1 and (3.11) for the definition of ${{\,\mathrm{Op^{BW}_{vec}}\,}}\left( \cdot \right)$). The transformed equation (1.19) has the crucial property that its para-differential part is in constant-coefficient form. To overcome the second obstacle Item 2., we aim to implement a Hamiltonian Birkhoff normal form up to homogeneity degree *N*. However, unlike the original complex system (1.18), (1.19) no longer possesses the fundamental Hamiltonian structure. This structure is essential to ensure that certain *non-trivial* resonant terms do not contribute to energy estimates, as explained below. Recovering this structure is the purpose of the Darboux symplectic corrector as designed in [20]. To understand why this correction is needed, we first examine the role of non-resonance conditions. The non-resonance conditions in Section 2.3 ensure the exclusion of resonances, meaning that

$$\begin{aligned} \sigma _1\omega _{\gamma ,{\texttt{b}}}(j_1) + \dots + \sigma _N\omega _{\gamma ,{\texttt{b}}}(j_N) \ne 0, \end{aligned}$$

unless the indices $(\sigma _1, \dots , \sigma _N) \in \{\pm \}^N$ and $(j_1, \dots , j_N) \in {\mathbb {Z}}^N$ are *super-action preserving* (see Definition 2.6). This can happen when $N = 2p$ is even, with

$$\begin{aligned} \sigma _1 = \dots = \sigma _p = +, \qquad \sigma _{p+1} = \dots = \sigma _{2p} = -, \end{aligned}$$

and either

$$\begin{aligned} \text {(i)}\ j_\ell = j_{p+\ell } \qquad \text {or } \qquad \text {(ii)} \ j_\ell = -j_{p+\ell }. \end{aligned}$$

Case (i) corresponds to *trivial resonances*. The associated monomials in the vector fields of (1.19) take the form

$$\begin{aligned} |u_{j_1}|^2 \dots |u_{j_{p-1}}|^2 u_{j_p} e^{\textrm{i}j_p x}. \end{aligned}$$

Proving that these terms do not contribute to Sobolev energy estimates is typically straightforward, as it suffices to show that their coefficients are purely imaginary. In contrast, case (ii) involves monomials of the form

$$\begin{aligned} u_{j_1} \overline{u_{-j_1}} \dots u_{j_{p-1}} \overline{u_{-j_{p-1}}} u_{j_p} e^{-\textrm{i}j_p x}. \end{aligned}$$

These terms couple different Fourier modes, making it more challenging to show that they do not affect energy estimates. However, if the vector field possesses the strong algebraic property of being Hamiltonian, these monomials — called *super-action preserving* — automatically admit infinitely many conservation laws, known as *super-actions*. Specifically, for any $n \in {\mathbb {N}}$, the quantities

1.20 $$\begin{aligned} J_n(u) \triangleq |u_n|^2 + |u_{-n}|^2 \end{aligned}$$

are conserved. As a consequence, a Hamiltonian, super-action preserving vector field remains transparent to any Sobolev energy estimate. However, the system (1.19) for *W* lacks this Hamiltonian structure, preventing direct application of these conservation laws. To overcome this issue, we introduce the Darboux symplectic correction, as detailed in Proposition 7.1, restoring the necessary structure and allowing us to exploit these properties effectively. Since the map ${\boldsymbol{B}}(U)$ satisfies the hypotheses of [20, Theorem 7.1], we apply it to obtain a new constant-coefficient equation:1.21This equation is Hamiltonian up to homogeneity *N*, meaning thatfor some real Hamiltonian function $H_{\leqslant N}$. At this point, we begin the algorithmic procedure of reducing the degrees of homogeneity (see Proposition 7.9). The final outcome is a super-action preserving Hamiltonian equation of the form1.22Since the super-action preserving Hamiltonian terms ${\textrm{i}\boldsymbol{\omega }_{\gamma ,{\texttt{b}}}(D)} Z$, $\boldsymbol{J}_\mathbb {C}\nabla H^{(\texttt{SAP})}_{\frac{3}{2}}(Z)$, and $\boldsymbol{J}_\mathbb {C}\nabla H^{(\texttt{SAP})}_{-\varrho }(Z)$ do not contribute to the energy estimate, we obtain, for small solutions $\Vert Z\Vert \sim \Vert U\Vert _s \lesssim \varepsilon$, the energy bound

$$\begin{aligned} \frac{\textrm{d}}{\textrm{d}t} \Vert Z\Vert _s^2 \lesssim \varepsilon ^{N+3}, \end{aligned}$$

which allows us to prove Theorem 1.1.

**Structure of the manuscript** The Hamiltonian formulation can be found in Section 2.1 while the non-resonance conditions are proved in Section 2.3. The paralinearization of the system (1.9) is carried out in Section 4 and the final result is stated in Proposition 4.2. The next step is to reformulate the results using the complex notation, see Section 5. Then, we implement a reducibility procedure to get rid of the space dependence of the positive order part. This part is now rather classical and the corresponding final result is given in Proposition 6.1. With this in hand, one can perform the Hamiltonian Birkhoff normal form. It is done in Section 7.2 and requires non-resonance conditions for frequency vectors composed with the equilibrium spectrum. Such conditions are checked in Section 2.3 and are the reasons for the introduction of the zero measure set $\mathcal {B}$.

## Hamiltonian Structure and Non-resonance Conditions

Here we highlight the Hamiltonian nature of the system (1.9). Then, we study the associated linearization at the trivial solution $\left( \eta ,\psi \right) =\left( 0,0 \right)$ and discuss the non-resonance property of the corresponding eigenvalues. This latter fact is crucial for implementing the Birkhoff normal form in Section 7.

### Derivation of the Hamiltonian Formulation

Let us now exhibit the Hamiltonian nature of the Kelvin-Helmholtz system (1.9).

#### Proposition 2.1

The system (1.9) is Hamiltonian. More precisely, let us consider that2.1where   and  are the pseudo

kinetic energy, the length of the free boundary and the angular momentum, respectively, defined by2.22.32.4Then, the equations (1.9) are equivalent to

2.5 $$\begin{aligned} \begin{bmatrix} \eta _t \\ \psi _t \end{bmatrix} = \begin{bmatrix} -\nabla _\psi H(\eta ,\psi ) \\ \nabla _\eta H(\eta ,\psi ) \end{bmatrix} = {\boldsymbol{J}} \ \nabla H \left( \eta , \psi \right) , \qquad {\boldsymbol{J}}\triangleq \begin{bmatrix} 0& -1\\ 1& 0 \end{bmatrix}. \end{aligned}$$

In addition, the Hamiltonian *H* is reversible and invariant under translations, namely defining the transformations

$$\begin{aligned}\texttt{S}\begin{bmatrix} \eta \\ \psi \end{bmatrix}(x)=\begin{bmatrix} \eta \\ -\psi \end{bmatrix}(-x),\qquad \textsf{t}_\varsigma \begin{bmatrix} \eta \\ \psi \end{bmatrix}(x)=\begin{bmatrix} \eta \\ \psi \end{bmatrix}(x+\varsigma ),\end{aligned}$$

we have

2.6 $$\begin{aligned} H\circ \texttt{S}=H=H\circ \textsf{t}_\varsigma ,\qquad \forall \varsigma \in {\mathbb {T}}. \end{aligned}$$

#### Remark 2.2

We note that the choice of the Poisson tensor $\boldsymbol{J}$ in Eq. (2.5) differs by a sign from the standard convention (see, for instance, [20]). We adopt this convention, which ultimately reflects a choice of sign in the definition of $\omega$ in (1.2), so that the Hamiltonian is positive definite.

#### Proof

$\blacktriangleright$ *The pseudo kinetic part:*

We compute the variation of  with respect to $\psi$. Using integration by parts and (1.11), we get2.7From (2.7), we deduce that2.8Now we turn to the differentiation of  with respect to $\eta$. Recall the notation in (1.3). Differentiating (2.2) with respect to $\eta$, we infer thatBy symmetry, we can reduce this expression towhich implies in turn,2.9$\blacktriangleright$ *The length part:*

Recall that  with $h=\sqrt{1+2\eta }-1.$ Therefore,Thus,Differentiating, we obtainIntegrating by parts, we getIt is easy to see thatAs a consequence,Applying the chain rule, we deduce that2.10$\blacktriangleright$ *The momentum part:*

One readily has thatand, via integration by partsHence,2.11Gathering (2.1), (2.8) and (2.11), we get2.12Putting together (2.1), (2.9), (2.10) and (2.11) yields2.13Comparing (1.9) with (2.12) and (2.13) concludes the desired result.

$\blacktriangleright$ *Invariances :* The properties (2.6) are easily obtained by changes of variables $x\mapsto -x$ and $x\mapsto x+\varsigma$. This ends the proof of Proposition 2.1. $\square$

### Analysis of the Linearization of (1.9)

#### Definition 2.3

Let $m\in \mathbb {R}$, we define the space of Fourier multipliers of order *m*, $\tilde{\Gamma }^m_0$, as the space of smooth functions from $\mathbb {R}\setminus \{0\}$ to $\mathbb {C}$ of the form $\xi \mapsto a\left( \xi \right)$ such that

$$\begin{aligned} \left| \partial _\xi ^\alpha a \left( \xi \right) \right| \leqslant C_\beta \left\langle \xi \right\rangle ^{m-\alpha }, \  &   \forall \alpha \in \mathbb {N}, \left| \xi \right| \geqslant 1/2. \end{aligned}$$

Following [5], the linearization of (1.9) around $\left( \eta , \psi \right) =\left( 0, 0 \right)$ is given by

2.14 $$\begin{aligned} \left\{ \begin{aligned}&\eta _t = \left( \Omega - \frac{{\texttt{b}}}{2} \right) \eta _x - \frac{\left| D \right| }{2} \psi ,\\&\psi _t = \left( \gamma \left| D \right| ^2 - \frac{{\texttt{b}}^2}{2} \left| D \right| - \left( \gamma -{\texttt{b}}^2 \right) \right) \ \eta +\left( \Omega -\frac{{\texttt{b}}}{2} \right) \psi _x. \end{aligned} \right. \end{aligned}$$

Namely, we can write (2.14) as

2.15 $$\begin{aligned} \begin{bmatrix} \eta _t \\ \psi _t \end{bmatrix} = {\boldsymbol{L}}_{\gamma , {\texttt{b}}} \left( D \right) \begin{bmatrix} \eta \\ \psi \end{bmatrix} ,  &   {\boldsymbol{L}}_{\gamma , {\texttt{b}}}\left( \xi \right) \triangleq \left[ \begin{array}{cc} \textrm{i}\left( \Omega - \frac{{\texttt{b}}}{2} \right) \xi &  -\frac{\left| \xi \right| }{2} \\ { \gamma \left| \xi \right| ^2 - \frac{{\texttt{b}}^2}{2} \left| \xi \right| - \left( \gamma -{\texttt{b}}^2 \right) } &  \textrm{i}\left( \Omega - \frac{{\texttt{b}}}{2} \right) \xi \end{array} \right] . \end{aligned}$$

The eigenvalues of ${\boldsymbol{L}}_{\gamma , {\texttt{b}}}\left( \xi \right)$ are given by

2.16 $$\begin{aligned} \lambda ^\pm _{\gamma , {\texttt{b}}}\left( \xi \right) \triangleq \textrm{i}\left( \Omega - \frac{{\texttt{b}}}{2} \right) \xi \pm \, \sqrt{-\frac{\left| \xi \right| }{2} \left( \gamma \left| \xi \right| ^2 - \frac{{\texttt{b}}^2}{2} \left| \xi \right| - \left( \gamma -{\texttt{b}}^2 \right) \right) } \ \in \tilde{\Gamma }^{3/2}_0 \ . \end{aligned}$$

We want the eigenvalues in (2.16) to be purely imaginary, this happens if and only if

2.17 $$\begin{aligned} \gamma \left| \xi \right| ^2 - \frac{{\texttt{b}}^2}{2} \left| \xi \right| - \left( \gamma -{\texttt{b}}^2 \right) > 0, \end{aligned}$$

The condition (2.17) is satisfied for any $|\xi |\geqslant 1$ if

$$\begin{aligned} \beta \triangleq \frac{{\texttt{b}}^2}{\gamma } \in [0,\beta _{+}),\qquad \beta _{+}\triangleq 4(2+\sqrt{3}) \approx 14,928... \end{aligned}$$

Indeed, for $|\xi |\in [ 1,2]$, one has

$$\begin{aligned} \gamma \left| \xi \right| ^2 - \frac{{\texttt{b}}^2}{2} \left| \xi \right| - \left( \gamma -{\texttt{b}}^2 \right) \geqslant \min \left\{ \frac{{\texttt{b}}^2}{2}, 2\gamma \right\} >0 \end{aligned}$$

for any $\beta >0$. Meanwhile, for $|\xi |>2$, This reduces to

2.18 $$\begin{aligned} \beta < \min _{|\xi |>2 } \left( \frac{ |\xi |^2-1}{\frac{|\xi |}{2}-1}\right) =4(2+\sqrt{3}). \end{aligned}$$

#### Remark 2.4

We consider the restriction in (2.17) for $|\xi |\geqslant 1$. If we further restrict to $\xi \in \mathbb {Z}^*$, we can improve $\beta _+$ from $4(2+\sqrt{3})$ to $\beta _+ = 15$. However, in Section 2.3, we extend the function $\lambda ^\pm _{\gamma , {\texttt{b}}}(\xi )$ to apply the Delort-Szeftel Theorem 2.8. We believe that the argument in Proposition 2.9 could be modified to maintain the slightly less restrictive condition $\beta _+ = 15$. Nevertheless, to preserve the simplicity of our approach, we do not pursue this further analysis.

Then we obtain that

2.19 $$\begin{aligned} \lambda ^\pm _{\gamma , {\texttt {b}}}\left( \xi \right)= &   \text {i}\left( \Omega - \frac{{\texttt {b}}}{2} \right) \xi \pm \text {i}\ \omega _{\gamma , {\texttt {b}}}\left( {\xi } \right) , \nonumber \\ \omega _{\gamma , {\texttt {b}}}\left( {\xi } \right)\triangleq &   \sqrt{\frac{\left| \xi \right| }{2} \left( \gamma \left( \left| \xi \right| ^2 -1 \right) - {\texttt {b}}^2 \left( \frac{\left| \xi \right| }{2} -1 \right) \right) } \in \tilde{\Gamma }^{3/2}_0 \ . \end{aligned}$$

Notice that we can expand $\omega _{\gamma , {\texttt{b}}}\left( {\xi } \right)$ and obtain that

2.20 $$\begin{aligned} \omega _{\gamma , {\texttt{b}}}\left( {\xi } \right) = \sqrt{\frac{\gamma }{2}} \ \left| \xi \right| ^{\frac{3}{2}} - \frac{1}{\sqrt{2\gamma }} \left( \frac{{\texttt{b}}}{2} \right) ^2 \ \left| \xi \right| ^{\frac{1}{2}} +\omega _{\gamma , {\texttt{b}};-\frac{1}{2}}\left( {\xi } \right) ,  &   \omega _{\gamma , {\texttt{b}};-\frac{1}{2}}\left( {\xi } \right) \in \tilde{\Gamma }^{-\frac{1}{2}}_0. \end{aligned}$$

In the sequel, we make the following natural choice for $\Omega$ which does not alter the structure of the equation, as it is explained in Remark 1.2, Item 4:

2.21 $$\begin{aligned} \Omega \triangleq \frac{{\texttt{b}}}{2}\cdot \end{aligned}$$

#### Remark 2.5

Already at linear level standard computations show that in order to close energy estimates for the system (2.14) we need a discrepancy in regularity between $\eta$ and $\psi$, namely we can close the energy estimates on (2.14) in the case in which $\eta \in H^{s + \frac{1}{4}}_0\left( {\mathbb {T}};{\mathbb {R}} \right)$ and $\psi \in {\dot{H}}^{s-\frac{1}{4}}\left( {\mathbb {T}};{\mathbb {R}} \right)$, such regularity gap shall persist at nonlinear level as well. This is not unexpected, and the same behavior is present for the one-phase water waves problem, both at linear and nonlinear level, cf. [69].

### Non-resonance Conditions

In this subsection, we study the non-resonances between the frequencies. This is needed in the application of the normal form algorithm performed in Section 7.2. Specifically, we prove that there are no resonances between linear frequencies, except for the *super-action-preserving* ones, as defined below.

#### Definition 2.6

*(*$\texttt{SAP}$ *multi-index)* A multi-index $(\alpha , \beta ) \in \mathbb {N}^{\mathbb {Z}^*} \times \mathbb {N}^{\mathbb {Z}^*}$ is *super-action preserving * if

2.22 $$\begin{aligned} \alpha _n + \alpha _{-n} = \beta _n + \beta _{-n} \ , \qquad \forall n \in \mathbb {N}\, . \end{aligned}$$

A super-action preserving multi-index $(\alpha ,\beta )$ satisfies $|\alpha | = |\beta |$ where $|\alpha | \triangleq \sum _{j \in \mathbb {Z}^*} \alpha _j$. If a multi-index $(\alpha , \beta ) \in \mathbb {N}^{\mathbb {Z}^*} \times \mathbb {N}^{\mathbb {Z}^*}$ is not super-action preserving, then the set

2.23 $$\begin{aligned} \mathfrak N(\alpha , \beta ) \triangleq \Big \{ n \in \mathbb {N}\quad \text {s.t.}\quad \alpha _{n} + \alpha _{-n} - \beta _n -\beta _{-n} \ne 0 \Big \} \end{aligned}$$

is not empty and, since

$$\begin{aligned}\mathfrak N(\alpha , \beta ) \subset \Big \{ n \in \mathbb {N}\quad \text {s.t.}\quad \alpha _{n} + \alpha _{-n} +\beta _n +\beta _{-n} \ne 0 \Big \},\end{aligned}$$

its cardinality satisfies

2.24 $$\begin{aligned} | \mathfrak N(\alpha , \beta ) | \leqslant |\alpha + \beta | = |\alpha | + | \beta | \, . \end{aligned}$$

The main result of the present section is the following:

#### Proposition 2.7

Let $M\in {\mathbb {N}}^*$ and $0<\beta _1<\beta _2<4\left( 2+\sqrt{3} \right) .$ Then, there exist $\tau ,\delta >0$ and a zero measure set $\mathcal {B}\subset \left[ \beta _1,\beta _2 \right]$ such that for any $\beta \in \left[ \beta _1,\beta _2 \right] \setminus \mathcal {B}$ the following holds: there is $\nu >0$ such that for any multi-index $\left( \alpha ,\alpha ' \right) \in ({\mathbb {N}}^*)^{{\mathbb {Z}}^*}\times ({\mathbb {N}}^*)^{{\mathbb {Z}}^*}$ of length $\left| \alpha +\alpha ' \right| \leqslant M,$ which is not super-action preserving (in the sense of Definition 2.6), one has that

$$\begin{aligned}\left| \vec {\omega }_{\gamma ,{\texttt{b}}}\cdot \left( \alpha -\alpha ' \right) \right| \geqslant \frac{\nu }{\left( \displaystyle \max _{j\in \text {supp}\left( \alpha \cup \alpha ' \right) }|j| \right) ^{\tau }},\end{aligned}$$

where

$$\begin{aligned}\vec {\omega }_{\gamma ,{\texttt{b}}}\triangleq \left( \omega _{\gamma ,{\texttt{b}}}\left( {j} \right) \right) _{j\in {\mathbb {Z}}^*}.\end{aligned}$$

The rest of Section 2.3 is dedicated to the proof of Proposition 2.7. This latter is a consequence of the Delort-Szeftel Theorem that we recall here for the convenience of the reader; for its proof, we refer to [70, Theorem 5.1].

#### Theorem 2.8

Let $d\in {\mathbb {N}}^*,$ $r_0>0$ and $\beta _1,\beta _2\in {\mathbb {R}}.$ We denote $B\left( 0,r_0 \right) \subset {\mathbb {R}}^d$ the ball centered at the origin and of radius $r_0.$ Consider $f:B\left( 0,r_0 \right) \times \left[ \beta _1,\beta _2 \right] \rightarrow {\mathbb {R}}$ a continuous sub-analytic function and $\rho :B\left( 0,r_0 \right) \rightarrow {\mathbb {R}}$ a non-zero real-analytic function. We assume the following facts:

1. The function *f* is real-analytic on $\left\{ x\in B\left( 0,r_0 \right) \quad \text {s.t.}\quad \rho (x)\ne 0 \right\} \times \left[ \beta _1,\beta _2 \right] .$
2. For any $\bar{x}\in B(0,r_0)$ with $\rho (\bar{x})\ne 0,$ the equation $f(\bar{x},\beta )=0$ admit finitely many solutions in $\left[ \beta _1,\beta _2 \right] .$

Then, there exist $N_0\in {\mathbb {N}}$ and $\alpha _0,\delta ,C>0$ such that for any $\alpha \in (0,\alpha _0]$, any integer $N\geqslant N_0$ and any $x\in B(0,r_0)$ with $\rho (x)\ne 0$, we have

$$\begin{aligned}\left| \left\{ \beta \in \left[ \beta _1,\beta _2 \right] \quad \text {s.t.}\quad \left| f(x,y) \right| \leqslant \alpha \left| \rho (x) \right| ^{N}\right\} \right| \leqslant C\alpha ^{\delta }\left| \rho (x) \right| ^{N\delta }.\end{aligned}$$

First observe that we can write that

2.25 $$\begin{aligned} \omega _{\gamma , {\texttt{b}}}\left( \xi \right) =\sqrt{\frac{\gamma \left| \xi \right| }{2}}\sqrt{\left| \xi \right| ^2-\frac{\beta }{2}\left| \xi \right| +\beta -1},\qquad \beta \triangleq \frac{{\texttt{b}}^2}{\gamma }\cdot \end{aligned}$$

Notice that $\omega _{\gamma ,{\texttt{b}}}(2)=\sqrt{3\gamma }$ is independent of $\beta$ and that

$$\begin{aligned} \omega _{\gamma ,{\texttt{b}}}(5)- \sqrt{5} \omega _{\gamma ,{\texttt{b}}}(3)\equiv 0. \end{aligned}$$

The above relation shows that there is no resonance between the modes 3 and 5. However, the couple (3, 5) appears to be singular in the analysis of the non-degeneracy for the application of the Delort-Szeftel Theorem; That is why we need to treat it separately.

#### Application of Theorem 2.8

The application of Theorem 2.8 allows us to control quasi-resonances at arbitrary order via polynomial bounds, thus inducing a finite, but recoverable, loss of derivatives. The result we obtain is

##### Proposition 2.9

Fix $0<\beta _1<\beta _2<4\left( 2+\sqrt{3} \right)$, $\texttt{A}\in {\mathbb {N}}$ and $\texttt{M}\in {\mathbb {N}}^*$. Then, there exist $\nu _0,\tau ,\delta >0,$ depending on $\texttt{A}$ and $\texttt{M},$ such that for any $\tilde{\nu }\in (0,\nu _0),$ there exists a set $\mathcal {B}_{\tilde{\nu }}\subset \left[ \beta _1,\beta _2 \right]$ of measure $O(\tilde{\nu }^{\delta })$ such that for any $\beta \in \left[ \beta _1,\beta _2 \right] \setminus \mathcal {B}_{\tilde{\nu }},$ the following holds: denote $n_0=2$, $n_1=3$ and for any distinct integers $n_2,..., n_{\texttt{A}}\in \mathbb {N}\setminus \{2,3,5\}$ and any $\vec {c}=\left( c_0,c_1,...,c_{\texttt{A}} \right) \in {\mathbb {R}}^{\texttt{A}+1}\setminus \{0\}$ with $\max _{a=0,...,\texttt{A}}\left| c_a \right| \leqslant \texttt{M},$ we have

2.26 $$\begin{aligned} \left| \sum _{a=0}^{\texttt{A}}c_a\omega _{\gamma ,{\texttt{b}}}(n_a) \right| \geqslant \widetilde{\nu }\left( \sum _{a=0}^{\texttt{A}}n_a \right) ^{-\tau }. \end{aligned}$$

##### Proof

We denote $\vec {n}=\left( n_0,n_1,...,n_{\texttt{A}} \right) \in \left( {\mathbb {N}}^* \right) ^{\texttt{A}+1}$ with $n_2,...,n_{\texttt{A}}\not \in \{2,3,5\}$ and distinct, we introduce the notations

2.27 $$\begin{aligned} x_0\left( \vec {n} \right) \triangleq \left( \sum _{a=0}^{\texttt{A}}n_a \right) ^{-1},\qquad x_a\left( \vec {n} \right) \triangleq x_0\left( \vec {n} \right) \sqrt{n_a-1}. \end{aligned}$$

We note that

2.28 $$\begin{aligned} 0\leqslant |x_a\left( \vec {n} \right) |\leqslant 1, \quad \text {for any } a=0,\dots , \texttt{A}. \end{aligned}$$

The condition (2.26) is equivalent to

2.29 $$\begin{aligned} \left| f_{\vec {c}}(x,\beta ) \right| \geqslant \frac{\widetilde{\nu }}{\sqrt{\gamma }} x_0^{\tau +3}, \quad x=\left( x_0(\textbf{n} ),x_1(\textbf{n}),\dots , x_\texttt{A}(\textbf{n}) \right) \end{aligned}$$

where

2.30 $$\begin{aligned} f_{\textbf{c}}(x,\beta )\triangleq \sum _{a=1}^{\texttt{A}}r_a\lambda (x_a,x_0,\beta )+c_0\sqrt{3}x_0^3,\qquad x=(x_0,x_1,\dots , x_\texttt{A}) \in B_1\left( \mathbb {R}^{\texttt{A}+1} \right) , \end{aligned}$$

with

$$\begin{aligned}r_a\triangleq c_a\sqrt{x_a^2+x_0^2},\qquad \lambda (y,x_0,\beta )\triangleq \sqrt{y^4+\left( 2-\frac{\beta }{2} \right) x_0^2y^2+\frac{\beta }{2}x_0^4}.\end{aligned}$$

The function $f_{\textbf{c} }:B_1\left( \mathbb {R}^{\texttt{A}+1} \right) \times \left[ \beta _1,\beta _2 \right] \rightarrow \mathbb {R}$ is continuous and sub-analytic. Remark that, for any $l\in {\mathbb {N}}^*,$

2.31 $$\begin{aligned}&\partial _{\beta }^{l}\lambda (y,x_0,\beta )=\left( {\begin{array}{c}\frac{1}{2}\\ l\end{array}}\right) \mu ^{l}(y,x_0,\beta )\lambda (y,x_0,\beta ),\qquad \mu (y,x_0,\beta )\triangleq \nonumber \\&\quad \frac{x_0^2\left( x_0^2-y^2 \right) }{x_0^2\left( x_0^2-y^2 \right) \beta +2y^2\left( y^2+2x_0^2 \right) }\cdot \end{aligned}$$

Note that, since $0<{\beta }<\beta _2< 4(2+\sqrt{3})$, the denominator in (2.31) satisfies

2.32 $$\begin{aligned} x_0^2\left( x_0^2-y^2 \right) \beta +2y^2\left( y^2+2x_0^2 \right) >0\quad \text {for any } y\in [0,1], \ x_0\in (0,1].\nonumber \\ \end{aligned}$$

We introduce the polynomial function $\rho :[-1,1]^{\texttt{A}+1}\rightarrow {\mathbb {R}}$

2.33 $$\begin{aligned} \rho (x_0,x_1,...,x_{\texttt{A}})&\triangleq x_0\prod _{a=1}^{\texttt{A}}\left( x_a^2-x_0^2 \right) \prod _{1\leqslant a<b\leqslant \texttt{A}}\left( \left( x_0^2-x_a^2 \right) x_b^2\left( x_b^2+2x_0^2 \right) -\left( x_0^2-x_b^2 \right) x_a^2\left( x_a^2+2x_0^2 \right) \right) . \end{aligned}$$

The condition (2.32) implies that the function $f_{\textbf{c} }$ is real-analytic on $\{\rho \not =0\}\times \left[ \beta _1,\beta _2 \right]$. We fix now $\bar{x}=\left( \bar{x}_0, \dots \bar{x}_\texttt{A} \right)$ such that $\rho ( \bar{x} ) \not =0$. Then $\bar{x}_0\not = 0$ and, in view also of (2.32), one has

2.34 $$\begin{aligned} \mu \left( \bar{x}_a,\bar{x}_0,{\beta } \right) \not =\mu \left( \bar{x}_b,\bar{x}_0,{\beta } \right) , \quad \text {for any }\beta \in \left( 0,4(2+\sqrt{3}) \right) . \end{aligned}$$

The next Lemma ensures that the function $f_{\textbf{c}}(x,\beta )$ fulfills the second assumption of Delort-Szeftel Theorem 2.8.

##### Lemma 2.10

The solutions of the equation

$$\begin{aligned} f_{\vec {c}}\left( \bar{x}, \beta \right) =0, \end{aligned}$$

if any, are finitely many.

##### Proof

Since the function $\beta \rightarrow f_{\textbf{c}}\left( \bar{x}, \beta \right)$ is analytic for $\beta \in [\beta _1,\beta _2]$, we are only left to prove that it is not identically zero. To do so we fix $\bar{\beta }\in (\beta _1,\beta _2)$ and we note that

$$\begin{aligned}\forall l=1,...,\texttt{A},\quad \partial _{\beta }^lf\left( \bar{x},\bar{\beta } \right) =0\qquad \Leftrightarrow \qquad A\left( \bar{x} \right) \vec {r}=0,\end{aligned}$$

where $$\vec {r}\triangleq \left( r_1,...,r_{\texttt{A}} \right) $$ and

$$\begin{aligned}A\left( \bar{x} \right) \triangleq \begin{pmatrix} \mu \left( \bar{x}_1,\bar{x}_0,\bar{\beta } \right) \lambda \left( \bar{x}_1,\bar{x}_0,\bar{\beta } \right) &  ... &  \mu \left( \bar{x}_{\texttt{A}},\bar{x}_0,\bar{\beta } \right) \lambda \left( \bar{x}_{\texttt{A}},\bar{x}_0,\bar{\beta } \right) \\ \mu ^2\left( \bar{x}_1,\bar{x}_0,\bar{\beta } \right) \lambda \left( \bar{x}_1,\bar{x}_0,\bar{\beta } \right) &  ... &  \mu ^2\left( \bar{x}_{\texttt{A}},\bar{x}_0,\bar{\beta } \right) \lambda \left( \bar{x}_{\texttt{A}},\bar{x}_0,\bar{\beta } \right) \\ \vdots &  &  \vdots \\ \mu ^{\texttt{A}}\left( \bar{x}_1,\bar{x}_0,\bar{\beta } \right) \lambda \left( \bar{x}_1,\bar{x}_0,\bar{\beta } \right) &  ... &  \mu ^{\texttt{A}}\left( \bar{x}_{\texttt{A}},\bar{x}_0,\bar{\beta } \right) \lambda \left( \bar{x}_{\texttt{A}},\bar{x}_0,\bar{\beta } \right) \end{pmatrix}.\end{aligned}$$

Since $\textbf{c} \not =0$ and $\rho (\bar{x})\not =0$ we have also $\bar{x}_0 \not =0$ and $\vec {r}\ne 0.$ Then $\beta \mapsto f(\bar{x},\beta )\equiv 0$ implies that $\det \left( A(\bar{x}) \right) =0.$ Besides, by $\texttt{A}$-linearity of the determinant and recognizing a Vandermonde determinant, we find that

$$\begin{aligned}\det \left( A\left( \bar{x} \right) \right) =\prod _{a=1}^{\texttt{A}}\mu \left( \bar{x}_a,\bar{x}_0,\bar{\beta } \right) \lambda \left( \bar{x}_a,\bar{x}_0,\bar{\beta } \right) \prod _{1\leqslant a<b\leqslant \texttt{A}}\left( \mu \left( \bar{x}_a,\bar{x}_0,\bar{\beta } \right) -\mu \left( \bar{x}_b,\bar{x}_0,\bar{\beta } \right) \right) .\end{aligned}$$

By construction, since $\bar{x}\in \{\rho \ne 0\}$, one has that $\det \left( A\left( \bar{x} \right) \right) \ne 0$ at the given $\overline{\beta }.$ So $\beta \mapsto f(\bar{x},\beta )$ cannot be identically zero proving Lemma 2.10. $\square$

We thus conclude that there are $N_0 \in \mathbb {N}^*$, $\alpha _0, \delta , C >0$, such that, for any $\alpha \in (0, \alpha _0]$, any $N \in \mathbb {N}^*$, $N \geqslant N_0$, any $x\in X$ with $\rho (x) \ne 0$,

2.35 $$\begin{aligned} \left| \left\{ \beta \in \left[ \beta _1,\beta _2 \right] \quad \text {s.t.}\quad \left| f_{\textbf{c}} \, (x, \beta ) \right| \leqslant \alpha \left| \rho (x) \right| ^N \right\} \right| \leqslant C \alpha ^\delta \, \left| \rho (x) \right| ^{N\delta } \, . \end{aligned}$$

To conclude the proof we need the following:

##### Lemma 2.11

Let $$\tau _1\triangleq 2\texttt{A}+1+8\left( {\begin{array}{c}\texttt{A}\\ 2\end{array}}\right) .$$ Then

2.36 $$\begin{aligned} \left( \sum _{a=1}^{\texttt{A}}n_a \right) ^{-\tau _1}\lesssim \left| \rho \left( x(\vec {n}) \right) \right| \lesssim \left( \sum _{a=1}^{\texttt{A}}n_a \right) ^{-1}. \end{aligned}$$

##### Proof

By definition (2.27), we have

$$\begin{aligned}&\left( (x_0^2-x_a^2)x_b^2(x_b^2+2x_0^2)-(x_0^2 -x_b^2 )x_a^2 (x_a^2 +2x_0^2 ) \right) _{|x_0=x_0\left( \vec {n} \right) ,\, x_a=x_a\left( \vec {n} \right) ,\, x_b=x_b\left( \vec {n} \right) }\\&=x_0^8\left( \vec {n} \right) \left[ \left( 2-n_a \right) \left( n_b^2-1 \right) -\left( 2-n_b \right) \left( n_a^2-1 \right) \right] . \end{aligned}$$

The equation

$$\begin{aligned}\left( 2-n_a \right) \left( n_b^2-1 \right) =\left( 2-n_b \right) \left( n_a^2-1 \right) \end{aligned}$$

is equivalent to

2.37 $$\begin{aligned} \left( 2-n_a \right) \left( n_b-n_a \right) \left( n_b-\frac{2n_a-1}{n_a-2} \right) =0. \end{aligned}$$

Since $n_a\ne 2$ and $n_b\ne n_a,$ we must have $n_b=\frac{2n_a-1}{n_a-2}\cdot$ But

$$\begin{aligned}2n_a-1=2(n_a-2)+3.\end{aligned}$$

Hence

$$\begin{aligned}2n_a-1\equiv 3 \quad \text {mod.} \ [n_a-2].\end{aligned}$$

Therefore

$$\begin{aligned}n_a-2|2n_a-1\quad \Leftrightarrow \quad n_a\in \{3,5\}.\end{aligned}$$

We conclude that the only non-trivial couples of integers solutions to (2.37) are

$$\begin{aligned}(3,5)\qquad \text {and}\qquad (5,3).\end{aligned}$$

However, we have excluded the possibility $n_a=5$ or $n_b=5.$ This implies that the equation (2.37) is not solved with our choice of $\vec {n}$. Thus,

2.38 $$\begin{aligned}  &   \left| \left( \left( x_0^2-x_a^2 \right) x_b^2\left( x_b^2+2x_0^2 \right) -\left( x_0^2 -x_b^2 \right) x_a^2 \left( x_a^2 +2x_0^2 \right) \right) _{|x_0=x_0\left( \vec {n} \right) ,\, x_a=x_a\left( \vec {n} \right) ,\, x_b=x_b\left( \vec {n} \right) } \right| \geqslant x_0^8(\vec {n}).\nonumber \\\end{aligned}$$

Moreover, since $n_a\not =2$ for $a=1,\ldots , \texttt{A}$, one has that,

2.39 $$\begin{aligned} \left| x_a^2\left( \vec {n} \right) -x_0^2\left( \vec {n} \right) \right| \geqslant x_0^2\left( \vec {n} \right) . \end{aligned}$$

Gathering Equations (2.38) and (2.39) we obtain the lower bound in (2.36).

To prove the upper bound, we use the definition of $\rho$ given in (2.33) along with the bounds in (2.28). $\square$

Consider the set

2.40 $$\begin{aligned} \begin{aligned} \mathcal {B}(\alpha , N)&\triangleq \bigcup _{ \begin{array}{c} \vec {n}=(n_2, \ldots , n_\texttt{A}) \in \mathbb {N}^{\texttt{A}},\, 1 \leqslant n_1< \ldots < n_{\texttt{A}} \\ \textbf{c} \in (\mathbb {Z}^*)^{\texttt{A}}, \, |\textbf{c}|_{\infty } \leqslant \texttt{M} \end{array}}\mathcal {B}_{\textbf{c},\textbf{n}}(\alpha , N) \subset \left[ \beta _1,\beta _2 \right] , \\ \mathcal {B}_{\textbf{c},\textbf{n}}(\alpha , N)&\triangleq \left\{ \beta \in \left[ \beta _1,\beta _2 \right] \quad \text {s.t.}\quad \left| f_{\textbf{c}}\left( x(\textbf{n}), \beta \right) \right| \leqslant \alpha \left| \rho \left( x(\textbf{n}) \right) \right| ^N \right\} . \end{aligned} \end{aligned}$$

We fix now $\underline{N}\in \mathbb {N}$ such that $\bar{N} \delta > 1$ and $\mathcal {B}_\alpha \triangleq \mathcal {B}(\alpha , \underline{N} )$. Then we get that

2.41 $$\begin{aligned} \big | \mathcal {B}_\alpha \big | \leqslant C(\texttt{A}, \texttt{M}) \alpha ^\delta \sum _{n_2, \ldots , n_\texttt{A}\in \mathbb {N}} \left( \sum _{a=0}^\texttt{A}n_a\right) ^{-N\delta } \leqslant C'(\texttt{A}, \texttt{M}) \alpha ^\delta . \end{aligned}$$

In conclusion, for any $\beta \in \left[ \beta _1,\beta _2 \right] \setminus \mathcal {B}_ \alpha$, for any $\textbf{n} = (n_0,n_1, \ldots , n_\texttt{A})$ as in the hypothesis, any $\textbf{c} \in (\mathbb {Z}^*)^{\texttt{A}}$ with $|\textbf{c} \, |_{\infty } \leqslant \texttt{M}$, it results, by (2.40) and (2.36), that

2.42 $$\begin{aligned} \left| {f_{\textbf{c}} \, \left( x(\textbf{n}), \beta \right) } \right| > \alpha \left| \rho \left( x(\textbf{n}) \right) \right| ^{\underline{N}} \geqslant c(\texttt{A}) \alpha \left( \sum _{a=1}^\texttt{A}n_a\right) ^{-\tau _1 \underline{N} } . \end{aligned}$$

Recalling the definition of $f_{\textbf{c}}$ in (2.30) and $x_0(\textbf{n})$ in (2.27), the lower bound (2.42) implies (2.9) with $\tau \triangleq \tau _1 \underline{N} - 3$ (cfr. (2.29)) and re-denoting $\tilde{\nu } \triangleq \alpha c(\texttt{A})$. $\square$

#### Proof of Proposition 2.7

Fix a multi-index $\left( \alpha ,\alpha ' \right) \in ({\mathbb {N}}^*)^{{\mathbb {Z}}^*}\times ({\mathbb {N}}^*)^{{\mathbb {Z}}^*}$ of length $\left| \alpha +\alpha ' \right| \leqslant M.$ We denote $\mathfrak {N}\left( \alpha , \alpha ' \right)$ as in (2.23).

Since the couple $(\alpha ,\alpha ')$ is not super-action preserving, then

2.43 $$\begin{aligned} \mathfrak {N}(\alpha ,\alpha ')\ne \varnothing . \end{aligned}$$

Then, we can write that

$$\begin{aligned} \vec {\omega }_{\gamma , {\texttt{b}}}\cdot \left( \alpha -\alpha ' \right)&=\sum _{j\in {\mathbb {Z}}^*}\omega _{\gamma ,{\texttt{b}}}\left( \left| j \right| \right) \left( \alpha _j-\alpha _j' \right) =\sum _{n\in {\mathbb {N}}^*}\omega _{\gamma ,{\texttt{b}}}(n)\left( \alpha _{n}+\alpha _{-n}-\alpha _{n}'-\alpha _{-n}' \right) \\&=\sum _{n\in \mathfrak {N}\left( \alpha ,\alpha ' \right) }\omega _{\gamma ,{\texttt{b}}}(n)\left( \alpha _{n}+\alpha _{-n}-\alpha _{n}'-\alpha _{-n}' \right) . \end{aligned}$$

We use the notation

$$\begin{aligned}\texttt{A}\triangleq \left| \mathfrak {N}\left( \alpha ,\alpha ' \right) \setminus \{2,3,5\} \right| +1.\end{aligned}$$

We also denote that

$$\begin{aligned} \mathfrak {N}(\alpha ,\alpha ')\setminus \{2,3,5\}=\{n_2,...n_{\texttt{A}}\}. \end{aligned}$$

Therefore,

$$\begin{aligned} \vec {\omega }_{\gamma ,{\texttt{b}}}\cdot \left( \alpha -\alpha ' \right) =\sum _{a=2}^{\texttt{A}}c_a\omega _{\gamma ,{\texttt{b}}}(n_a)+c_0\omega _{\gamma ,{\texttt{b}}}(2)+c_{1}\omega _{\gamma ,{\texttt{b}}}(3), \end{aligned}$$

with

$$\begin{aligned}\begin{aligned}&c_0\triangleq \alpha _{2}+\alpha _{-2}-\alpha _2'-\alpha _{-2}', \\  &c_{1}\triangleq \left( \alpha _{3}+\alpha _{-3}-\alpha _{3}'-\alpha _{-3}' \right) +\sqrt{5}\left( \alpha _{5}+\alpha _{-5}-\alpha _{5}'-\alpha _{-5}' \right) \end{aligned}\end{aligned}$$

and for any $a=2,...,\texttt{A},$

$$\begin{aligned}c_a\triangleq \alpha _{n_a}+\alpha _{-n_a}-\alpha _{n_a}'-\alpha _{-n_a}'.\end{aligned}$$

Observe that for any $a=0,...,\texttt{A},$ $\left| c_a \right| \leqslant 4M$, where *M* is defined in Proposition 2.7. Let us assume the absurd hypothesis $\vec {c}\triangleq (c_0,c_{1},c_2,...,c_{\texttt{A}})=0.$ By definition, under such absurd hypothesis, we obtain that $\mathfrak {N}(\alpha ,\alpha ')\subset \{3,5\}.$ But $c_{1}\in {\mathbb {Z}}[\sqrt{5}]$ so $c_{1}=0$ implies also that $3,5\not \in \mathfrak {N}(\alpha ,\alpha ').$ Hence, $\mathfrak {N}(\alpha ,\alpha ')=\varnothing$ which is a contradiction with (2.43), thus we can safely assume $\textbf{c} \ne 0$. We consider the set

$$\begin{aligned}\mathcal {B}\triangleq \bigcap _{\tilde{\nu }\in (0,\nu _0)}\mathcal {B}_{\tilde{\nu }},\end{aligned}$$

where $\nu _0$ and $\mathcal {B}_{\tilde{\nu }}$ are introduced in Proposition 2.9. Hence, for any $\beta \in \left[ \beta _1,\beta _2 \right] \setminus \mathcal {B}$ there is $\tilde{\nu }\in (0,\nu _0)$ such that $\beta \in \left[ \beta _1,\beta _2 \right] \setminus \mathcal {B}_{\tilde{\nu }}$ and, applying Proposition 2.9 we get the desired result with $\nu \triangleq \frac{\tilde{\nu }}{M^{\tau }}$. $\square$

## Functional Setting

Throughout the document, we shall use the notations

$$\begin{aligned} {\mathbb {N}}\triangleq \{0,1,2,...\},  &   {\mathbb {N}}^*\triangleq {\mathbb {N}}\setminus \{0\},  &   \mathbb {Z}^*\triangleq \mathbb {Z}\setminus \{0\}. \end{aligned}$$

Along the paper we deal with real parameters

3.1 $$\begin{aligned} s\geqslant s_0 \gg K \gg \varrho \gg N {\geqslant }0, \end{aligned}$$

where $N \in \mathbb {N}$. The values of $s, s_0, K$ and $\varrho$ may vary from line to line while still being true the relation (3.1). We expand a $2\pi$-periodic function $u\in L^2 (\mathbb {T};\mathbb {C})$ in Fourier series as

$$\begin{aligned} u(x)= \sum _{j \in {\mathbb {Z}}} \hat{u}\left( j \right) e^{\textrm{i}j x},  &   \hat{u}\left( j \right) \triangleq \mathcal{F}_{x \rightarrow j}\left( j \right) \triangleq u_j \triangleq \int _{{\mathbb {T}}}u(x) e^{- \textrm{i}j x }\,\textrm{d}x . \end{aligned}$$

The function *u* is real-valued if and only if $\overline{u_j} = u_{-j}$, for any $j \in \mathbb {Z}$.

For any $s\in {\mathbb {R}},$ we define the Sobolev space $H^{s} \triangleq H^{s}(\mathbb {T};\mathbb {C})$ with norm

$$\begin{aligned} \left\| u \right\| _{s} \triangleq \left\| u \right\| _{H^{s}} = \left( \sum _{ j \in {\mathbb {Z}} } \left\langle j \right\rangle ^{2s} \left| \hat{u}\left( j \right) \right| ^2 \right) ^{\frac{1}{2}} ,  &   \left\langle j \right\rangle \triangleq \max \left\{ 1, \left| j \right| \right\} . \end{aligned}$$

We define $\Pi _0 u \triangleq u_0$ the average of *u* and

$$\begin{aligned} \Pi _0^\bot \triangleq \text {Id}- \Pi _0 . \end{aligned}$$

We define $H^s_0$ the subspace of zero average functions of $H^s$ for which we also denote $\left\| u \right\| _s = \left\| u \right\| _{H^s} = \left\| u \right\| _{H^s_0}$, and with ${\dot{H}}^s \triangleq \left. H^s / {\mathbb {C}} \right.$ endowed with the norm $\left\| u \right\| _{{\dot{H}}^s}\triangleq \left\| \Pi _0^\bot u \right\| _s$. We define, on $\dot{L}^2(\mathbb {T};\mathbb {C})\triangleq \dot{H}^0(\mathbb {T};\mathbb {C})$, the complex scalar product $\left\langle \cdot \ \bigg \vert \ \cdot \right\rangle _\mathbb {C}$ and the real symmetric bilinear form $\left\langle \cdot \ \bigg \vert \ \cdot \right\rangle _\mathbb {R}$ as follows: for any $u, v \in \dot{L}^2 (\mathbb {T};\mathbb {C})$,

3.2 $$\begin{aligned} \left\langle u \ \bigg \vert \ v \right\rangle _{ \mathbb {C}} \triangleq \int _\mathbb {T}\Pi _0^\bot u(x)\, \overline{\Pi _0^\bot { v(x)}} \, \textrm{d}x,  &   \left\langle u \ \bigg \vert \ v \right\rangle _{ \mathbb {R}} \triangleq \int _\mathbb {T}\Pi _0^\bot u(x)\, \Pi _0^\bot { v(x)} \, \textrm{d}x\,. \end{aligned}$$

Moreover we define the real subspace of $\dot{H}^s(\mathbb {T};\mathbb {C}^2)$

$$\begin{aligned} \dot{H}^s_\mathbb {R}(\mathbb {T};\mathbb {C}^2)\triangleq \left\{ U= \begin{bmatrix} u^+ \\ u^- \end{bmatrix} \in \dot{H}^s(\mathbb {T};\mathbb {C}^2)\quad \text {s.t.}\quad u^-= \overline{u^+}\right\} . \end{aligned}$$

We also denote

$$\begin{aligned} \dot{H}^{\infty }({\mathbb {T}};{\mathbb {C}}^2) \triangleq \bigcap _{s \in \mathbb {R}} \dot{H}^{s}({\mathbb {T}};{\mathbb {C}}^2),\qquad \dot{H}_{{\mathbb {R}}}^{\infty }({\mathbb {T}};{\mathbb {C}}^2) \triangleq \bigcap _{s \in \mathbb {R}} \dot{H}_{{\mathbb {R}}}^{s}({\mathbb {T}};{\mathbb {C}}^2).\end{aligned}$$

Given an interval $I\subset \mathbb {R}$ symmetric with respect to $t = 0$ and $s\in \mathbb {R}$, we define the space

$$\begin{aligned} C_*^K \left( I;{\dot{H}}^s\left( {\mathbb {T}};{\mathbb {C}}^2 \right) \right) \triangleq \bigcap _{k=0}^K C^k \left( I; {\dot{H}}^{s- \frac{3}{2} k}\left( {\mathbb {T}};{\mathbb {C}}^2 \right) \right) , \end{aligned}$$

endowed with the norm

3.3 $$\begin{aligned} \sup _{t\in I} \left\| U (t, \cdot ) \right\| _{K,s} \qquad \textrm{where} \qquad \left\| U(t, \cdot ) \right\| _{K,s}\triangleq \sum _{k=0}^K \left\| \partial _t^k U(t, \cdot ) \right\| _{\dot{H} ^{s- \frac{3}{2} k}} ; \end{aligned}$$

we also consider its subspace

$$\begin{aligned} C_{*\mathbb {R}}^K\left( I,{\dot{H}}^s\left( {\mathbb {T}},{\mathbb {C}}^2 \right) \right) \triangleq \left\{ U\in C_*^K\left( I,{\dot{H}}^s\left( {\mathbb {T}},{\mathbb {C}}^2 \right) \right) \quad \text {s.t.}\quad U=\begin{pmatrix} u \\ \bar{u}\end{pmatrix}\right\} .\end{aligned}$$

Given $r>0$ we set $B^K_s(I;r)$ the ball of radius *r* in $C_*^K\left( I,{\dot{H}}^s\left( {\mathbb {T}},{\mathbb {C}}^2 \right) \right)$ and by $B^K_{s,\mathbb {R}}(I;r)$ the ball of radius *r* in $C_{*\mathbb {R}}^K\left( I,{\dot{H}}^s\left( {\mathbb {T}},{\mathbb {C}}^2 \right) \right)$.

A vector field *X*(*u*) is *translation invariant* if

$$\begin{aligned} X \circ \textsf{t}_\varsigma = \textsf{t}_\varsigma \circ X,\qquad \forall \varsigma \in \mathbb {R}, \end{aligned}$$

where the translation operator $\textsf{t}_{\varsigma }$ is defined by

$$\begin{aligned} \textsf{t}_\varsigma :u(x) \mapsto u(x + \varsigma ). \end{aligned}$$

Given a linear operator $R(u) [ \cdot ]$ acting on $L^2_0(\mathbb {T};\mathbb {C})\triangleq H^0_0(\mathbb {T};\mathbb {C})$ we associate the linear operator defined by the relation $\overline{R(u)} v \triangleq \overline{R(u) \overline{v} }$ for any $v \in L^2_0(\mathbb {T};\mathbb {C}).$ An operator *R*(*u*) is *real * if $R(u) = \overline{R(u)}$ for any *u* real.

### Paradifferential Calculus

We introduce the para-differential calculus developed in [20, 61].

**Classes of symbols.** Roughly speaking the class $\widetilde{\Gamma }_p^m$ contains symbols of order *m* and homogeneity *p* in *u*, whereas the class $\Gamma _{K,K',p}^m$ contains non-homogeneous symbols of order *m* that vanish at degree at least *p* in *u* and that are $(K-K')$-times differentiable in *t*. We can think the parameter $K'$ like the number of time derivatives of *u* that are contained in the symbols.

#### Definition 3.1

*(Symbols)* Let $m\in \mathbb {R}$, $p,N\in \mathbb {N}$, $K, K' \in \mathbb {N}$ with $K' \leqslant K$, and $\epsilon _0>0$.

i) *p***-Homogeneous symbols.** We denote by $\widetilde{\Gamma }^m_p$ the space of *p*-linear symmetric maps from $\left( {\dot{H}}^{\infty }\left( {\mathbb {T}};\mathbb {\mathbb {C}}^2\right) \right) ^p$ to $C^\infty ({\mathbb {T}}\times \mathbb {R}; \mathbb {C})$ , $(x, \xi ) \mapsto a_p(U_1, \dots , U_p;x,\xi )$ whose associated polynomial has the form
  3.4 $$\begin{aligned} a_p(U;x,\xi )\triangleq a_p(U, \dots , U; x, \xi )\triangleq \sum _{\begin{array}{c} \vec {\jmath }\in (\mathbb {Z}^*)^p\\ \vec {\sigma }\in \{ \pm \}^p \end{array}} a_{\vec {\jmath }}^{\vec {\sigma }}(\xi ) u_{\vec {\jmath }}^{\vec {\sigma }} \, e^{\textrm{i}(\vec {\sigma }\cdot \vec {\jmath }) x}, \end{aligned}$$
  where $a_{\vec {\jmath }}^{\vec {\sigma }}(\xi )$ are complex valued Fourier multipliers, satisfying that
  $$\begin{aligned} a_{\vec {\jmath }}^{\vec {\sigma }}(\xi )\triangleq a_{j_1, \dots , j_p}^{\sigma _1, \dots , \sigma _p}(\xi ) = a_{j_{\pi (1)}, \dots , j_{\pi (p)}}^{\sigma _{\pi (1)}, \dots , \sigma _{\pi (p)}}(\xi ) \quad \text { for any } \pi \text { permutation of } \{1, \dots , p\}\,, \end{aligned}$$
  and for some $\mu \geqslant 0$,
  3.5 $$\begin{aligned} | \partial _\xi ^\beta a_{\vec {\jmath }}^{\vec {\sigma }}(\xi ) | \leqslant C_\beta \left\langle \vec {\jmath }\right\rangle ^{\mu } \langle \xi \rangle ^{m-\beta }, \quad \forall \vec {\jmath }\in \mathbb {Z}^p,\, \vec {\sigma }\in \{\pm \}^p, \, \beta \in \mathbb {N}. \end{aligned}$$
  We have used the following notations for given $\vec {\jmath }= (j_1,\dots ,j_p) \in \mathbb {Z}^p$ and $\vec {\sigma }=(\sigma _1,\dots ,\sigma _p)\in \{\pm \}^{p}$:
  $$\begin{aligned}\langle \vec {\jmath }\rangle \triangleq \max (1,|\vec {\jmath }|),\qquad |\vec {\jmath }|\triangleq \max (|j_1|, \ldots , |j_p| ),\qquad u_{\vec {\jmath }}^{\vec {\sigma }} \triangleq u_{j_1}^{\sigma _1} \dots u_{j_p}^{\sigma _p}.\end{aligned}$$
  Hence for a fixed $u\in {\mathbb {C}},$ we denote that
  $$\begin{aligned}u^{+}\triangleq u\qquad \text {and}\qquad u^{-}\triangleq \overline{u}.\end{aligned}$$
  We denote by $\widetilde{\Gamma }^m_0$ the space of constant coefficients symbols $\xi \mapsto a(\xi )$ which satisfy (3.5) with $\mu = 0$.
ii) **Non-homogeneous symbols. ** We denote by $\Gamma _{K,K',p}^m[\epsilon _0]$ the space of functions $a(U;t, x,\xi )$, defined for $U \in B^{K'}_{s_0}\left( I;\epsilon _0 \right)$ for some $s_0$ large enough, with complex values, such that for any $0\leqslant k\leqslant K-K'$, any $s\geqslant s_0$, there is $0<\epsilon _0(s)<\epsilon _0$ such that for any $\beta \in \mathbb {N}$ the following holds. There is $C\triangleq C_{s,\beta }>0$ such that for any $U\in B^{K'}_{s_0}\left( I;\epsilon _0(s) \right) \cap C_{*}^{k+K'}\left( I; \dot{H}^{s}\left( {\mathbb {T}};{\mathbb {C}} \right) \right)$ and $\alpha \in \mathbb {N}$, with $\alpha \leqslant s-s_0$ one has the estimate
  3.6 $$\begin{aligned} \left| \partial _t^k\partial _x^\alpha \partial _\xi ^\beta a\left( U;t, x,\xi \right) \right| \leqslant C \langle \xi \rangle ^{m-\beta } \Vert U \Vert _{k+K',s_0}^{p-1}\Vert U\Vert _{k+K',s} \, . \end{aligned}$$
  If $p = 0$ the right hand side has to be replaced by $C \langle \xi \rangle ^{m-\beta }$. We say that a non-homogeneous symbol $a(U;x,\xi )$ is *real* if it is real valued for any $U \in B^{K'}_{s_0,\mathbb {R}}\left( I;\epsilon _0 \right)$.
iii) **Symbols.** We denote by $\Sigma \Gamma _{K,K',p}^m[\epsilon _0,N]$ the space of symbols
  3.7 $$\begin{aligned} a(U;t, x,\xi )= \sum _{q=p}^{N} a_q\left( U;x,\xi \right) + a_{>N}(U;t, x,\xi ), \end{aligned}$$
  where $$a_q $$, $$q=p,\dots , N$$ are homogeneous symbols in $$ \widetilde{\Gamma }_q^m $$ and $$a_{>N} $$ is a non-homogeneous symbol in $$ \Gamma _{K,K',N+1}^m $$. We say that a symbol $$a(U;t, x,\xi ) $$ is *real* if it is real valued for any $$ U \in B^{K'}_{s_0,\mathbb {R}}(I;\epsilon _0)$$. We shall also denote with $$ \Sigma _p^N \Gamma ^m_q$$ the subspace of $$\Sigma \Gamma _{K,K',p}^m[\epsilon _0,N]$$ made of pluri-homogeneous symbols, namely symbols which expand as in (3.7) with $$a_{>N}\equiv 0$$.

#### Remark 3.2

Let us make the following remarks:

- Given a *p*-homogeneous symbol $a_p \in \widetilde{\Gamma }^{m}_p$, we shall often, with a slight abuse of notation, identify the associated *p*-homogeneous polynomial with the *p*-linear symbol itself
  $$\begin{aligned} a_p(U_1, \ldots , U_p; x, \xi )\quad \leftrightsquigarrow \leftrightsquigarrow \quad a_p(U; x, \xi )\triangleq a_p(U, \ldots , U; x, \xi ). \end{aligned}$$
  This identification is harmless due to the standard correspondence between *p*-homogeneous polynomials and symmetric *p*-linear maps.
- If $a ( U; \cdot )$ is a homogeneous symbol in ${\widetilde{\Gamma }}_p^m$ then it belongs to the class of non-homogeneous symbols $\Gamma ^m_{K,0,p} [\epsilon _0]$, for any $\epsilon _0 > 0$.
- The classical properties expected for a symbol hold: if *a* is a symbol in $\Sigma \Gamma ^m_{K,K',p}[\epsilon _0,N]$ then $\partial _x a$ is in $\Sigma \Gamma ^{m}_{K,K',p}[\epsilon _0,N]$ and $\partial _\xi a$ belongs to $\Sigma \Gamma ^{m-1}_{K,K',p}[\epsilon _0,N]$. If in addition *b* is a symbol in $\Sigma \Gamma ^{m'}_{K,K',p'}[\epsilon _0,N]$ then their product *ab* is a symbol in $\Sigma \Gamma ^{m+m'}_{K,K',p+p'}[\epsilon _0,N]$.

We also define classes of functions in analogy with our classes of symbols.

#### Definition 3.3

*(Functions)* Let $p, N \in \mathbb {N}$, $K,K'\in \mathbb {N}$ with $K'\leqslant K$, $\epsilon _0>0$. We denote by $\tilde{\mathcal {F}}_{p}$, resp. $\mathcal {F}_{K,K',p}[\epsilon _0]$, $\Sigma \mathcal {F}_{K,K',p}[\epsilon _0,N]$, the subspace of $\tilde{\Gamma }^{0}_{p}$, resp. $\Gamma ^0_{K,K',p}[\epsilon _0]$, resp. $\Sigma \Gamma ^{0}_{K,K',p}[\epsilon _0,N]$, made of those symbols which are independent of $\xi$. We write $\tilde{\mathcal {F}}^{\mathbb {R}}_{p}$, resp. $\mathcal {F}_{K,K',p}^{\mathbb {R}}[\epsilon _0]$, $\Sigma \mathcal {F}_{K,K',p}^{\mathbb {R}}[\epsilon _0,N]$, to denote functions in $\tilde{\mathcal {F}}_{p}$, resp. $\mathcal {F}_{K,K',p}[\epsilon _0]$, $\Sigma \mathcal {F}_{K,K',p}[\epsilon _0,N]$, which are real valued for any $u \in B^{K'}_{s_0}\left( I;\epsilon _0 \right)$.

**Paradifferential quantization.** Given $p\in \mathbb {N},$ we consider functions $\chi _{p}\in C^{\infty }(\mathbb {R}^{p}\times \mathbb {R};\mathbb {R})$ and $\chi \in C^{\infty }(\mathbb {R}\times \mathbb {R};\mathbb {R})$, even with respect to each of their arguments, satisfying, for $0<\delta _0\leqslant \tfrac{1}{10}$,

3.8 $$\begin{aligned} \begin{aligned}&\mathrm{{supp}}\, \chi _{p} \subset \{(\xi ',\xi )\in \mathbb {R}^{p}\times \mathbb {R}\quad \text {s.t.}\quad |\xi '|\leqslant \delta _0 \langle \xi \rangle \} ,\qquad \chi _p (\xi ',\xi )\equiv 1\,\,\, \textrm{ for } \,\,\, |\xi '|\leqslant \tfrac{1}{2}\delta _0 \langle \xi \rangle , \\&\textrm{supp}\, \chi \subset \{(\xi ',\xi )\in \mathbb {R}\times \mathbb {R}\quad \text {s.t.}\quad |\xi '|\leqslant \delta _0 \langle \xi \rangle \} ,\qquad \quad \chi (\xi ',\xi ) \equiv 1\,\,\, \textrm{ for } \,\,\, |\xi '|\leqslant \tfrac{1}{2}\delta _0 \langle \xi \rangle . \end{aligned} \end{aligned}$$

For $p=0,$ we set $\chi _0\equiv 1$. We assume moreover, that

$$\begin{aligned} |\partial _{\xi }^{\ell }\partial _{\xi '}^{\beta }\chi _p(\xi ',\xi )|&\leqslant C_{\ell ,\beta }\langle \xi \rangle ^{-\ell -|\beta |},\quad \forall \ell \in \mathbb {N}, \,\beta \in \mathbb {N}^{p},\\ |\partial _{\xi }^{\ell }\partial _{\xi '}^{\beta }\chi (\xi ',\xi )|&\leqslant C_{\ell ,\beta }\langle \xi \rangle ^{-\ell -\beta }, \quad \forall \ell , \,\beta \in \mathbb {N}. \end{aligned}$$

If $a (x, \xi )$ is a smooth symbol we define its Weyl quantization as the operator acting on a $2 \pi$-periodic function *u* as

$$\begin{aligned} \textrm{Op}^{W}(a)u(x)= \sum _{k\in \mathbb {Z}} \Big (\sum _{j\in \mathbb {Z}}\hat{a}\big (k-j, \tfrac{k+j}{2}\big ) u_j \Big ){e^{\textrm{i}k x}}, \end{aligned}$$

where $\hat{a}(k,\xi )$ is the $k^{th}-$Fourier coefficient of the $2\pi -$periodic function $x\mapsto a(x,\xi )$.

#### Definition 3.4

*(Bony-Weyl quantization)* If $a(U;x,\xi )$ is a symbol in $\widetilde{\Gamma }^{m}_{p}$, and respectively, in $\Gamma ^{m}_{K,K',p}[\epsilon _0]$, we set that

$$\begin{aligned} \begin{aligned} a_{\chi _{p}}(U;x,\xi )&\triangleq \!\!\!\!\! \sum _{\begin{array}{c} \vec {\jmath }\in (\mathbb {Z}^*)^p\\ \vec {\sigma }\in \{ \pm \}^p \end{array}}\chi _p (\vec {\jmath },\xi ) a_{\vec {\jmath }}^{\vec {\sigma }}(\xi ) u_{\vec {\jmath }}^{\vec {\sigma }} e^{\textrm{i}(\vec {\sigma }\cdot \vec {\jmath }) x}, \\ a_{\chi }(U;x,\xi )&\triangleq \sum _{j\in \mathbb {Z}^*} \chi (j,\xi )\hat{a}(U;j,\xi )e^{\textrm{i}j x} , \end{aligned} \end{aligned}$$

where in the last equality $\hat{a}(U; j,\xi )$ stands for $j^{th}$ Fourier coefficient of $a(U;x, \xi )$ with respect to the *x* variable, and we define the *Bony-Weyl* quantization of $a(U; \cdot )$ as

3.9 $$\begin{aligned}&{{{\,\mathrm{Op^{BW}}\,}}\left( a({U};\cdot )\right) }v(x)\triangleq \textrm{Op}^{W} (a_{\chi _{p}}({U};\cdot ))v(x)\nonumber \\&\quad = \!\!\!\!\!\!\!\!\!\sum _{\begin{array}{c} (\vec {\jmath },j,k)\in (\mathbb {Z}^*)^{p+2}\\ \vec {\sigma }\in \{ \pm \}^p\\ \vec {\sigma }\cdot \vec {\jmath }+j=k \end{array}}\chi _p \left( \vec {\jmath },\frac{j+k}{2}\right) a_{\vec {\jmath }}^{\vec {\sigma }}\left( \frac{j+k}{2}\right) u_{\vec {\jmath }}^{\vec {\sigma }} v_j {e^{\textrm{i}k x}} \, , \end{aligned}$$

3.10 $$\begin{aligned}&{{{\,\mathrm{Op^{BW}}\,}}\left( a(U;\cdot )\right) }v(x)\triangleq \textrm{Op}^{W} (a_{\chi }(U;\cdot ))v(x)\nonumber \\&\quad = \!\!\!\!\!\! \sum _{(j,k)\in (\mathbb {Z}^*)^2} \!\!\! \chi \left( k-j,\frac{j+k}{2} \right) \hat{a}\left( U; k-j, \frac{k+j}{2}\right) v_j {e^{\textrm{i}k x}} \, . \end{aligned}$$

Note that if $\chi \Big ( k-j, \tfrac{ k + j }{2}\Big ) \ne 0$ then $|k-j| \leqslant \delta _0 \langle \frac{j + k}{2} \rangle$ and therefore, for $\delta _0 \in (0,1)$,

$$\begin{aligned} \frac{1-\delta _0}{1+\delta _0} |k| \leqslant |j| \leqslant \frac{1+\delta _0}{1-\delta _0}|k| \, , \quad \forall j, k \in \mathbb {Z}\, . \end{aligned}$$

This relation shows that the action of a paradifferential operator does not spread much the Fourier support of functions.

If *a* is a symbol in $\Sigma \Gamma ^{m}_{K,K',p}[\epsilon _0,N]$, we define its *Bony-Weyl* quantization

$$\begin{aligned} {{{\,\mathrm{Op^{BW}}\,}}\left( a(U;\cdot )\right) } =\sum _{q=p}^{N} {{{\,\mathrm{Op^{BW}}\,}}\left( a_q(U;\cdot )\right) } + {{{\,\mathrm{Op^{BW}}\,}}\left( a_{>N}(U; \cdot ) \right) } . \end{aligned}$$

We define, as well that

3.11 $$\begin{aligned}&{{\,\mathrm{Op^{BW}_{vec}}\,}}\left( a\left( U;x,\xi \right) \right) \triangleq {{{\,\mathrm{Op^{BW}}\,}}\left( \begin{bmatrix} a\left( U;x, \xi \right) &  0 \\ 0 &  \overline{a^\vee \left( U;x, \xi \right) } \end{bmatrix} \right) } , \nonumber \\&a^\vee \left( U;x, \xi \right) \triangleq a\left( U;x, -\xi \right) . \end{aligned}$$

#### Remark 3.5

- The operator ${{{\,\mathrm{Op^{BW}}\,}}\left( a\right) }$ maps functions with zero average in functions with zero average, and $\Pi _0^\bot {{{\,\mathrm{Op^{BW}}\,}}\left( a\right) } = {{{\,\mathrm{Op^{BW}}\,}}\left( a\right) }\Pi _0^\bot$.
- If *a* is a homogeneous symbol, the two definitions of quantization in (3.9)-(3.10) differ by a smoothing operator according to Definition 3.11 below, see [61, page 50].
- Definition 3.4 is independent of the cut-off functions $\chi _{p}$, $\chi$, up to smoothing operators (Definition 3.11).
- The action of ${{{\,\mathrm{Op^{BW}}\,}}\left( a\right) }$ on the spaces $\dot{H}^s$ only depends on the values of the symbol $a(u;t, x,\xi )$ for $|\xi |\geqslant 1$. Therefore, we may identify two symbols $a(u;t, x,\xi )$ and $b(u;t, x,\xi )$ if they agree for $|\xi | \geqslant 1/2$. In particular, whenever we encounter a symbol that is not smooth at $\xi =0$, such as, for example, $a = g(x)|\xi |^{m}$ for $m\in \mathbb {R}^*$, or ${{\,\textrm{sgn}\,}}{\xi }$, we will consider its smoothed out version $\left( 1-\chi (\xi ) \right) a\left( x, \xi \right)$, where $\chi$ is defined in (3.8). Similarly for *p*-homogeneous symbols.

#### Remark 3.6

Given a paradifferential operator $A = {{{\,\mathrm{Op^{BW}}\,}}\left( a(x,\xi )\right) }$ it turns out that

$$\begin{aligned} \overline{ A} = {{{\,\mathrm{Op^{BW}}\,}}\left( \overline{a(x, - \xi )}\right) }, \qquad A^\intercal = {{{\,\mathrm{Op^{BW}}\,}}\left( a(x, - \xi )\right) }, \qquad A^*= {{{\,\mathrm{Op^{BW}}\,}}\left( \overline{a(x, \xi )}\right) }, \end{aligned}$$

where $A^\intercal$ is the transposed operator with respect to the real scalar product $\left\langle \cdot \ \bigg \vert \ \cdot \right\rangle _\mathbb {R}$ in (3.2), and $A^*$ denotes the adjoint operator with respect to the complex scalar product $\left\langle \cdot \ \bigg \vert \ \cdot \right\rangle _\mathbb {C}$ on ${\dot{L}}^2$ in (3.2). This results in $A^* = \overline{A}^\intercal$.

- A paradifferential operator $A= {{{\,\mathrm{Op^{BW}}\,}}\left( a(x,\xi )\right) }$ is *real* (i.e. $A = \overline{A}$) if
  3.12 $$\begin{aligned} \overline{a(x,\xi )}= a(x,-\xi ) \, . \end{aligned}$$
- It is *symmetric* (i.e. $A = A^\intercal$) if
  $$\begin{aligned}a(x,\xi ) = a(x,-\xi ). \end{aligned}$$

We now provide the action of a paradifferential operator on Sobolev spaces, cf. [61, Prop. 3.8].

#### Lemma 3.7

(Action of a paradifferential operator) Let $m \in \mathbb {R}$.

i) If $p \in \mathbb {N}$, there is $s_0 > 0$ such that for any symbol *a* in ${\tilde{\Gamma }^{m}_{p}}$, there is a constant $C > 0$, depending only on *s* and on (3.5) with ${\texttt{b}}= \beta = 0$, such that, for any $(U_1, \ldots , U_p )$, for $p \geqslant 1$,
  $$\begin{aligned} \left\| {{{\,\mathrm{Op^{BW}}\,}}\left( a(U_1, \ldots , U_p;\cdot )\right) } u_{p+1} \right\| _{\dot{H}^{s-m}}\leqslant C \left\| U_1 \right\| _{\dot{H}^{s_0}} \cdots \left\| U_p \right\| _{\dot{H}^{s_0}} \left\| u_{p+1} \right\| _{\dot{H}^{s}} . \end{aligned}$$
  If $p = 0$ the above bound holds replacing the right hand side with $C \left\| u_{p+1} \right\| _{\dot{H}^{s}}$.
ii) Let $\epsilon _0>0$, $p\in \mathbb {N}$, $K'\leqslant K\in \mathbb {N}$, *a* in ${{\Gamma }^{m}_{K,K', p}[\epsilon _0]}$. There is $s_0 > 0$, and a constant *C*, depending only on *s*, $\epsilon _0$, and on (3.6) with $0 \leqslant \alpha \leqslant 2, \beta = 0$, such that, for any *t* in *I*, any $0\leqslant k\leqslant K-K'$, any *U* in ${B^{K}_{{s_0}_{}}(I;\epsilon _0)}$,
  3.13 $$\begin{aligned} \left\| {{{\,\mathrm{Op^{BW}}\,}}\left( \partial _t^ka(U;t,\cdot ) \right) } \right\| _{{\mathcal {L}}\left( \dot{H}^{s},\dot{H}^{s-m} \right) }\leqslant C \Vert U(t, \cdot )\Vert _{k+K',s_0}^p \, , \end{aligned}$$
  so that $$\left\| {{{\,\mathrm{Op^{BW}}\,}}\left( a(U;t,\cdot )\right) } v(t) \right\| _{K-K', s-m} \leqslant C \Vert U(t, \cdot )\Vert _{K,s_0}^p \Vert v(t) \Vert _{K- K', s} $$.

**Classes of ***m***-Operators and smoothing Operators.** Given integers $\left( n_1,\ldots ,n_{p+1} \right) \in (\mathbb {N}^*)^{p+1}$ we denote by $\max _{2}\left\{ n_1,\ldots , n_{p+1} \right\}$ the second largest among $n_1,\ldots , n_{p+1}$. We now define *m*-operators, which include the class of paradifferential operators of order *m* (see Remark 3.12), and allow for definition of define the smoothing remainders when the order *m* is negative (see Definition 3.11). The class $\tilde{\mathcal {M}}^{m}_{p}$ denotes multilinear operators that lose *m* derivatives and are *p*-homogeneous in *u*, while the class $\mathcal {M}_{K,K',p}^{m}$ contains non-homogeneous operators which lose *m* derivatives, vanish at degree at least *p* in *u*, satisfy tame estimates and are $(K-K')$-times differentiable in *t*. The constant $\mu$ in (3.15) takes into account possible loss of derivatives in the “low" frequencies.

The following definition is taken from [20, Def. 2.5] (see also its Fourier characterization in [20, Lemma 2.9]:

#### Definition 3.8

*(Classes of* *m-operators)* Let $m \in \mathbb {R}$, $p,N\in \mathbb {N}$, $K,K'\in \mathbb {N}$ with $K'\leqslant K$, and $\epsilon _0 > 0$.

i) *p***-homogeneous** *m***-operators.** We denote by $$\tilde{\mathcal {M}}^{m}_{p}$$ the space of $$ (p+1)$$-linear symmetric translation invariant operators from $$ \left( \dot{H}^\infty \left( {\mathbb {T}};{\mathbb {C}}^2 \right) \right) ^{p} \times \dot{H}^\infty \left( {\mathbb {T}};{\mathbb {C}} \right) $$ to $$ \dot{H}^\infty \left( {\mathbb {T}};{\mathbb {C}} \right) $$, whose associated polynomial has the form
  3.14 $$\begin{aligned} M(U)v \triangleq M\left( U, \ldots , U \right) v = \sum _{\begin{array}{c} \mathbf {\sigma }\in \{\pm \}^{p} \\ k -j = \mathbf {\sigma }\cdot \mathbf {\jmath } \end{array} } \!\!\!\!\!\! M_{\mathbf {\jmath }, j,k}^{\mathbf {\sigma }} \, u_{\mathbf {\jmath }}^{\mathbf {\sigma }}\, v_{j} \, {e^{\textrm{i}k x} } \end{aligned}$$
  with coefficients $M_{\mathbf {\jmath }, j,k}^{\mathbf {\sigma }}$ symmetric in $(j_1,\sigma _1), \ldots , (j_{p},\sigma _p)$, satisfying the following: there are $\mu {\geqslant }0$, $C>0$ such that, for any $\mathbf {\jmath }=( j_1, \ldots , j_{p}) \in (\mathbb {Z}^*)^p$, $j, k \in \mathbb {Z}^*$,
  3.15 $$\begin{aligned} \left| M_{\mathbf {\jmath }_{p}, j,k}^{\mathbf {\sigma }_{p}} \right| \leqslant C \ \textrm{max} _2 \left\{ \left| j_1 \right| , \ldots , \left| j_{p} \right| , \left| j \right| \right\} ^\mu \ \max \left\{ \left| j_1 \right| , \ldots , \left| j_{p} \right| ,\left| j \right| \right\} ^m , \end{aligned}$$
  If $p=0$ the right hand side of (3.14) must be substituted with $\sum _{j\in \mathbb {Z}} M_j v_j e^{\textrm{i}j x}$ with $\left| M_j \right| \leqslant C \left| j \right| ^m$.
ii) **Non-homogeneous** *m***-operators.** We denote by $\mathcal {M}^{m}_{K,K',p}[\epsilon _0]$ the space of operators $(U,t,v)\mapsto M(U;t) v$ defined on $B^{K'}_{s_0}\left( I;\epsilon _0 \right)$ for some $s_0 >0$, which are linear in the variable *v* and such that the following holds true. For any $s\geqslant s_0$ there are $C>0$ and $\epsilon _0(s)\in ]0,\epsilon _0[$ such that for any $U \in B^{K'}_{s_0}\left( I;\epsilon _0 \right) \cap C^K_{*}\left( I,\dot{H}^{s}(\mathbb {T};\mathbb {C}) \right)$, any $v \in C^{K-K'}_{*} \left( I,\dot{H}^{s}(\mathbb {T};\mathbb {C}) \right)$, any $0\leqslant k\leqslant K-K'$, $t\in I$, we have that
  3.16 $$\begin{aligned}&\left\| {\partial _t^k\left( M(U;t)v\right) } \right\| _{s- \frac{3}{2} k-m}\nonumber \\&\quad \leqslant C \sum _{k'+k''=k} \left( \Vert {v}\Vert _{k'',s}\Vert {U}\Vert _{k'+K',{s_0}}^{p} +\Vert {v}\Vert _{k'',{s_0}}\Vert U\Vert _{k'+K',{s_0}}^{p-1}\Vert U \Vert _{k'+K',s} \right) . \end{aligned}$$
  In case $$ p = 0$$ we require the estimate $$ \Vert {\partial _t^k\left( M(U;t) v \right) }\Vert _{s- \frac{3}{2}k-m} \leqslant C \Vert v \Vert _{k,s}$$. We say that a non-homogeneous *m*-operator $$M\left( U;t \right) $$ is *real* if it is real valued for any $$ u \in B^{K'}_{s_0}\left( I;\epsilon _0 \right) $$.
iii) *m***-Operators.** We denote by $\Sigma \mathcal {M}^{m}_{K,K',p}[\epsilon _0,N]$ the space of operators
  3.17 $$\begin{aligned} M(U;t)v =\sum _{q=p}^{N}M_{q}(U)v+M_{>N}(U;t)v , \end{aligned}$$
  where $$M_{q} $$ are homogeneous *m*-operators in $$\tilde{\mathcal {M}}^{m}_{q}$$, $$q=p,\ldots , N$$ and $$M_{>N}$$ is a non-homogeneous *m*-operator in $$\mathcal {M}^{m}_{K,K',N+1}[\epsilon _0]$$. We say that a *m*-operator $$M\left( u;t \right) $$ is *real* if it is real valued for any $$ u \in B^{K'}_{s_0}\left( I;\epsilon _0 \right) $$. We shall also denote with $$ \Sigma _p^N \widetilde{\mathcal {M}}^m_q$$ the subspace of $$\Sigma \mathcal {M}_{K,K',p}^m[\epsilon _0,N]$$ made of pluri-homogeneous *m*-operators, namely symbols which expand as in (3.7) with $$M_{>N}\equiv 0$$.

#### Remark 3.9

By [20, Lemma 2.8], if $M( U_1,\dots , U_p)$ is a *p*–homogeneous *m*-operator in ${\widetilde{\mathcal {M}}}_p ^m$ then $M(U) = M(U, \ldots , U)$ is a non-homogeneous *m*-operator in $\mathcal{M}^m_{K,0,p}[\epsilon _0]$ for any $\epsilon _0>0$ and $K\in \mathbb {N}$.

#### Notation 3.10

- If $M(U_1, \ldots , U_p)$ is a *p*-homogeneous *m*-operator, we shall often denote by *M*(*U*) the associated *p*-homogeneous polynomial, as in (3.14), and write $M(U) \in \widetilde{\mathcal {M}}_p^m$. Conversely, a *p*-homogeneous polynomial can be represented by a $(p+1)$-linear form of the type $M(U_1, \ldots , U_p) U_{p+1}$, which may not be symmetric in the first *p* variables. If this form satisfies the symmetric estimate (3.15), then it corresponds to an *m*-operator in $\widetilde{\mathcal {M}}_p^m$ obtained by symmetrizing the internal variables. In the sequel, we adopt this identification without further comment.
- given an operator *M*(*U*; *t*) in $\Sigma \mathcal {M}_{K,K',p}^m[r, N]$ of the form (3.17), we denote by
  3.18 $$\begin{aligned} \mathcal {P}_{\leqslant N}[ M(U;t)] \triangleq \sum _{q=p}^{N} M_q(U) \, , \quad \text {resp.} \qquad \mathcal {P}_{q}[ M(U;t)] \triangleq M_q(U) \, \end{aligned}$$
  the projections on the pluri-homogeneous, resp. homogeneous, operators in $\Sigma _p^N {\widetilde{\mathcal {M}}}^m_q$ , resp. in $\widetilde{\mathcal {M}}_q^m$. Given an integer $p\leqslant p'\leqslant N$ we also denote that
  $$\begin{aligned} \mathcal {P}_{\geqslant p'}[ M(U;t)] \triangleq \sum _{q=p'}^{N} M_q(U), \qquad \mathcal {P}_{\leqslant p'}[ M(U;t)] \triangleq \sum _{q=p}^{p'} M_q(U) \, . \end{aligned}$$
  The same notation will be also used to denote pluri-homogeneous/homogeneous components of symbols.

If $m \leqslant 0$ the operators in $\Sigma \mathcal {M}^{m}_{K,K',p}[\epsilon _0,N]$ are referred to as smoothing operators.

#### Definition 3.11

*(Smoothing operators)* Let $\varrho {\geqslant }0$. A $(-\varrho )$-operator *R*(*U*) belonging to $\Sigma \mathcal {M}^{-\varrho }_{K,K',p}[\epsilon _0,N]$ is called a smoothing operator. We also denote that

$$\begin{aligned}&\widetilde{\mathcal {R}}^{-\varrho }_{p}\triangleq \widetilde{\mathcal {M}}^{-\varrho }_{p} \, , \quad \mathcal {R}^{-\varrho }_{K,K',p}[\epsilon _0]\triangleq \mathcal {M}^{-\varrho }_{K,K',p}[\epsilon _0] \, ,\\&\Sigma \mathcal {R}^{-\varrho }_{K,K',p}[\epsilon _0,N]\triangleq \Sigma \mathcal {M}^{-\varrho }_{K,K',p}[\epsilon _0,N] \,, \quad \Sigma _p^N \widetilde{\mathcal {R}}^{-\varrho }_q\triangleq \Sigma _p^N \widetilde{\mathcal {M}}^{-\varrho }_q . \end{aligned}$$

#### Remark 3.12

$\bullet$ Lemma 3.7 implies that, if $a(U; t, \cdot )$ is a symbol in $\Sigma \Gamma ^{m}_{K,K',p}\left[ \epsilon _0, N \right]$, $m\in {\mathbb {R}}$, then the associated paradifferential operator ${{{\,\mathrm{Op^{BW}}\,}}\left( a(U; t, \cdot )\right) }$ defines a *m*-operator in $\Sigma \mathcal {M}^{m}_{K,K',p}\left[ \epsilon _0, N \right]$.

$\bullet$ The composition of smoothing operators $R_1 \in {{\Sigma {\mathcal {R}}}^{-\varrho }_{K,K',p_1}[\epsilon _0 ,N]}$ and $R_2 \in {{\Sigma {\mathcal {R}}}^{-\varrho }_{K,K',p_2}[\epsilon _0 ,N]}$ is a smoothing operator $R_1 R_2$ in ${{\Sigma {\mathcal {R}}}^{-\varrho }_{K,K',p_1+p_2}[\epsilon _0 ,N]}$. This is a particular case of Proposition 3.17-(*i*) below.

#### Definition 3.13

*(Homogeneous vector fields)* Let $$m \in \mathbb {R}$$ and $$ p, N \in \mathbb {N}$$. We denote by $$\widetilde{{\mathfrak X }}_{p+1}^m$$ the space of $$ (p+1)$$-homogeneous vector fields of the form $$ X(U)=M(U)U$$ where *M*(*U*) is a matrix of *p*-homogeneous *m*-operators in . In particular, one has the Fourier expansion

$$\begin{aligned}X(U)=\begin{bmatrix} X(U)^+\\ X(U)^- \end{bmatrix},\qquad X(U)^{\sigma }\triangleq \sum _{ \begin{array}{c} (\vec {\jmath }, k, \vec {\sigma }, - \sigma ) \in {\mathfrak {T}}_{p+2} \end{array}}X_{\vec {\jmath },k}^{\vec {\sigma },\sigma } u_{\vec {\jmath }}^{\vec {\sigma }}e^{\textrm{i}\sigma k},\end{aligned}$$

where the Fourier restriction $\mathfrak {T}_{p+2}$ is the set of momentum preserving indices, defined, for a given $q\in \mathbb {N}^*$, as

3.19 $$\begin{aligned} \mathfrak {T}_q\triangleq \left\{ \left( \vec {\jmath } , \mathbf {\sigma } \right) \in \mathbb {Z}^q \times \{\pm \}^q \quad \text {s.t.}\quad \mathbf {\sigma }\cdot \vec {\jmath } = 0 \right\} . \end{aligned}$$

We denote $\Sigma _{p+1}^{N+1}\widetilde{{\mathfrak X }}^{m}_q$ the class of pluri-homogeneous vector fields. The vector fields in $\widetilde{{\mathfrak X }}_{p+1}^{- \varrho }$, $\varrho \geqslant 0$, are called smoothing.

**Symbolic calculus.** Let ${\sigma }(D_{x},D_{\xi },D_{y},D_{\eta }) \triangleq D_{\xi }D_{y}-D_{x}D_{\eta }$ where $D_{x}\triangleq \frac{1}{\textrm{i}}\partial _{x}$ and $D_{\xi },D_{y},D_{\eta }$ are similarly defined. The following is Definition 3.11 in [61]:

#### Definition 3.14

*(Asymptotic expansion of composition symbol)* Let *p*, $p'$ in $\mathbb {N}$, $K, K' \in \mathbb {N}$ with $K'\leqslant K$, $\varrho {\geqslant }0$, $m,m'\in \mathbb {R}$, $\epsilon _0>0$. Consider symbols $a \in \Sigma \Gamma _{K,K',p}^{m}[\epsilon _0,N]$ and $b\in \Sigma \Gamma ^{m'}_{K,K',p'}[\epsilon _0,N]$. For *U* in $B_{{\sigma }}^{K}(I;\epsilon _0)$ we define, for $\varrho < {\sigma }- s_0$, the symbol

$$\begin{aligned} (a\#_{\varrho } b)\left( U;t, x,\xi \right) \triangleq \sum _{k=0}^{\varrho }\frac{1}{k!} \left( \frac{\textrm{i}}{2}{\sigma }\left( D_{x},D_{\xi },D_{y},D_{\eta } \right) \right) ^{k} \Big [a(U;t, x,\xi )b(U;t,y,\eta )\Big ]_{|_{\begin{array}{c} x=y, \xi =\eta \end{array}}} \end{aligned}$$

modulo symbols in $\Sigma \Gamma ^{m+m'-\varrho }_{K,K',p+p'}[\epsilon _0,N]$.

The symbol $a\#_{\varrho } b$ belongs to $\Sigma \Gamma ^{m+m'}_{K,K',p+p'}[\epsilon _0,N]$. Moreover,

$$\begin{aligned} a\#_{\varrho }b = a b + \frac{1}{2 \textrm{i}}\{a,b\}, \end{aligned}$$

up to a symbol in $\Sigma \Gamma ^{m+m'-2}_{K,K',p+p'}[\epsilon _0,N]$, where

$$\begin{aligned} \{a,b\} \triangleq \partial _{\xi }a \ \partial _{x}b -\partial _{x}a \ \partial _{\xi }b \end{aligned}$$

denotes the Poisson bracket.

The following result is proven in Proposition 3.12 in [61]:

#### Proposition 3.15

(Composition of Bony-Weyl operators) Let $p,q,N, K, K' \in \mathbb {N}$ with $K' \leqslant K$, $\varrho {\geqslant }0$, $m,m'\in \mathbb {R}$, $\epsilon _0>0$. Consider symbols $a\in \Sigma {\Gamma }^{m}_{K,K',p}[\epsilon _0,N]$ and $b\in \Sigma {\Gamma }^{m'}_{K,K',q}[\epsilon _0, N]$. Then

$$\begin{aligned} {{{\,\mathrm{Op^{BW}}\,}}\left( a(U;t, x,\xi )\right) }\circ {{{\,\mathrm{Op^{BW}}\,}}\left( b(U;t, x,\xi )\right) } - {{{\,\mathrm{Op^{BW}}\,}}\left( (a\#_{\varrho } b)(U;t, x,\xi )\right) } \end{aligned}$$

is a smoothing operator in $\Sigma {\mathcal {R}}^{-\varrho +m+m'}_{K,K',p+q}[\epsilon _0,N]$.

We have the following result, see e.g. Lemma 7.2 in [61]:

#### Lemma 3.16

(Bony paraproduct decomposition) Let $u_1, u_2$ be functions in $H^{\sigma }(\mathbb {T};\mathbb {C})$ with ${\sigma }>\frac{1}{2}$. Then

$$\begin{aligned} u_1 u_2 = {{{\,\mathrm{Op^{BW}}\,}}\left( u_1 \right) }u_2 + {{{\,\mathrm{Op^{BW}}\,}}\left( u_2 \right) }u_1 + R_1(u_1)u_2 + R_2(u_2)u_1 \end{aligned}$$

where for $\textsf{j} =1, 2$, $R_\textsf{j}$ is a homogeneous smoothing operator in $\widetilde{\mathcal {R}}^{-\varrho }_{1}$ for any $\varrho {\geqslant }0$.

We now state other composition results for *m*-operators which follow as in [20, Proposition 2.15].

#### Proposition 3.17

(Compositions of *m*-operators) Let $p, p', N, K, K' \in \mathbb {N}$ with $K'\leqslant K$ and $\epsilon _0>0$. Let $m,m' \in \mathbb {R}$. Then

1. If *M*(*U*; *t*) is in $$ \Sigma \mathcal {M}^{m}_{K,K',p}[\epsilon _0,N]$$ and $$M'(U;t) $$ is in $$ \Sigma \mathcal {M}^{m'}_{K,K',p'}[\epsilon _0,N] $$ then the composition $$ M(u;t)\circ M'(U;t) $$ is in $$\Sigma \mathcal {M}^{m+\max (m',0)}_{K,K',p+p'}[\epsilon _0,N]$$.
2. If *M*(*U*) is a homogeneous *m*-operator in $\widetilde{\mathcal {M}}_{p}^{m}$ and $M^{(\ell )}(U;t)$, $\ell =1,\dots ,p+1$, are matrices of $m_\ell$-operators in $\Sigma \mathcal {M}^{m_\ell }_{K,K',q_\ell }[\epsilon _0,N]$ with $m_\ell \in \mathbb {R}$, $q_\ell \in \mathbb {N}$, then
  $$\begin{aligned} M \left( M^{(1)}(U;t)u, \ldots , M^{(p)}(U;t)U \right) M^{(p+1)}(U;t) \end{aligned}$$
  belongs to $\Sigma \mathcal {M}_{K,K',p+\bar{q}}^{m+ \bar{m}}[\epsilon _0,N]$ with $\bar{m}\triangleq \sum _{\ell =1}^{p+1} \max (m_\ell ,0)$ and $\bar{q}\triangleq \sum _{\ell =1}^{p+1}q_\ell$.
3. Let *a* be a symbol in ${{\Sigma \Gamma }^{m}_{K,K',p}[\epsilon _0,N]}$ with $m{\geqslant }0$ and *R* a smoothing operator in ${{\Sigma {\mathcal {R}}}^{-\varrho }_{K,K',p'}[\epsilon _0 ,N]}$. Then
  $$\begin{aligned} {{{\,\mathrm{Op^{BW}}\,}}\left( a(U; t, \cdot )\right) } \circ R(U;t) \, , \ \ R(U;t) \circ {{{\,\mathrm{Op^{BW}}\,}}\left( a(U; t, \cdot )\right) } \, \ \in {{\Sigma {\mathcal {R}}}^{-\varrho +m}_{K,K',p+p'}[\epsilon _0 ,N]}. \end{aligned}$$
4. If $a_p$ is in $\widetilde{\Gamma }_p^m$ and $M(U)\in \Sigma \mathcal {M}_{K,K',p'}^{m'}[\epsilon _0,N]$ then $a_p(M(U), U, \ldots , U;x,\xi )\in \Sigma \Gamma ^m_{K,K',p+p'}[\epsilon _0,N]$ and
  $$\begin{aligned} {{{\,\mathrm{Op^{BW}}\,}}\left( a(W, U, \ldots , U;x,\xi )\right) }_{|W= M(U)} = {{{\,\mathrm{Op^{BW}}\,}}\left( a_p(M(U), U, \ldots , U;x,\xi )\right) }+ R(U) \end{aligned}$$
  where $R(U) \in \Sigma \mathcal {R}_{K,K',p+p'}^{-\varrho }[\epsilon _0,N]$, for any $\varrho >0$. In particular if $a \in \Sigma \Gamma _{K,K',p}^{m}[\epsilon _0,N]$ then
  $$\begin{aligned}&\text { If} \quad \partial _t U = M_0(U)U, \quad M_0(U)\in \Sigma \mathcal {M}_{K,K',0}^{m'}[\epsilon _0,N],\\&\text {then} \quad \partial _t {{{\,\mathrm{Op^{BW}}\,}}\left( a(U; x, \xi )\right) }= {{{\,\mathrm{Op^{BW}}\,}}\left( a_{[1]}(U;x,\xi )\right) }+ R(U), \end{aligned}$$
  where $$a_{[1]}\in \Sigma \Gamma _{K,K'+1,p}^{m}[\epsilon _0,N]$$ and $$R(U)\in \Sigma \mathcal {R}_{K,K',p'}^{-\varrho }[\epsilon _0,N]$$

#### Notation 3.18

In the sequel if $K'= 0$ we denote a symbol $a (U; t, x, \xi )$ in $\Gamma _{K,0,p}^m[\epsilon _0]$ simply as $a (U; x, \xi )$, and a smoothing operator in *R*(*U*; *t*) in $\Sigma \mathcal {R}^{-\varrho }_{K,0,p}[\epsilon _0,N]$ simply as *R*(*U*) , without writing the *t*-dependence.

We finally provide the Bony paralinearization formula of the composition operator whose proof is a combination of [61, Lemma 3.19].

#### Lemma 3.19

(Bony Paralinearization formula) Let *F* be a smooth $\mathbb {C}$-valued function defined on a neighborhood of zero in $\mathbb {C}$, vanishing at zero at order $q\in \mathbb {N}$. Then there are $s_0,\epsilon _0 > 0$ such that if $u\in B_{H^{s_0}(\mathbb {T};\mathbb {R})}(\varepsilon _0),$ then

$$\begin{aligned} F(u) = \ {{{\,\mathrm{Op^{BW}}\,}}\left( F'\left( u \right) \right) } u + R\left( u \right) u, \end{aligned}$$

where $R\left( u \right)$ is a smoothing operator in   $\Sigma \mathcal {R}^{-\varrho }_{K,0,q'}\left[ \epsilon _0, N \right]$, $q' \triangleq \max (q-1,1)$, for any $\varrho {\geqslant }0$.

### Spectrally Localized Maps

In this section we introduce the class of spectrally localized map, needed to apply the Darboux symplectic correction (see Proposition 7.1). This class was introduced first in [20, Def. 2.15]

#### Definition 3.20

*(Spectrally localized maps)* Let $m \in {\mathbb {R}}, p, N \in {\mathbb {N}}, K, K' \in {\mathbb {N}}$ with $K' \leqslant K$ and $r > 0$.

i) **Spectrally localized** *p***-homogeneous maps.** We denote by $\widetilde{\mathcal {S}}^m_p$ the subspace of *m*-operators *S*(*U*) in $\widetilde{\mathcal {M}}^m_p$ whose coefficients $S_{\mathbf {\jmath }, j,k}^{\mathbf {\sigma }}$ (see (3.14)) satisfying the following spectral condition: there are $\delta> 0, C > 1$ such that
  $$\begin{aligned}S_{\mathbf {\jmath }, j,k}^{\mathbf {\sigma }}\not =0 \quad \implies | \mathbf {\jmath }| \leqslant \delta | j |, \quad C^{-1} |k| \leqslant |j| \leqslant C |k|.\end{aligned}$$
  We denote $\widetilde{\mathcal {S}} \triangleq \bigcup _{p} \widetilde{\mathcal {S}}^m_p$ and by $\Sigma ^N_p \widetilde{\mathcal {S}}^m_q$ the class of pluri-homogeneous spectrally localized maps of the form $\sum _{q=p}^N S_q$ with $S_q \in \widetilde{\mathcal {S}}^m_q$ and $\Sigma _p \widetilde{\mathcal {S}}^m_q \triangleq \bigcup _{N \in {\mathbb {N}}} \Sigma ^N_p \widetilde{\mathcal {S}}^m_q$. For $p \geqslant N + 1$ we mean that the sum is empty.
ii) **Non-homogeneous spectrally localized maps.** We denote $\mathcal {S}^{m}_{K, K', p}[\epsilon _0]$ the space of maps $(U, t, V) \mapsto S(U; t)V$ defined on $B^{K'}_{K}(I; r) \times I \times C^{0}(I, H^{s_0}(T, {\mathbb {C}}))$ for some $s_0 > 0$, which are linear in the variable *V* and such that the following holds true. For any $s \in {\mathbb {R}}$ there are $C > 0$ and $r(s) \in [0, \epsilon _0]$ such that for any $U \in B^{K'}_{K}(I; r(s)) \cap C^{*}(I, H^{s}(T; {\mathbb {C}}^2))$, any $V \in C^{*}_{K - K'}(I, H^{s}(T, {\mathbb {C}})),$ any $0 \leqslant k \leqslant K - K', t \in I$, we have that
  $$\begin{aligned} \Vert \partial ^{k}_{t} (S(U; t)V) (t, \cdot ) \Vert _{H^{s - \frac{3}{2}k - m}} \leqslant&C \sum _{k' + k'' = k} \Vert U \Vert ^{p}_{k', s_0} \Vert V \Vert _{H^{k'', s}},\quad  &   \text {if} \ p\geqslant 1,\\ \Vert \partial ^{k}_{t} (S(U; t)V) \Vert _{H^{s - \frac{3}{2}k - m}} \leqslant&C \Vert V \Vert _{k, s},\quad  &   \text {if} \ p=0. \end{aligned}$$
  We denote that $S^{m}_{K, K', N}[\epsilon _0] \triangleq \bigcup _{p} \mathcal {S}^{m}_{K, K', p}[\epsilon _0]$.
iii) **Spectrally localized Maps.** We denote by $\Sigma \mathcal {S}^m_{K, K', p}[r, N]$ the space of maps $(U, t, V) \mapsto S(U; t)V$ of the form
  $$\begin{aligned} S(U; t)V = \sum _{q = p}^{N} S_q (U)V + S_{> N}(U; t)V, \end{aligned}$$
  where $S_q$ are spectrally localized homogeneous maps in $\widetilde{\mathcal {S}}^m_q, q = p, \ldots , N$ and $S_{> N}$ is a non-homogeneous spectrally localized map in $\mathcal {S}^{m}_{K, K', N + 1}[\epsilon _0]$. We denote by $\left( \Sigma ^m_{K, K', p}[r, N] \right) ^{2\times 2}$ the space of $2 \times 2$ matrices whose entries are spectrally localized maps in $\Sigma ^{m}_{K, K', p}[r, N]$. We will use also the notation $\Sigma ^m_{K, K', p}[r, N] \triangleq \bigcup _{l {\geqslant }0} \Sigma ^m_{K, K', p}[r, N + l]$.

### *z*-Dependent Paradifferential Calculus

In this section, $z \in {\mathbb {T}} \setminus \{0\}$ represents a variable on the torus ${\mathbb {T}} \triangleq {\mathbb {R}}/(2\pi {\mathbb {Z}})$. The notation $\left| z \right| _{{\mathbb {T}}}$ denotes the distance on the torus, i.e., $\left| z \right| _{{\mathbb {T}}} \triangleq \min _{k \in {\mathbb {Z}}} \left| z - 2\pi k \right|$.

The following “Kernel-functions", that depend on the "convolutive $2\pi$-periodic variable" *z*, have to be considered as Taylor remainders of functions $K\left( u; x, z \right)$ at $z=0$ which are smooth in *u* and which have finite regularity in *x* and *z*. A Kernel function is a *z*-dependent family of functions (cfr. Definition 3.3) with coefficients of size proportional to $|z|^n_{{\mathbb {T}}}$. For $n > - 1$ such singularity is integrable in *z*.

#### Definition 3.21

*(Kernel functions)* Let $n\in \mathbb {R}$, $p,N\in \mathbb {N}$, $K \in \mathbb {N}$, and $\epsilon _0>0$.

i) *p*-**homogeneous Kernel-functions.** If $p \in \mathbb {N},$ we denote $\widetilde{K\mathcal {F}}^n_p$ as the space of *z*-dependent, *p*-homogeneous maps from $\dot{H}^\infty \left( {\mathbb {T}};{\mathbb {C}} \right)$ to the space of *x*-translation invariant real functions $\kappa (u;x,z)$ of class $\mathcal {C}^\infty$ in $(x, z ) \in {\mathbb {T}}^2$ with Fourier expansion
  $$\begin{aligned} \kappa (u;x,z)= \sum _{j_1, \ldots , j_p \in \mathbb {Z}^*} \! \kappa _{j_1, \ldots , j_p} (z) u_{j_1} \cdots u_{j_p} e^{\textrm{i}(j_1+ \cdots + j_p) x} \, , \quad z \in \mathbb {T}\setminus \{ 0 \} \, , \end{aligned}$$
  with coefficients $\kappa _{j_1, \ldots , j_p} (z)$ of class $\mathcal {C}^\infty \left( {\mathbb {T}}\setminus \{0\}; \mathbb {C} \right)$, symmetric in $(j_1, \ldots , j_p )$, satisfying the reality condition $\overline{\kappa _{j_1, \ldots , j_p}}\left( z \right) = \kappa _{-j_1, \ldots , -j_p} \left( z \right)$ and the following: for any $l \in \mathbb {N}$, there exist $\mu > 0$ and a constant $C > 0$ such that
  3.20 $$\begin{aligned} \left| \partial _{z}^{l} \kappa _{j_1, \ldots , j_p} (z) \right| \leqslant C \left| \vec {\jmath }_p \right| ^{\mu }\ \left| z \right| ^{n-l}_{{\mathbb {T}}} \, , \quad \forall \mathbf {\jmath }_p = \left( j_1, \ldots , j_p \right) \in (\mathbb {Z}^*)^p \, . \end{aligned}$$
  For $p = 0$ we denote by $\widetilde{K\mathcal {F}}^n _0$ the space of maps $z \mapsto \kappa (z)$ which satisfy $\left| \partial _{z}^{l} \kappa (z) \right| \leqslant C \, \left| z \right| ^{n-l}_{{\mathbb {T}}}$.
ii) **Non-homogeneous Kernel-functions.** We denote by $K\mathcal {F}_{K,0,p}^{n}[\epsilon _0]$ the space of *z*-dependent, real functions $\kappa (u;x,z)$, defined for $u \in B_{s_0}^0 (I;\epsilon _0)$ for some $s_0$ large enough, such that for any $0\leqslant k\leqslant K$ and , any $s\geqslant s_0$, there are $C>0$, $0<\epsilon _0(s)<\epsilon _0$ and for any $u \in B_{s_0}^K\left( I;\epsilon _0(s) \right) \cap C_{*}^{k}\left( I, \dot{H}^{s} \left( {\mathbb {T}};{\mathbb {C}} \right) \right)$ and any ${\texttt{b}}\in \mathbb {N}$, with $\alpha \leqslant s-s_0,$ one has the estimate
  3.21 $$\begin{aligned} \left| \partial _t^k\partial _x^\alpha \partial _z ^l \kappa \left( u;x, z \right) \right| \leqslant C \Vert u \Vert _{k,s_0}^{p-1}\Vert u\Vert _{k,s} \ \left| z \right| ^{n-l}_{{\mathbb {T}}} \, , \qquad z\in {\mathbb {T}}\setminus \{ 0 \} \, . \end{aligned}$$
  If $p = 0$ the right hand side in (3.21) has to be replaced by $\left| z \right| ^{n-l}_{{\mathbb {T}}}$.
iii) **Kernel-functions.** We denote by $\Sigma K\mathcal {F}_{K,0,p}^{n}[\epsilon _0,N]$ the space of real functions of the form
  $$\begin{aligned} \kappa (u;x,z)= \sum _{q=p}^{N} \kappa _q\left( u;x,z \right) + \kappa _{>N}(u;x,z ) , \end{aligned}$$
  where $\kappa _q \left( u;x,z \right)$, $q=p,\dots , N$ are homogeneous Kernel functions in $\widetilde{K\mathcal {F}}_q^n$, and $\kappa _{>N}(u;x,z )$ is a non-homogeneous Kernel function in $K\mathcal {F}_{K,0,N+1}^{n} [\epsilon _0]$. A Kernel function $\kappa (u;x,z)$ is *real* if it is real valued for any $u \in B_{s_0,\mathbb {R}}^0 (I;\epsilon _0)$.

We list some properties of the Kernel functions. In view of the second point of Remark 3.2, a homogeneous Kernel function $\kappa ( u; x,z)$ in $\widetilde{K\mathcal {F}}^n_p$ defines a non-homogeneous Kernel function in $K\mathcal {F}_{K,0,p}^{n}[\epsilon _0]$ for any $\epsilon _0 >0$.

#### Remark 3.22

Let us make some remarks.

- Let $\kappa \left( u;x, z \right)$ be a Kernel function in $\Sigma \mathcal {F}^{n}_{K, 0, p}\left[ \epsilon _0, N \right]$ with $n{\geqslant }0$, which admits a continuous extension in $z=0$. Then its trace $\kappa \left( u;x, 0 \right)$ at $z = 0$ is a function in $\Sigma \mathcal {F}^{{\mathbb {R}}}_{K, 0, p}\left[ \epsilon _0, N \right]$.
- If $\kappa (u; x, z)$ is a homogeneous Kernel function $\widetilde{K\mathcal {F}}^n_p$, the two definitions of quantization in (3.9) differ by a Kernel smoothing operator in $\widetilde{K\mathcal {R}}^{-\varrho ,n}_p$, for any $\varrho > 0$, according to Definition 3.25 below.
- (Sum and product of Kernel functions) If $\kappa _1 (u;x,z)$ is a Kernel function in $\Sigma K\mathcal {F}^{n_1}_{K, 0, p_1}\left[ \epsilon _0, N \right]$ and $\kappa _2 (u;x,z)$ in $\Sigma K\mathcal {F}^{n_2}_{K, 0, p_2}\left[ \epsilon _0, N \right]$, then the sum $(\kappa _1 + \kappa _2 )(u;x,z)$ is a Kernel function in $\Sigma K\mathcal {F}^{\min \left\{ n_1, n_2 \right\} }_{K, 0, \min \left\{ p_1, p_2 \right\} }\left[ \epsilon _0, N \right]$ and the product $( \kappa _1 \kappa _2 )(u;x,z)$ is a Kernel function in $\Sigma K\mathcal {F}^{n_1+ n_2}_{K, 0, p_1 + p_2}\left[ \epsilon _0, N \right]$.
- (Integral of Kernel functions) Let $\kappa \left( u; x, z \right)$ be a Kernel function in $\Sigma K\mathcal {F}^{n}_{K, 0, p}\left[ \epsilon _0, N \right]$ with $n>-1$. Then $\mathop {\,{--}\!\!\!\int }\nolimits \kappa \left( u; x, z \right) \text {d}z$ is a function in $\Sigma \mathcal {F}^{{\mathbb {R}}}_{K, 0, p}\left[ \epsilon _0, N \right]$. This follows directly integrating (3.20) and (3.21) in *z*.

The *m*-Kernel-operators defined below are a *z*-dependent family of *m*-operators (cfr. Definition 3.8) with coefficients of size proportional to $$ |z|^n_{{\mathbb {T}}} $$. For $$ n > - 1 $$ such singularity is integrable in *z*. A family of *z*-dependent paraproduct operators associated to Kernel functions defines a 0-Kernel operator, see Remark 3.24. The kind of operators will appear only in case $$ m < 0$$, as smoothing operators in the composition of Bony-Weyl quantizations of Kernel-functions (see Definition 3.25).

#### Definition 3.23

Let $$ m,n \in \mathbb {R}$$, $$p,N\in \mathbb {N}$$, $$K\in \mathbb {N}$$ with $$ \epsilon _0 > 0 $$.

i) *p***-homogeneous** *m***-Kernel-operator.** We denote by $\widetilde{K{\mathcal {M}}}^{m,n}_{p}$ the space of *z*-dependent, *x*-translation invariant homogeneous *m*-operators according to Definition 3.8, Item 3.8, in which the constant *C* is substituted with $C\left| z \right| ^n_{{\mathbb {T}}}$, equivalently,
  3.22 $$\begin{aligned} M( u ;z)v \left( x \right) = \sum _{\begin{array}{c} (\mathbf {\jmath }_{p},j,k) \in \mathbb {Z}^{p+2} \\ j_1 + \ldots + j_p + j = k \end{array} } M_{\mathbf {\jmath }_{p}, j,k}\left( z \right) u_{j_1} \ldots u_{j_p} v_{j} e^{\textrm{i}k x} \, , \qquad z \in {\mathbb {T}}\setminus \{ 0 \} \, , \end{aligned}$$
  with coefficients satisfying
  3.23 $$\begin{aligned} \big | M_{\mathbf {\jmath }_p, j, k}\left( z \right) \big | \leqslant C \ \textrm{max} _2 \left\{ \left| j_1 \right| , \ldots , \left| j_{p} \right| , \left| j \right| \right\} ^\mu \ \max \left\{ \left| j_1 \right| , \ldots , \left| j_{p} \right| , \left| j \right| \right\} ^m \left| z \right| ^n_{{\mathbb {T}}} \, .\nonumber \\ \end{aligned}$$
  If $p=0$ the right hand side of (3.22) is replaced by $\sum _{j\in \textbf{Z}} M_j \left( z \right) v_j e^{\textrm{i}j x}$ with $\big | M_j \left( z \right) \big | \leqslant C \left| j \right| ^m \left| z \right| ^n_{{\mathbb {T}}}$.
ii) **Non-homogeneous** *m***-Kernel-operator.** We denote by $K{\mathcal {M}}^{m,n}_{K,0,p}[\epsilon _0]$ the space of *z*-dependent, non-homogeneous operators *M*(*u*; *z*)*v* defined for any $z \in {\mathbb {T}}\setminus \{ 0 \}$, such that for any $0\leqslant k\leqslant K$
  3.24 $$\begin{aligned}  &   \left\| {\partial _t^k\left( M(u;z)v\right) } \right\| _{s- \frac{3}{2} k-m} \nonumber \\  &   \quad \leqslant C \left| z \right| ^n_{{\mathbb {T}}} \ \sum _{k'+k''=k} \left( \Vert {v}\Vert _{k'',s}\Vert {u}\Vert _{k',{s_0}}^{p} +\Vert {v}\Vert _{k'', {s_0}}\Vert u\Vert _{k', {s_0}}^{p-1}\Vert u \Vert _{k', s} \right) \, . \end{aligned}$$
iii) *m***-Kernel-Operator.** We denote by $\Sigma K{\mathcal {M}}^{m,n}_{K,0,p}[\epsilon _0,N]$ the space of operators of the form
  3.25 $$\begin{aligned} M(u;z)v =\sum _{q=p}^{N}M_{q}(u)v+M_{>N}(u;z)v \end{aligned}$$
  where $$M_{q} $$ are homogeneous *m*-Kernel operators in $$ \widetilde{K{\mathcal {M}}}^{m,n}_{q}$$, $$q=p,\ldots , N$$ and $$M_{>N}$$ is a non-homogeneous *m*-Kernel-operator in $$\mathcal {M}^{m,n}_{K,0,N+1}[\epsilon _0]$$. We denote by $$ \Sigma _p^N \widetilde{\mathcal {M}}^{m}_q $$ the space pluri-homogeneous *m*-Kernel operators of the form (3.25) with $$ M_{>N} = 0 $$.

#### Remark 3.24

Given a Kernel function $\kappa \left( u;x, z \right)$ in $\Sigma K\mathcal {F}^{n}_{K,0,p}\left[ \epsilon _0, N \right]$ then ${{{\,\mathrm{Op^{BW}}\,}}\left( \kappa \left( u;x, z \right) \right) }$ is 0- Kernel operator in $\Sigma K{\mathcal {M}}^{0, n}_{K, 0, p}\left[ \epsilon _0, N \right]$.

#### Definition 3.25

*(Kernel-smoothing operators)* Given $\varrho > 0$ we define the homogeneous and non-homogeneous Kernel-smoothing operators as

$$\begin{aligned} \begin{aligned}&\widetilde{K\mathcal {R}} ^{-\varrho ,n}_p \triangleq \widetilde{K{\mathcal {M}}} ^{-\varrho ,n}_p ,\quad K\mathcal {R}^{-\varrho ,n}_{K, 0, p}\left[ \epsilon _0 \right] \triangleq K{\mathcal {M}}^{-\varrho ,n}_{K, 0, p}\left[ \epsilon _0 \right] , \quad \\  &\Sigma K\mathcal {R}^{-\varrho ,n}_{K, 0, p}\left[ \epsilon _0, N \right] \triangleq \Sigma K{\mathcal {M}}^{-\varrho ,n}_{K, 0, p}\left[ \epsilon _0, N \right] . \end{aligned} \end{aligned}$$

In view of [20, Lemma 2.8], if $M\left( u, \ldots , u;z \right)$ is a homogeneous *m*-Kernel operator in $\widetilde{K{\mathcal {M}}}^{m,n}_p$ then $M\left( u, \ldots , u; z \right)$ defines a non-homogeneous *m*-Kernel operator in $K{\mathcal {M}}^{m,n}_{K, 0, p}\left[ \epsilon _0 \right]$ for any $\epsilon _0 > 0$ and $K\in {\mathbb {N}}$.

The classes of paraproducts associated to Kernel functions and *m*-Kernel-operators are closed w.r.t. compositions as we list below, cf. [21].

#### Proposition 3.26

(Composition of *z*-dependent operators) Let $m,n,m',n'\in {\mathbb {R}}$, and integers $K, p, p', N\in {\mathbb {N}}$ with $p, p'\leqslant N$.

1. Let $\kappa \left( u;x,z \right) \in \Sigma K\mathcal {F}^{n}_{K, 0, p}\left[ \epsilon _0, N \right]$ and $\kappa ' \left( u;x,z \right) \in \Sigma K\mathcal {F}^{n'}_{K, 0, p'}\left[ \epsilon _0, N \right]$ be Kernel functions. Then
  $$\begin{aligned} {{{\,\mathrm{Op^{BW}}\,}}\left( \kappa \left( u;x,z \right) \right) }\circ {{{\,\mathrm{Op^{BW}}\,}}\left( \kappa '\left( u;x,z \right) \right) } = {{{\,\mathrm{Op^{BW}}\,}}\left( \kappa \ \kappa ' \left( u;x,z \right) \right) } + R\left( u;z \right) , \end{aligned}$$
  where $R\left( u;z \right)$ is a Kernel-smoothing operator in $\Sigma K\mathcal {R}^{-\varrho , n+n'}_{K, 0, p+p'}\left[ \epsilon _0, N \right]$ for any $\varrho {\geqslant }0$;
2. Let $$ M \left( u; z \right) $$ be a *m*-Kernel operator in $$ \Sigma K{\mathcal {M}}^{m, n}_{K, 0,p}\left[ \epsilon _0, N \right] $$ and $$ M' \left( u; z \right) $$ be an $$ m'$$-operator belonging to $$ \Sigma K{\mathcal {M}}^{m', n'}_{K, 0,p'}\left[ \epsilon _0, N \right] $$. Then $$ M\left( u; z \right) \circ M' \left( u; z \right) $$ belongs to $$ \Sigma K{\mathcal {M}}^{m+\max \left( m',0 \right) , n+n'}_{K, 0, p+p'}\left[ \epsilon _0, N \right] $$;
3. Let $\kappa \left( u;x,z \right)$ be a Kernel function in $\Sigma K\mathcal {F}^n_{K, 0, p}\left[ \epsilon _0, N \right]$ and $R\left( u;z \right)$ be a Kernel smoothing operator in $\Sigma K\mathcal {R}^{-\varrho , n'}_{K, 0, p'}\left[ \epsilon _0, N \right]$ then ${{{\,\mathrm{Op^{BW}}\,}}\left( \kappa \left( u; x, z \right) \right) }\circ R\left( u;z \right)$ and $R\left( u;z \right) \circ {{{\,\mathrm{Op^{BW}}\,}}\left( \kappa \left( u; x, z \right) \right) }$ are a Kernel smoothing operator in $\Sigma K\mathcal {R}^{-\varrho , n+n'}_{K, 0, p+p'}\left[ \epsilon _0, N \right]$;
4. Let $M \left( u;z \right)$ be an homogeneous *m*-Kernel operator in $\widetilde{K{\mathcal {M}}}^{m,n}_1$, and $M' \left( u;z \right)$ in $\Sigma K{\mathcal {M}}^{0,0}_{K,0,0}\left[ \epsilon _0, N \right]$ then $M\left( M'\left( u;z \right) u;z \right) \in \Sigma K{\mathcal {M}}^{m,n}_{K,0,1}\left[ \epsilon _0, N \right]$.

Finally integrating (3.23) and (3.24) in *z* we deduce the following lemma.

#### Lemma 3.27

*(Integrals of Kernel smoothing operators)* Let $R\left( u;z \right)$ be a Kernel smoothing operator belonging to $\Sigma \mathcal {R}^{-\varrho , n}_{K,0, p}\left[ \epsilon _0, N \right]$ with $n > - 1$. Then

$$\begin{aligned} \int _{{\mathbb {T}}} R\left( u;z \right) g\left( x-z \right) \text {d}z = R_1\left( u \right) g , \qquad \int _{{\mathbb {T}}} R\left( u;z \right) \text {d}z = R_2\left( u \right) , \end{aligned}$$

where $R_1 \left( u \right)$, $R_2 \left( u \right)$ are smoothing operators in $\Sigma \mathcal {R}^{-\varrho }_{K,0,p}\left[ \epsilon _0, N \right]$.

The following proposition will be crucial in Section 4, and is proved in [21]:

#### Proposition 3.28

Let $n > -1$ and $\kappa \left( u; x, z \right)$ be a Kernel-function in $\Sigma K\mathcal {F}^{n}_{K,0,p}\left[ \epsilon _0, N \right]$. Let us define the operator, for any $g \in \dot{H}^s ({\mathbb {T}};{\mathbb {R}})$, $s \in {\mathbb {R}}$,

$$\begin{aligned} \left( \mathcal {T}_\kappa g \right) \left( x \right) \triangleq \int _{{\mathbb {T}}} {{{\,\mathrm{Op^{BW}}\,}}\left( \kappa \left( u; \bullet , z \right) \right) } g\left( x-z \right) \text {d}z \, . \end{aligned}$$

Then there exists

- a symbol $a \left( u; x, \xi \right)$ in $\Sigma \Gamma ^{-\left( 1+n \right) }_{K,0, p}\left[ \epsilon _0, N \right]$ satisfying (3.12);
- a pluri-homogeneous smoothing operator $R\left( u \right)$ in $\Sigma _p^N \widetilde{\mathcal {R}}^{-\varrho }_q$ for any $\varrho > 0$;

such that $$ \mathcal {T}_\kappa g = {{{\,\mathrm{Op^{BW}}\,}}\left( a \left( u; x, \xi \right) \right) } g + R\left( u \right) g $$.

## Paralinearization of the Kelvin-Helmholtz System

### Notation 4.1

In the present section we use the notation

$$\begin{aligned} B^K_{s, \mathbb {R}}\left( I;r \right) \triangleq B_{C^K_*\left( I;\dot{H} ^s\left( \mathbb {T};\mathbb {R} \right) \right) }\left( 0, r \right) . \end{aligned}$$

We warn the reader that such notation is conflictive with the notation introduced at page 19, but we think that in the restricted context of the paralinearization procedure outlined here there is no risk of confusion and it helps to streamline the mathematical statements that we present.

In the present section we paralinearize the system Eq. (1.9) in the $(\eta ,\psi )$-variables. The result we obtain is

### Theorem 4.2

Let $N\in {\mathbb {N}}$, $\gamma \geqslant 0$, ${\texttt{b}}\in {\mathbb {R}}$ and $\varrho \geqslant 0$, for any $K\in {\mathbb {N}}$ there exist $s_0 >0$ and $\epsilon _0 > 0$ such that if $\eta , \psi \in B^K_{s_0, {\mathbb {R}}}\left( I;\epsilon _0 \right)$ is a solution of Eq. (1.9), then $\left( \eta , \psi \right)$ solves the paradifferential equation

4.1 $$\begin{aligned} \begin{bmatrix} \eta _t \\ \psi _t \end{bmatrix}&={{{\,\mathrm{Op^{BW}}\,}}\left( {\boldsymbol{Q}}_{\gamma , {\texttt{b}}}\left( \eta ,\psi ; x, \xi \right) {+} {\boldsymbol{B}}_{\texttt{b}}\left( \eta ,\psi ; x \right) \left| \xi \right| {-} \textrm{i}V_{\texttt{b}}\left( \eta , \psi ;x \right) \text {Id}_{\mathbb {R}^2}\ \xi {+} {\boldsymbol{A}}_{\left[ 0 \right] } \left( \eta ,\psi ; x, \xi \right) \right) } \begin{bmatrix} \eta \\ \psi \end{bmatrix} \nonumber \\&+ {\boldsymbol{R}}\left( \eta , \psi \right) \begin{bmatrix} \eta \\ \psi \end{bmatrix} , \end{aligned}$$

where

- The matrix of symbols ${\boldsymbol{Q}}_{\gamma , {\texttt{b}}}$, whose entries satisfy the reality condition (3.12), is explicitly given by
  4.2 $$\begin{aligned} {\boldsymbol{Q}}_{\gamma , {\texttt{b}}}\left( \eta ,\psi ; x, \xi \right) \triangleq \,&\left[ \begin{array}{cc} 0 &  -\frac{\left| \xi \right| }{2} \\ \gamma \left( 1+\texttt{f}\left( \eta ;x \right) \right) \left( \left| \xi \right| ^2-1 \right) -\left( \frac{{\texttt{b}}^2}{2} + w_{\texttt{b}}\left( \eta , \psi ;x \right) \right) \left| \xi \right| + \frac{{\texttt{b}}^2}{\left( 1+2\eta \right) } &  0 \end{array} \right] \nonumber \\&\in \left( \Sigma \Gamma ^2_{K,0,0}\left[ \epsilon _0, N \right] \right) ^{2\times 2}, \end{aligned}$$
  with
  4.3 $$\begin{aligned} \begin{aligned} \texttt{f}\left( \eta ;x \right)&\triangleq \left( \frac{1+2\eta }{\left( 1+2\eta \right) ^{2}+\eta _x^2} \right) ^{\frac{3}{2}}-1\in \Sigma \mathcal {F}^{{\mathbb {R}}}_{K,0,1}\left[ \epsilon _0, N \right] ,\\ w_{\texttt{b}}\left( \eta , \psi ;x \right)&\triangleq \frac{1}{2}\left( W_{\texttt{b}}^2\left( \eta , \psi ;x \right) -{\texttt{b}}^2 \right) \in \Sigma \mathcal {F}^{{\mathbb {R}}}_{K,0,1}\left[ \epsilon _0, N \right] ,\\ W_{\texttt{b}}\left( \eta , \psi ;x \right)&\triangleq \left( \psi _x+{\texttt{b}} \right) \frac{1+2\eta }{\left( 1+2\eta \right) ^2+\eta _x^2}\in \Sigma \mathcal {F}^{{\mathbb {R}}}_{K,0,0}\left[ \epsilon _0, N \right] . \end{aligned} \end{aligned}$$
  In particular, $$ W_{\texttt{b}}\left( 0, 0;x \right) \equiv {\texttt{b}}$$;
- 4.4 $$\begin{aligned} {\boldsymbol{B}}_{\texttt{b}}\left( \eta ,\psi ; x \right) \triangleq \frac{1}{2} \left[ \begin{array}{cc} B_{\texttt{b}}\left( \eta , \psi ; x \right) &  0 \\ B_{\texttt{b}}^2\left( \eta , \psi ; x \right) &  -B_{\texttt{b}}\left( \eta , \psi ; x \right) \end{array} \right] \in \left( \Sigma \mathcal {F}^{{\mathbb {R}}}_{K,0,1}\left[ \epsilon _0, N \right] \right) ^{2\times 2} , \end{aligned}$$
  where
  $$\begin{aligned}  &   B_{\texttt{b}}\left( \eta , \psi ;x \right) \triangleq \left( \psi _x+{\texttt{b}} \right) \frac{\textsf{J}^0\left( \eta ;x \right) }{1+2\eta }\in \Sigma \mathcal {F}^{{\mathbb {R}}}_{K,0,1}\left[ \epsilon _0, N \right] ,\qquad \\  &   \textsf{J}^0\left( \eta ;x \right) \triangleq \frac{2\eta _x\left( 1+2\eta \right) }{\left( 1+2\eta \right) ^2+\eta _x^2}\in \Sigma \mathcal {F}^{{\mathbb {R}}}_{K, 0, 1}\left[ \epsilon _0, N \right] .\end{aligned}$$
  In particular, each entry of ${\boldsymbol{B}}_{{\texttt{b}}}$ satisfies the reality condition (3.12);
- $V_{\texttt{b}}\left( \eta , \psi ;x \right) \in \Sigma \mathcal {F}^{{\mathbb {R}}}_{K, 0, 1}\left[ \epsilon _0, N \right]$ and is explicitly defined as 4.5 where  is defined in (1.5);
- the matrix of symbols ${\boldsymbol{A}}_{\left[ 0 \right] }\left( \eta ,\psi ; x, \xi \right)$, whose entries satisfy the reality condition (3.12), belongs to $\left( \Sigma \Gamma ^0_{K,0,1}\left[ \epsilon _0, N \right] \right) ^{2\times 2}$ and is given by
  $$\begin{aligned} {\boldsymbol{A}}_{\left[ 0 \right] } \left( \eta ,\psi ; x, \xi \right) \triangleq \left[ \begin{array}{cc} A_{\left[ 0 \right] }^\eta \left( \eta ,\psi ; x, \xi \right) &  A_{\left[ -1 \right] }^\eta \left( \eta ,\psi ; x, \xi \right) \\ A_{\left[ 0 \right] }^\psi \left( \eta ,\psi ; x, \xi \right) &  A_{\left[ -1 \right] }^\psi \left( \eta ,\psi ; x, \xi \right) \end{array} \right] , \end{aligned}$$
  with $A_{\left[ m \right] }^\textsf{u} \left( \eta ,\psi ; x, \xi \right) \in \Sigma \Gamma ^m _{K,0,1}\left[ \epsilon _0, N \right]$ for $\textsf{u}=\eta , \psi$ and $m\in {\mathbb {R}}$;
- ${\boldsymbol{R}}\left( \eta , \psi \right) \in \left( \Sigma \mathcal {R}^{-\varrho }_{K,0,1}\left[ \epsilon _0, N \right] \right) ^{2\times 2}$ and is real-valued.

### Remark 4.3

Notice that the quasilinear contribution $-\left( \frac{{\texttt{b}}^2}{2} + w_{\texttt{b}}\left( \eta , \psi ;x \right) \right) \left| \xi \right| =\frac{W_{\texttt{b}}^2 \left( \eta , \psi ;x \right) }{2} \ \left| \xi \right|$ is not nil when ${\texttt{b}}=0$, as it is evident from Eqs. (4.68) and (4.69). This term, in particular, is an unstable contribution that is not present in the one-phase version of the present system, cf. [20, 61, 69].

### Notation 4.4

Along this section, for $\eta \in B^{K}_{s_0, \mathbb {R}}\left( I;\epsilon _0 \right)$ and $$ W\triangleq \begin{bmatrix} w_1 \\ w_2 \end{bmatrix} \in \left( B^{K}_{s_0, \mathbb {R}}\left( I;\epsilon _0 \right) \right) ^2 $$, we use the notations

1. For any $x, z\in {\mathbb {T}}$ (cf. (1.3))
  4.6 $$\begin{aligned} \begin{aligned} r=r\left( x \right) = r\left( \eta ;x \right) \triangleq&\ \sqrt{1+2\eta \left( x \right) } \in \Sigma \mathcal {F}^{{\mathbb {R}}}_{K, 0, 1}\left[ \epsilon _0, N \right] , \\ \delta _z\eta \triangleq&\ \eta \left( x \right) -\eta \left( x-z \right) \in \widetilde{K\mathcal {F}}_1^{\ 1}, \\ \Delta _z \eta \triangleq&\ \frac{\delta _z \eta }{2\sin (z/2)} = \frac{\eta (x) - \eta (x-z)}{2\sin (z/2)}\cdot \end{aligned} \end{aligned}$$
2. $V\left( W;x \right)$ is a generic element in $\Sigma \mathcal {F}^{{\mathbb {R}}} _{K, 0, 1}\left[ \epsilon _0, N \right]$ (cf. Definition 3.3) and $V^n\left( W;x, z \right)$ is a generic element in $\Sigma K\mathcal {F}^n _{K, 0, 1}\left[ \epsilon _0, N \right]$, $n > -1$ (cf. Definition 3.21);
3. $A_{\left[ m \right] } \left( W;x, \xi \right)$ is a generic element in $\Sigma \Gamma ^m _{K, 0, 1}\left[ \epsilon _0, N \right]$ for $m\in {\mathbb {R}}$ that satisfies (3.12) and ${\boldsymbol{A}}_{\left[ m \right] } \left( W;x, \xi \right)$ is a generic element in $\left( \Sigma \Gamma ^m _{K, 0, 1}\left[ \epsilon _0, N \right] \right) ^{2\times 2}$ for $m\in {\mathbb {R}}$ whose entries satisfy (3.12);
4. $R\left( W \right)$ is a generic element in $\Sigma \mathcal {R}^{-\varrho } _{K, 0, 1}\left[ \epsilon _0, N \right]$ which is real-valued and $R\left( W; z \right)$ is a generic element in $\Sigma \mathcal {R}^{-\varrho , n} _{K, 0, 1}\left[ \epsilon _0, N \right]$, $n > -1$ which is real-valued. Similarly ${\boldsymbol{R}}\left( W \right)$ is a generic element in $\left( \Sigma \mathcal {R}^{-\varrho } _{K, 0, 1}\left[ \epsilon _0, N \right] \right) ^{2\times 2}$ which is real-valued and ${\boldsymbol{R}}\left( W; z \right)$ is a generic element in $\Sigma \mathcal {R}^{-\varrho , n} _{K, 0, 1}\left[ \epsilon _0, N \right]$, $n > -1$ which is real-valued.

### Remark 4.5

Accordingly to the notation introduced in Notation 4.4 we write that

$$\begin{aligned} {\boldsymbol{R}}\left( W \right) W\cdot \begin{bmatrix} 1 \\ 1 \end{bmatrix} = R_1\left( W \right) w_1 + R_2 \left( W \right) w_2, \end{aligned}$$

where $R_1$ and $R_2$ are elements of the space $\Sigma \mathcal {R}^{-\varrho }_{K, 0, 1}\left[ \epsilon _0, N \right]$. The same holds when we write $$ {\boldsymbol{R}}\left( W;z \right) W\cdot \begin{bmatrix} 1 \\ 1 \end{bmatrix} $$.

Notice that from Eq. (1.5) we derive the relation4.7where4.8

### Paralinearization of 

The present section is dedicated to the paralinearization of the nonlocal operator  given in (4.8). The result reads as follow:

#### Proposition 4.6

Let $N\in {\mathbb {N}}$ and $\varrho \geqslant 0$, for any $K\in {\mathbb {N}}$ there exist $s_0 > 0$ and $\epsilon _0 > 0$ such that if $\eta , g \in B^{K}_{s_0, \mathbb {R}}\left( I;\epsilon _0 \right)$, the nonlinear operator  in (4.8) admits the paralinearization

1. 4.9 where
  4.10 $$\begin{aligned} \begin{aligned} \textsf{K}^0\left( \eta ;x \right)&\triangleq -\frac{2\eta _x^2}{\left( 1+2\eta \right) ^2+\eta _x^2}\in \mathcal {F}^{{\mathbb {R}}}_{K, 0, 1}\left[ \epsilon _0, N \right] ,\\ \textsf{K}^{\prime 0}\left( \eta ;x \right)&\triangleq -\frac{4\eta _x\left( 1+2\eta \right) ^3}{\left( \left( 1+2\eta \right) ^2+\eta _x^2 \right) ^2}\in \mathcal {F}^{{\mathbb {R}}}_{K, 0, 1}\left[ \epsilon _0, N \right] , \end{aligned} \end{aligned}$$
  $A_{\left[ m \right] }\left( \eta , g;x, \xi \right) \in \Sigma \Gamma ^{m} _{K, 0, 1}\left[ \epsilon _0, N \right]$ satisfies (3.12) and ${\boldsymbol{R}}\left( \eta , g \right) \in \left( \Sigma \mathcal {R}^{-\varrho } _{K, 0, 1}\left[ \epsilon _0, N \right] \right) ^{2\times 2}$ is real-valued.
2. 4.11 where
  $\textsf{K}^{\prime 0}\left( \eta ; x \right) \in \mathcal {F}^{{\mathbb {R}}}_{K, 0, 1}\left[ \epsilon _0, N \right]$ and is explicitly defined in (4.10);
  $$ A_{\left[ m \right] }\left( \eta ;x, \xi \right) \in \Sigma \Gamma ^{m} _{K, 0, 1}\left[ \epsilon _0, N \right] $$ satisfying (3.12);
  $R\left( \eta \right) \in \Sigma \mathcal {R}^{-\varrho } _{K, 0, 1}\left[ \epsilon _0, N \right]$ and is real-valued.

#### Proof of Item 1.

With the notation introduced in (4.6) we can rewrite (4.8) as4.12where

$$\begin{aligned} \textsf{G}_z\left( \textsf{X} \right) \triangleq \frac{\sqrt{1-2\textsf{X}}\sin z}{{1-\textsf{X}-\sqrt{1-2\textsf{X}}\cos z}}\cdot \end{aligned}$$

To handle the singularity of the kernel $g(x-z)/\sin (z/2)$ as $z\rightarrow 0$ (since *g* has zero average), we introduce a desingularized function ${\textsf{K}}_z(X)$ of $\textsf{G}_z$ as

4.13 $$\begin{aligned} \textsf{K}_z \left( \textsf{X} \right) \triangleq \textsf{G}_z\left( \textsf{X}\ 2\sin \left( z/2 \right) \right) \ 2\tan \left( z/2 \right) = \frac{\sqrt{1-4\textsf{X}\ \sin \left( z/2 \right) }\left( 2\sin \left( z/2 \right) \right) ^2}{{1-2\textsf{X}\ \sin \left( z/2 \right) -\sqrt{1-4\textsf{X}\ \sin \left( z/2 \right) }\cos z}} , \end{aligned}$$

and the periodic difference quotient $\Delta _z\eta$ defined in (4.6). Note that ${\textsf{K}}_z(0)=2$, which implies that

$$\begin{aligned}{\textsf{G}}_z(0) = 2 / (2\tan (z/2))=\cot (z/2).\end{aligned}$$

By substituting $X = \frac{\Delta _z \eta }{r^2}$ into the definition (4.13), we get that

$$\begin{aligned}\textsf{K}_z \left( \frac{\Delta _z\eta }{r^2} \right) = \textsf{G}_z\left( \frac{\Delta _z\eta }{r^2} 2\sin (z/2) \right) 2\tan (z/2).\end{aligned}$$

Using (4.6), this becomes $\textsf{K}_z \left( \frac{\Delta _z\eta }{r^2} \right) = \textsf{G}_z\left( \frac{\delta _z\eta }{r^2} \right) 2\tan (z/2).$ Rearranging and subtracting ${\textsf{G}}_z(0)$, we find that

4.14 $$\begin{aligned} \left( \textsf{G}_z \left( \frac{\delta _z\eta }{r^2} \right) - \textsf{G}_z\left( 0 \right) \right) g \left( x-z \right) = \left( \textsf{K}_z \left( \frac{\Delta _z\eta }{r^2} \right) - 2 \right) \frac{g \left( x-z \right) }{2\tan \left( z/2 \right) }\cdot \end{aligned}$$

Notice that from (4.13) we derive that

4.15 $$\begin{aligned} \textsf{K}'_z\left( \textsf{X} \right) =&- \frac{\textsf{X}\left( 2\sin \left( z/2 \right) \right) ^4}{{\left( 1 - 2 \, \textsf{X}\sin \left( z/2\right) - \sqrt{1- 4 \, \textsf{X}\sin \left( z/2\right) } \cos \left( z\right) \right) ^2} \sqrt{1- 4 \, \textsf{X}\sin \left( z/2\right) }} \cdot \end{aligned}$$

We need the following technical result whose proof is postponed at page 35:

#### Lemma 4.7

Let $\textsf{K}_z (\textsf{X})$, be as in Eq. (4.13). Then

4.16 $$\begin{aligned} \textsf{K}_z \left( \frac{\Delta _z \eta }{1+2\eta } \right) - 2 \in \Sigma K\mathcal {F}^{0}_{K, 0, 1}\left[ \epsilon _0, N \right] ,  &   \textsf{K}_z' \left( \frac{\Delta _z \eta }{1+2\eta } \right) \in \Sigma K\mathcal {F}^{0}_{K, 0, 1}\left[ \epsilon _0, N \right] , \end{aligned}$$

are Kernel functions, which admit the expansions

4.17 $$\begin{aligned} \begin{aligned} \textsf{K}_z \left( \frac{\Delta _z \eta }{1+2\eta } \right) - 2 =&\ \textsf{K}^0 \left( \eta ;x \right) + \textsf{K}^1 \left( \eta ;x \right) \ 2\tan \left( z/2 \right) + V^2 \left( \eta ;x,z \right) , \\ \textsf{K}'_z \left( \frac{\Delta _z \eta }{1+2\eta } \right) =&\ \textsf{K}^{\prime 0} \left( \eta ;x \right) + \textsf{K}^{\prime 1} \left( \eta ;x \right) \ \sin z + V^2\left( \eta ;x,z \right) , \end{aligned} \end{aligned}$$

where $\textsf{K}^0\left( \eta ;x \right)$ and $\textsf{K}^{\prime 0}\left( \eta ;x \right)$ are functions in $\mathcal {F}^{{\mathbb {R}}}_{K, 0, 1}\left[ \epsilon _0, N \right]$ having the expressions (4.10).Then, $\textsf{K}^1 \left( \eta ;x \right)$ and $\textsf{K}^{\prime 1} \left( \eta ;x \right)$ are functions in $\Sigma \mathcal {F}^{{\mathbb {R}}}_{K, 0, 1}\left[ \epsilon _0, N \right]$ while $V^2\left( \eta ;x,z \right)$ are Kernel-functions in $\Sigma K\mathcal {F}^{2}_{K, 0, 1}\left[ \epsilon _0, N \right]$ as per Notation 4.4.

Bony paraproduct decomposition (cf. Lemma 3.16) give us that

4.18 $$\begin{aligned} \begin{aligned} \left( \textsf{K}_z \left( \frac{\Delta _z\eta }{r^2} \right) - 2 \right) g\left( x-z \right)&= {{{\,\mathrm{Op^{BW}}\,}}\left( \textsf{K}_z \left( \frac{\Delta _z\eta }{r^2} \right) - 2\right) } g\left( x-z \right) \\&\quad + {{{\,\mathrm{Op^{BW}}\,}}\left( g\left( x-z \right) \right) } \left[ \textsf{K}_z \left( \frac{\Delta _z\eta }{r^2} \right) - 2 \right] \\&\quad + R_1\left( \textsf{K}_z \left( \frac{\Delta _z\eta }{r^2} \right) - 2 \right) g\left( x-z \right) \\&\quad +R_2\left( g\left( x-z \right) \right) \left[ \textsf{K}_z \left( \frac{\Delta _z\eta }{r^2} \right) - 2 \right] . \end{aligned} \end{aligned}$$

In view of (4.16) we can apply Proposition 3.26, Items 4 and 2 and obtain that

4.19 $$\begin{aligned} \begin{aligned}&R_1\left( \textsf {K}_z \left( \frac{\Delta _z\eta }{r^2} \right) - 2 \right) g\left( x-z \right) = R\left( \eta ;z \right) g, \\  &R_2\left( g\left( x-z \right) \right) \left[ \textsf {K}_z \left( \frac{\Delta _z\eta }{r^2} \right) - 2 \right] = R\left( \eta ;z \right) g, \end{aligned} \end{aligned}$$

for suitable $R\left( \bullet ;z \right) \in \Sigma K\mathcal {R}^{-\varrho , 0}_{K, 0, 1}\left[ \epsilon _0, N \right]$. We use now Bony paralinearization formula of Lemma 3.19 and Bony paraproduct decomposition and obtain that

4.20 $$\begin{aligned} \begin{aligned}&\textsf{K}_z \left( \frac{\Delta _z\eta }{r^2} \right) -2\\&= {{{\,\mathrm{Op^{BW}}\,}}\left( \textsf{K}_z' \left( \frac{\Delta _z\eta }{r^2} \right) \right) } \left[ {{{\,\mathrm{Op^{BW}}\,}}\left( r^{-2}\right) }\Delta _z\eta + {{{\,\mathrm{Op^{BW}}\,}}\left( \Delta _z \eta \right) }\left[ r^{-2}-1 \right] +R_1\left( r^{-2}-1 \right) \Delta _z\eta + R_2 \left( \Delta _z \eta \right) \left[ r^{-2}-1 \right] \right] \\&\quad +R\left( \frac{\Delta _z\eta }{r^2} \right) \frac{\Delta _z\eta }{r^2}\cdot \end{aligned} \end{aligned}$$

Then, we apply again Lemma 3.19, Proposition 3.26, Items 4 and 2 in order to get that

4.21 $$\begin{aligned} {{{\,\mathrm{Op^{BW}}\,}}\left( \textsf{K}_z' \left( \frac{\Delta _z\eta }{r^2} \right) \right) } \left( R_1\left( r^{-2}-1 \right) \Delta _z\eta + R_2 \left( \Delta _z \eta \right) \left[ r^{-2}-1 \right] \right) = R\left( \eta ; z \right) \eta ,\end{aligned}$$

4.22 $$\begin{aligned} R\left( \frac{\Delta _z\eta }{r^2} \right) \frac{\Delta _z\eta }{r^2}=R\left( \eta ; z \right) \eta . \end{aligned}$$

Besides, combining Proposition 3.26, Item 1 and Lemma 3.19, we obtain that

4.23 $$\begin{aligned} \begin{aligned}&{{{\,\mathrm{Op^{BW}}\,}}\left( \textsf{K}_z' \left( \frac{\Delta _z\eta }{r^2} \right) \right) }\left( {{{\,\mathrm{Op^{BW}}\,}}\left( r^{-2}\right) }\Delta _z\eta + {{{\,\mathrm{Op^{BW}}\,}}\left( \Delta _z \eta \right) }\left[ r^{-2}-1 \right] \right) \\&= {{{\,\mathrm{Op^{BW}}\,}}\left( r^{-2}\textsf{K}_z' \left( \frac{\Delta _z\eta }{r^2} \right) \right) } \Delta _z\eta + {{{\,\mathrm{Op^{BW}}\,}}\left( \tilde{V}^0 \left( \eta ;x,z \right) \right) } \eta + R\left( \eta , g ;z \right) \eta , \end{aligned} \end{aligned}$$

where

$$\begin{aligned}\tilde{V}^0(\eta ;x,z)\triangleq r^{-2}\big (K_z'(X)X\big )|_{X=\frac{\Delta _z\eta }{r^2} }.\end{aligned}$$

We plug Eq. (4.23) and (4.21) in Eq. (4.20) and the resulting equation and Eq. (4.19) in Eq. (4.18) and obtain, after using

$$\begin{aligned}\int _{{\mathbb {T}}}\frac{1}{\tan (z/2)}\text {d}z=0 \end{aligned}$$

and applying Proposition 3.26, Item 1, that

4.24 $$\begin{aligned} \begin{aligned} \left( \textsf{K}_z \left( \frac{\Delta _z\eta }{r^2} \right) - 2 \right) g\left( x-z \right)&= {{{\,\mathrm{Op^{BW}}\,}}\left( \textsf{K}_z \left( \frac{\Delta _z\eta }{r^2} \right) - 2\right) } g\left( x-z \right) \\&\quad + {{{\,\mathrm{Op^{BW}}\,}}\left( \frac{g\left( x-z \right) }{r^2}\textsf{K}_z' \left( \frac{\Delta _z\eta }{r^2} \right) \right) } \Delta _z\eta \\&\quad + {{{\,\mathrm{Op^{BW}}\,}}\left( V^0\left( \eta , g ;x,z \right) \right) } \eta + {\boldsymbol{R}}\left( \eta , g;z \right) \begin{bmatrix} \eta \\ g \end{bmatrix} \cdot \begin{bmatrix} 1 \\ 1 \end{bmatrix} , \end{aligned} \end{aligned}$$

where

$$\begin{aligned}V^0(\eta ,g;x,z)\triangleq \tilde{V}^0(\eta ;x,z)g(x-z).\end{aligned}$$

We Taylor expand in *z*

$$\begin{aligned}V^0(\eta ,g;x,z)=\overline{V}_0(\eta ,g;x)+\overline{V}_1(\eta ,g;x,z),\qquad \overline{V}_1\in \Sigma K\mathcal {F}^{1}_{K, 0, 1}\left[ \epsilon _0, N \right] .\end{aligned}$$

We thus plug (4.24) into (4.14) and insert the resulting equation in (4.12) and, after application of Remark 3.22 and Lemma 3.27 we obtain that4.25where

$$\begin{aligned}V(\eta ,g;x)\triangleq \int _{{\mathbb {T}}}\frac{\overline{V}_1(\eta ,g;x,z)}{2\tan (z/2)}\text {d}z.\end{aligned}$$

We use now the identity $\frac{1}{4\sin \left( z/2 \right) \ \tan \left( z/2 \right) } =\frac{1}{4\sin ^2\left( z/2 \right) } - \frac{1}{8\cos ^2 \left( z/4 \right) }$ and Proposition 3.28 and Remark 3.22 in order to transform

4.26 $$\begin{aligned} \int _{{\mathbb {T}}}{{{\,\mathrm{Op^{BW}}\,}}\left( \frac{g\left( x-z \right) }{r^2}\textsf{K}_z' \left( \frac{\Delta _z\eta }{r^2} \right) \right) } \frac{\delta _z\eta }{4\sin \left( z/2 \right) \ \tan \left( z/2 \right) }\text {d}z \nonumber \\ = \int _{{\mathbb {T}}}{{{\,\mathrm{Op^{BW}}\,}}\left( \frac{g\left( x-z \right) }{r^2}\textsf{K}_z' \left( \frac{\Delta _z\eta }{r^2} \right) \right) } \frac{\delta _z\eta }{4\sin ^2\left( z/2 \right) }\text {d}z\nonumber \\ + {{{\,\mathrm{Op^{BW}}\,}}\left( V\left( \eta , g;x \right) + A_{\left[ -2 \right] }\left( \eta , g;x, \xi \right) \right) } \eta + R\left( \eta , g \right) \eta . \end{aligned}$$

Putting (4.26) into (4.25) we obtain that4.27Now, we can use the Taylor-like expansions (4.17), Proposition 3.28 and the fact that *g* has zero average and obtain that4.28Next, denoting

$$\begin{aligned} \tilde{\textsf{K}}\left( \eta , g;x, z \right) \triangleq \frac{g\left( x-z \right) }{1+2\eta }\textsf{K}_z' \left( \frac{\Delta _z\eta }{1+2\eta } \right) - \frac{g}{1+2\eta }\textsf{K}^{\prime 0} \left( \eta ;x \right) \in \Sigma K\mathcal {F}^1_{K, 0, 1}\left[ \epsilon _0, N \right] , \end{aligned}$$

we Taylor-expand $z\mapsto \tilde{\textsf{K}}\left( \eta , g;x, z \right)$ obtaining that

$$\begin{aligned} \tilde{\textsf{K}}\left( \eta , g;x, z \right) = \tilde{\textsf{K}}^1\left( \eta , g;x \right) \sin z + V^2\left( \eta , g;x,z \right) , \end{aligned}$$

so that applying Remark 3.22 and Proposition 3.28, we obtain that4.29We plug Eq. (4.29) and (4.28) in Eq. (4.27) and use Proposition 3.15 and obtain the desired quasi-linear expansion4.30Thus, (4.9) can be derive applying Proposition 3.15 to (4.30) combined with . $\square$

#### Proof of Item 2.

From Eq. (4.12) and (4.14) we have that4.31Thus, we apply Lemma 3.19 and obtain that

4.32 $$\begin{aligned} \textsf{K}_z \left( \frac{\Delta _z\eta }{r^2} \right) - 2 = {{{\,\mathrm{Op^{BW}}\,}}\left( \textsf{K}_z' \left( \frac{\Delta _z\eta }{r^2} \right) \right) }\left[ \frac{\Delta _z\eta }{r^2} \right] + R \left( \frac{\Delta _z\eta }{r^2} \right) \left[ \frac{\Delta _z\eta }{r^2} \right] . \end{aligned}$$

Notice that from Proposition 3.26, Item 4 we have that $R \left( \frac{\Delta _z\eta }{r^2} \right) \in \Sigma K\mathcal {R}^{-\varrho , 0}_{K, 0, 1}\left[ \epsilon _0, N \right]$ and by Taylor expansion we have that

$$\begin{aligned} \tilde{R}\left( \eta ;x, z \right) \triangleq R \left( \frac{\Delta _z\eta }{r^2} \right) -R \left( \frac{\eta _x}{r^2} \right) \in \Sigma K\mathcal {R}^{-\varrho , 1 }_{K, 0, 1}\left[ \epsilon _0, N \right] , \end{aligned}$$

and, since $\frac{\Delta _z}{r^2} \in \Sigma K{\mathcal {M}}^{1, 0}_{K, 0, 1}\left[ \epsilon _0, N \right]$ applying Proposition 3.26, Item 2 we obtain that

4.33 $$\begin{aligned} \tilde{R}\left( \eta ;x, z \right) \circ \frac{\Delta _z}{r^2}\in \Sigma K\mathcal {R}^{-\varrho +1, 1 }_{K, 0, 1}\left[ \epsilon _0, N \right] , \end{aligned}$$

so that, thanks to (4.33), we have that

$$\begin{aligned}  &   \int _{{\mathbb {T}}}R \left( \frac{\Delta _z\eta }{r^2} \right) \left[ \frac{\Delta _z\eta }{r^2} \right] \frac{\text {d}z}{2\tan \left( z/2 \right) } = R\left( \frac{\eta _x}{r^2} \right) \left[ \frac{1}{r^2} \ \int _{{\mathbb {T}}}\frac{\delta _z\eta }{4\sin \left( z/2 \right) \tan \left( z/2 \right) }\text {d}z \right] \\  &   \quad + \int _{{\mathbb {T}}}R\left( \eta ;x, z \right) \text {d}z \ \eta .\end{aligned}$$

Next we use the fact that

$$\begin{aligned}\int _{{\mathbb {T}}}\frac{\delta _z\eta }{4\sin \left( z/2 \right) \tan \left( z/2 \right) }\text {d}z =m_1\left( D \right) \eta ,\qquad \text {with}\qquad m_1\left( \xi \right) \in \widetilde{\Gamma }^1_0,\end{aligned}$$

the fact that $r^{-2} m_1\left( D \right) \in \Sigma K{\mathcal {M}}^{1, 0}_{K,0,1}\left[ \epsilon _0, N \right]$, Proposition 3.26, Item 2 and Lemma 3.27 to obtain that

4.34 $$\begin{aligned} \int _{{\mathbb {T}}}R \left( \frac{\Delta _z\eta }{r^2} \right) \left[ \frac{\Delta _z\eta }{r^2} \right] \frac{\text {d}z}{2\tan \left( z/2 \right) } = R\left( \eta \right) \eta . \end{aligned}$$

Next, computations similar to the ones performed in Eqs. (4.20) and (4.23) allow us to deduce that

4.35 $$\begin{aligned} \begin{aligned} \int _{{\mathbb {T}}}{{{\,\mathrm{Op^{BW}}\,}}\left( \textsf{K}_z' \left( \frac{\Delta _z\eta }{r^2} \right) \right) }\left[ \frac{\Delta _z\eta }{r^2} \right] \frac{\text {d}z}{2\tan \left( z/2 \right) }&= \int _{{\mathbb {T}}}{{{\,\mathrm{Op^{BW}}\,}}\left( r^{-2} \textsf{K}_z' \left( \frac{\Delta _z\eta }{r^2} \right) \right) }\left[ \frac{\delta _z\eta }{4\sin \left( z/2 \right) \tan \left( z/2 \right) } \right] \text {d}z \\&\quad +{{{\,\mathrm{Op^{BW}}\,}}\left( V\left( \eta ;x \right) \right) }\eta + R\left( \eta \right) \eta . \end{aligned} \end{aligned}$$

Taylor-expanding as in (4.29) and using Proposition 3.15 we obtain that

4.36 $$\begin{aligned} \begin{aligned}&\int _{{\mathbb {T}}}{{{\,\mathrm{Op^{BW}}\,}}\left( r^{-2} \textsf{K}_z' \left( \frac{\Delta _z\eta }{r^2} \right) \right) }\left[ \frac{\delta _z\eta }{4\sin \left( z/2 \right) \tan \left( z/2 \right) } \right] \text {d}z \\&= {{{\,\mathrm{Op^{BW}}\,}}\left( r^{-2}\textsf{K}^{\prime 0}\left( \eta ;x \right) \right) }\left| D \right| \eta + {{{\,\mathrm{Op^{BW}}\,}}\left( A_{\left[ 0 \right] }\left( \eta ;x,\xi \right) \right) }\eta + R\left( \eta \right) \eta \\&= {{{\,\mathrm{Op^{BW}}\,}}\left( r^{-2}\textsf{K}^{\prime 0}\left( \eta ;x \right) \left| \xi \right| + A_{\left[ 0 \right] }\left( \eta ;x,\xi \right) \right) }\eta + R\left( \eta \right) \eta . \end{aligned} \end{aligned}$$

Combining Equations (4.32) and (4.34) to (4.36) we obtain (4.11). $\square$

#### Proof of Lemma 4.7.

Let us prove at first (4.16). We prove the result for $\textsf{K}_z$ only being the procedure for $\textsf{K}_z'$ the same. Notice that from Eq. (4.13) we immediately have that the map $\left( z, \textsf{X} \right) \mapsto \textsf{K}_z\left( \textsf{X} \right)$ is analytic in ${\mathbb {T}}\times \left( -\frac{1}{4}, \frac{1}{4} \right)$. Let us now define that

$$\begin{aligned} \overline{\textsf{K}}_z \left( \textsf{x}, \textsf{y} \right) \triangleq \textsf{K}_z\left( \frac{\textsf{y}}{1+2\textsf{x}} \right) , \end{aligned}$$

which is again analytic for $\left| \textsf{x} \right| , \left| \textsf{y} \right| < \varepsilon _1$ small. By analyticity we have that

$$\begin{aligned} \overline{\textsf{K}}_z \left( \textsf{x}, \textsf{y} \right) = \sum _{p_1, p_2 =0}^\infty \underbrace{\frac{\partial _\textsf{x}^{p_2}\partial _\textsf{y}^{p_1} \overline{\textsf{K}}_z \left( 0,0 \right) }{p_1!p_2!}}_{\triangleq \textsf{k}_{p_1, p_2}\left( z \right) } \textsf{x}^{p_2} \textsf{y}^{p_1}, \end{aligned}$$

with

$$\begin{aligned} \left| \partial _\textsf{x}^{p_2}\partial _\textsf{y}^{p_1} \overline{\textsf{K}}_z \left( 0,0 \right) \right| \leqslant C p_1!p_2! \ \varepsilon _1 ^{-\left( p_1 + p_2 \right) }. \end{aligned}$$

From Eq. (4.13) it is immediate that

$$\begin{aligned} \textsf{K}_z\left( 0 \right) = 2, \end{aligned}$$

so that

$$\begin{aligned} \textsf{K}_z\left( \frac{\Delta _z \eta }{1+2\eta } \right) = \overline{\textsf{K}}_z \left( \eta , \Delta _z \eta \right) =&\ 2+ \sum _{p\geqslant 1} \underbrace{\sum _{\begin{array}{c} p_1 \geqslant 1 \\ p_1 + p_2 = p \end{array}} \textsf{k}_{ p_1, p_2}\left( z \right) \ \eta ^{p_2} \left( \Delta _z \eta \right) ^{p_1} }_{ {=: \widetilde{\textsf{K}}^{p} \left( f;x, z \right) } }\\ =&\ 2+ \sum _{p = 1}^N \widetilde{\textsf{K}}^{p} \left( \eta ;x, z \right) + \underbrace{\sum _{p> N} \widetilde{\textsf{K}}^{p} \left( \eta ;x, z \right) }_{ =: \textsf{K}^{> N} \left( \eta ;x, z \right) } \, . \end{aligned}$$

We claim that for any $p\in {\mathbb {N}}$ and $\ell =0,\ldots , 7$,

4.37 $$\begin{aligned} \partial _z^\ell \widetilde{\textsf{K}}^{p} \left( \eta ; x, z \right) \  &\in \widetilde{K\mathcal {F}}^0_p , \end{aligned}$$

4.38 $$\begin{aligned} \partial _z^\ell \textsf{K}^{ > N} \left( \eta ;x, z \right)&\in K\mathcal {F}^0_{K, 0, N +1}\left[ \epsilon _0 \right] . \end{aligned}$$

The proof of Eqs. (4.37) and (4.38) is the same as the one performed in order to prove [21, Eqs. (4.34) and (4.35)] and is thus omitted. The proof for (4.16) for $\textsf{K}'_z\left( \frac{\Delta _z\eta }{1+2\eta } \right)$ is the same as the one performed for $\textsf{K}_z\left( \frac{\Delta _z\eta }{1+2\eta } \right)$ with the sole difference that $\textsf{K}'_z\left( 0 \right) =0$.

The proof of (4.17) follows the lines of the proof of [21, Eq. (4.23)], and is generic enough to be applied in the present case. It remains to prove Eq. (4.10), we can Taylor-expand in $z=0$ and obtain that

$$\begin{aligned} \begin{aligned} \textsf{K}_z\left( \textsf{X} \right) =&\ \textsf{K}_0\left( \textsf{X} \right) + \left. \partial _z \textsf{K}_z\left( \textsf{X} \right) \right| _{z=0} z + R_1 \left( \textsf{K};\textsf{X} \right) \left( z \right) , \\ \textsf{K}'_z\left( \textsf{X} \right) =&\ \textsf{K}'_0\left( \textsf{X} \right) + \left. \partial _z \textsf{K}'_z\left( \textsf{X} \right) \right| _{z=0} z + R_1 \left( \textsf{K}' ;\textsf{X} \right) \left( z \right) . \end{aligned} \end{aligned}$$

Explicit computations show that

$$\begin{aligned} \begin{aligned} \textsf{K}_0\left( \textsf{X} \right) \triangleq&\ 2 - \frac{2\textsf{X}^2}{1+\textsf{X}^2},&\left. \partial _z \textsf{K}_z\left( \textsf{X} \right) \right| _{z=0} \triangleq&-\frac{4\textsf{X}^3}{\left( 1+\textsf{X}^2 \right) ^2}, \\ \textsf{K}_0'\left( \textsf{X} \right) \triangleq&\ -\frac{4\textsf{X}}{\left( 1+\textsf{X}^2 \right) ^2},&\left. \partial _z \textsf{K}'_z\left( \textsf{X} \right) \right| _{z=0} \triangleq&\ -\frac{4\textsf{X}^2\left( 3-\textsf{X}^2 \right) }{\left( 1+\textsf{X}^2 \right) ^3}\cdot \end{aligned} \end{aligned}$$

Hence setting $\textsf{X}\triangleq \frac{\Delta _z\eta }{1+2\eta }$ and Taylor expanding we obtain that

$$\begin{aligned} \begin{aligned} \textsf{K}_0 \left( \frac{\Delta _z\eta }{1+2\eta } \right) =&\ 2 \underbrace{- 2\frac{\eta _x^2}{\left( 1+2\eta \right) ^2 + \eta _x^2}}_{\triangleq \textsf{K}^0\left( \eta ; x \right) } + 4\frac{\left( 1+2\eta \right) ^2\eta _x\eta _{xx}}{\left( \left( 1+2\eta \right) ^2 + \eta _x^2 \right) ^2} z + V^2\left( \eta ;x,z \right) , \\ \textsf{K}'_0 \left( \frac{\Delta _z\eta }{1+2\eta } \right) =&\ \underbrace{-\frac{4\left( 1+2\eta \right) \eta _x}{\left( \left( 1+2\eta \right) ^2 + \eta _x^2 \right) ^2}}_{\triangleq \textsf{K}^{\prime 0}\left( \eta ;x \right) } + 2\frac{\left( \left( 1+2\eta \right) ^2 -3\eta _x^2 \right) \left( 1+2\eta \right) ^3\eta _{xx}}{\left( \left( 1+2\eta \right) ^2 + \eta _x^2 \right) ^3} z + V^2\left( \eta ;x,z \right) , \\ \left. \partial _z \textsf{K}_z\left( \frac{\Delta _x \eta }{1+2\eta } \right) \right| _{z=0} \triangleq&-\frac{4\left( 1+2\eta \right) \eta _x^3}{\left( \left( 1+2\eta \right) ^2+\eta _x^2 \right) ^2} + R_0 \left( \left. \partial _z \textsf{K}_z\left( \frac{\Delta _x \eta }{1+2\eta } \right) \right| _{z=0} ;x \right) \left( z \right) , \\ \left. \partial _z \textsf{K}'_z\left( \frac{\Delta _x \eta }{1+2\eta } \right) \right| _{z=0} \triangleq&\ -\frac{4\left( 1+2\eta \right) ^2\eta _x^2\left( 3\left( 1+2\eta \right) ^2-\eta _x^2 \right) }{\left( \left( 1+2\eta \right) ^2+\eta _x^2 \right) ^3}+ R_0 \left( \left. \partial _z \textsf{K}'_z\left( \frac{\Delta _x \eta }{1+2\eta } \right) \right| _{z=0} ;x \right) \left( z \right) , \end{aligned} \end{aligned}$$

so that defining

$$\begin{aligned} \textsf{K}^1\left( \eta ;x \right) \triangleq&\ \frac{4\left( 1+2\eta \right) ^2\eta _x\eta _{xx}}{\left( \left( 1+2\eta \right) ^2 + \eta _x^2 \right) ^2} -\frac{4\left( 1+2\eta \right) \eta _x^3}{\left( \left( 1+2\eta \right) ^2+\eta _x^2 \right) ^2}=\frac{4\left( 1+2\eta \right) \eta _x\left( \left( 1+2\eta \right) \eta _{xx}-\eta _x^2 \right) }{\left( \left( 1+2\eta \right) ^2+\eta _x^2 \right) ^2}, \\ \textsf{K}^{\prime 1}\left( \eta ;x \right) \triangleq&\ \frac{2\left( \left( 1+2\eta \right) ^2 -3\eta _x^2 \right) \left( 1+2\eta \right) ^3\eta _{xx}-4\left( 1+2\eta \right) ^2\eta _x^2\left( 3\left( 1+2\eta \right) ^2-\eta _x^2 \right) }{\left( \left( 1+2\eta \right) ^2 + \eta _x^2 \right) ^3}, \end{aligned}$$

which are analytic applications w.r.t. $\eta$ small in $W^{2, \infty }$, proving (4.17) and (4.10).

$$\square $$

### Paralinearization of 

Here we perform the paralinearization of the nonlinear operator  introduced in (1.5).

#### Proposition 4.8

Let $N\in {\mathbb {N}}$ and $\varrho \geqslant 0$, for any $K\in {\mathbb {N}}$ there exist $s_0 > 0$ and $\epsilon _0 > 0$ such that if $\eta , g \in B^{K}_{s_0, \mathbb {R}}\left( I;\epsilon _0 \right)$ and. be as in (1.5), we have that

1. 4.39 where
  4.40 $$\begin{aligned} \textsf{J}^0\left( \eta ;x \right)&\triangleq \frac{2\eta _x\left( 1+2\eta \right) }{\left( 1+2\eta \right) ^2+\eta _x^2}\in \mathcal {F}^{{\mathbb {R}}}_{K, 0, 1}\left[ \epsilon _0, N \right] , \end{aligned}$$
  4.41 $$\begin{aligned} \textsf{J}^{\prime 0}\left( \eta ;x \right)&\triangleq \frac{2\left( 1+2\eta \right) ^2\left( \left( 1+2\eta \right) ^2-\eta _x^2 \right) }{\left( \left( 1+2\eta \right) ^2+\eta _x^2 \right) ^2}\in \mathcal {F}^{{\mathbb {R}}}_{K, 0, 0}\left[ \epsilon _0, N \right] , \end{aligned}$$
  $$ A_{\left[ m \right] }\left( \eta , g;x, \xi \right) \in \Sigma \Gamma ^{m} _{K, 0, 1}\left[ \epsilon _0, N \right] $$ and $$ {\boldsymbol{R}}\left( \eta , g \right) \in \left( \Sigma \mathcal {R}^{-\varrho } _{K, 0, 1}\left[ \epsilon _0, N \right] \right) ^{2\times 2} $$.
2. 4.42 where
  $\textsf{J}^{\prime 0}\left( \eta ; x \right)$ is explicitly defined in (4.41), and is such that $\textsf{J}^{\prime 0}\left( 0;x \right) \equiv 2$;
  $$ A_{\left[ 0 \right] }\left( \eta ;x, \xi \right) \in \Sigma \Gamma ^{0} _{K, 0, 1}\left[ \epsilon _0, N \right] $$;
  $$ R\left( \eta \right) \in \Sigma \mathcal {R}^{-\varrho } _{K, 0, 1}\left[ \epsilon _0, N \right] $$.

#### Proof of Proposition 4.8, Item 1.

Let us recall that  is defined in (1.5). We proceed analogously as in the previous section. We have, using the auxiliary functions defined in (4.6) and the fact that *g* is of zero average, that4.43where

$$\begin{aligned} \textsf{H}_z\left( \textsf{X} \right) \triangleq \frac{1-\sqrt{1-2\textsf{X}}\cos z}{1-\textsf{X}- \sqrt{1-2\textsf{X}}\cos z} \ 2\sin \left( z/2 \right) . \end{aligned}$$

We define

4.44 $$\begin{aligned} \textsf{J}_z\left( \textsf{X} \right) \triangleq \textsf{H}_z\left( \textsf{X}\ 2\sin \left( z/2 \right) \right) . \end{aligned}$$

We have the following technical result:

#### Lemma 4.9

Let $\textsf{J}_z (\textsf{X})$, be as in Eq. (4.44). Then

4.45 $$\begin{aligned}&\textsf{J}_z \left( \frac{\Delta _z \eta }{1+2\eta } \right) _{\geqslant 1} \triangleq \textsf{J}_z \left( \frac{\Delta _z \eta }{1+2\eta } \right) - 2\sin \left( z/2 \right) \in \Sigma K\mathcal {F}^{0}_{K, 0, 1}\left[ \epsilon _0, N \right] , \nonumber \\&\textsf{J}_z' \left( \frac{\Delta _z \eta }{1+2\eta } \right) \in \Sigma K\mathcal {F}^{0}_{K, 0, 0}\left[ \epsilon _0, N \right] , \end{aligned}$$

are Kernel functions, which admit the expansion

4.46 $$\begin{aligned} \begin{aligned} \textsf{J}_z \left( \frac{\Delta _z \eta }{1+2\eta } \right) - 2\sin \left( z/2 \right) =&\ \textsf{J}^0 \left( \eta ;x \right) +\textsf{J}^1\left( \eta ;x \right) \ 2\sin \left( z/2 \right) + V^2 \left( \eta ;x,z \right) , \\ \textsf{J}'_z \left( \frac{\Delta _z \eta }{1+2\eta } \right) =&\ \textsf{J}^{\prime 0} \left( \eta ;x \right) +\textsf{J}^{\prime 1 }\left( \eta ;x \right) \sin z + V^2 \left( \eta ;x,z \right) , \end{aligned} \end{aligned}$$

where $\textsf{J}^{0}\left( \eta ;x \right) ,\textsf{J}^{\prime 0}\left( \eta ;x \right)$ are defined in (4.40)-(4.41) and $\textsf{J}^1\left( \eta ;x \right) , \textsf{J}^{\prime 1}\left( \eta ;x \right) \in \Sigma \mathcal {F}^{{\mathbb {R}}}_{K, 0, 1}\left[ \epsilon _0, N \right] .$

The proof of Lemma 4.9 follows exactly the same lines of the proof of Lemma 4.7 (cf. page 35) and is thus omitted for the sake of brevity.

We apply Lemma 3.16 and obtain that

4.47 $$\begin{aligned} \begin{aligned}&\frac{1}{r^2}\textsf{J}_z \left( \frac{\Delta _z\eta }{r^2} \right) _{\geqslant 1} g\left( x-z \right) \\&\quad = {{{\,\mathrm{Op^{BW}}\,}}\left( \frac{1}{r^2}\textsf{J}_z \left( \frac{\Delta _z\eta }{r^2} \right) \right) }_{\geqslant 1} g\left( x-z \right) + {{{\,\mathrm{Op^{BW}}\,}}\left( g\left( x-z \right) \right) }\left[ \frac{1}{r^2}\textsf{J}_z \left( \frac{\Delta _z \eta }{1+2\eta } \right) _{\geqslant 1} \right] \\&\qquad + R_1 \left( \frac{1}{r^2}\textsf{J}_z \left( \frac{\Delta _z \eta }{1+2\eta } \right) _{\geqslant 1} \right) g\left( x-z \right) + R_2 \left( g\left( x-z \right) \right) \left[ \frac{1}{r^2}\textsf{J}_z \left( \frac{\Delta _z \eta }{1+2\eta } \right) _{\geqslant 1} \right] . \end{aligned} \end{aligned}$$

In view of (4.45) we can apply Proposition 3.26, Items 4 and 2 giving that

4.48 $$\begin{aligned}&R_1 \left( \frac{1}{r^2}\textsf{J}_z \left( \frac{\Delta _z \eta }{1+2\eta } \right) _{\geqslant 1} \right) g\left( x-z \right) = R\left( \eta , g ;z \right) g, \nonumber \\&R_2 \left( g\left( x-z \right) \right) \left[ \frac{1}{r^2}\textsf{J}_z \left( \frac{\Delta _z \eta }{1+2\eta } \right) _{\geqslant 1} \right] = R\left( \eta , g ;z \right) \eta . \end{aligned}$$

Then, we apply Lemmas 3.19 and 3.16 and Proposition 3.15 in a similar fashion to the procedure detailed in the previous section and get that

4.49 $$\begin{aligned}&{{{\,\mathrm{Op^{BW}}\,}}\left( g\left( x-z \right) \right) }\left[ \frac{1}{r^2}\textsf{J}_z \left( \frac{\Delta _z \eta }{1+2\eta } \right) _{\geqslant 1} \right] \nonumber \\&\quad = {{{\,\mathrm{Op^{BW}}\,}}\left( \frac{g\left( x-z \right) }{r^4}\textsf{J}'_z\left( \frac{\Delta _z\eta }{r^2} \right) \right) } \Delta _z\eta \nonumber \\&\qquad + {{{\,\mathrm{Op^{BW}}\,}}\left( V^0\left( \eta , g;x, z \right) \right) } \eta + R\left( \eta , g;z \right) \eta . \end{aligned}$$

Next, we use the expansions of Lemma 4.9 and obtain that

4.50 $$\begin{aligned} \begin{aligned} {{{\,\mathrm{Op^{BW}}\,}}\left( \frac{1}{r^2}\textsf{J}_z \left( \frac{\Delta _z \eta }{1+2\eta } \right) _{\geqslant 1}\right) } g\left( x-z \right) =&\ {{{\,\mathrm{Op^{BW}}\,}}\left( \frac{\textsf{J}^0\left( \eta ;x \right) }{r^2} + V^1\left( \eta , g;x, z \right) \right) }g\left( x-z \right) , \\ {{{\,\mathrm{Op^{BW}}\,}}\left( \frac{g\left( x-z \right) }{r^4}\textsf{J}'_z\left( \frac{\Delta _z\eta }{r^2} \right) \right) } \Delta _z\eta =&\ {{{\,\mathrm{Op^{BW}}\,}}\left( \frac{g}{r^4}\textsf{J}^{\prime 0}\left( \eta ; x \right) \right) }\Delta _z\eta \\&\ + {{{\,\mathrm{Op^{BW}}\,}}\left( V\left( \eta , g;x \right) + V^1\left( \eta , g;x, z \right) \right) }\delta _z\eta , \\ {{{\,\mathrm{Op^{BW}}\,}}\left( V^0\left( \eta , g;x, z \right) \right) } \eta =&\ {{{\,\mathrm{Op^{BW}}\,}}\left( V\left( \eta , g;x \right) + V^1\left( \eta , g;x, z \right) \right) }\eta . \end{aligned} \end{aligned}$$

We plug the results in Eqs. (4.50) and (4.49) to (4.44) into Eq. (4.43) and obtain, after an application of Proposition 3.28, thatfrom which we conclude after standard symbolic manipulations the identity in (4.39). $\square$

#### Proof of Proposition 4.8, Item 2.

Arguing similarly as it was done in order to deduce (4.43) and using Eqs. (4.44) and (4.46), we have that4.51We apply Lemma 3.19 and obtain that4.52Thus, computations similar to the ones that lead to (4.34) give us that

4.53 $$\begin{aligned} \int _{{\mathbb {T}}}\frac{1}{r^2} R\left( \frac{\Delta _z\eta }{r^2} \right) \left[ \frac{\Delta _z\eta }{r^2} \right] \frac{\text {d}z}{2\sin \left( z/2 \right) } = R \left( \eta \right) \eta . \end{aligned}$$

Next, similar computations to the ones that lead to (4.35) give us that

4.54 $$\begin{aligned} \begin{aligned}&\int _{{\mathbb {T}}}\frac{1}{r^2}{{{\,\mathrm{Op^{BW}}\,}}\left( \textsf{J}_z' \left( \frac{\Delta _z\eta }{r^2} \right) \right) }\left[ \frac{\Delta _z\eta }{r^2} \right] \frac{\text {d}z}{2\sin \left( z/2 \right) }\\&\quad = \int _{{\mathbb {T}}}{{{\,\mathrm{Op^{BW}}\,}}\left( r^{-4} \textsf{J}_z' \left( \frac{\Delta _z\eta }{r^2} \right) \right) }\left[ \frac{\delta _z\eta }{\left( 2\sin \left( z/2 \right) \right) ^2 } \right] \text {d}z \\&\qquad +{{{\,\mathrm{Op^{BW}}\,}}\left( V\left( \eta ;x \right) \right) }\eta + R\left( \eta \right) \eta , \end{aligned} \end{aligned}$$

so that arguing as in order to deduce (4.36)

4.55 $$\begin{aligned} \begin{aligned}&\int _{{\mathbb {T}}}{{{\,\mathrm{Op^{BW}}\,}}\left( r^{-4} \textsf{J}_z' \left( \frac{\Delta _z\eta }{r^2} \right) \right) }\left[ \frac{\delta _z\eta }{\left( 2\sin \left( z/2 \right) \right) ^2 } \right] \text {d}z \\&= {{{\,\mathrm{Op^{BW}}\,}}\left( r^{-4}\textsf{J}^{\prime 0}\left( \eta ;x \right) \right) }\left| D \right| \eta + {{{\,\mathrm{Op^{BW}}\,}}\left( A_{\left[ 0 \right] }\left( \eta ;x,\xi \right) \right) }\eta + R\left( \eta \right) \eta \\&= {{{\,\mathrm{Op^{BW}}\,}}\left( r^{-4}\textsf{J}^{\prime 0}\left( \eta ;x \right) \left| \xi \right| + A_{\left[ 0 \right] }\left( \eta ;x,\xi \right) \right) }\eta + R\left( \eta \right) \eta . \end{aligned} \end{aligned}$$

Thus, combining Eqs. (4.51) to (4.55) and the paralinearization $r^{-2}=1+{{{\,\mathrm{Op^{BW}}\,}}\left( -\frac{2}{\left( 1+2\eta \right) ^2}\right) }\eta +R\left( \eta \right) \eta$ derived using Lemma 3.19 we obtain Eq. (4.42). $\square$

### Paralinearization of 

In view of (4.7), putting together the paralinearizations of Sections 4.1 and 4.2, we prove the following:

#### Lemma 4.10

Let $N\in {\mathbb {N}}$, ${\texttt{b}}\in {\mathbb {R}}$ and $\varrho \geqslant 0$, for any $K\in {\mathbb {N}}$ there exist $s_0 > 0$ and $\epsilon _0 > 0$ such that if $\eta , g \in B^{K}_{s_0, \mathbb {R}}\left( I;\epsilon _0 \right)$, let  be as in (4.7) then we have that

1. 4.56 where
  $\textsf{J}^0\left( \eta ; x \right) \in \mathcal {F}^{{\mathbb {R}}}_{K, 0, 1}\left[ \epsilon _0, N \right]$ and is explicitly defined in (4.40);
  , cf. (1.5);
  $$ A_{\left[ m \right] }\left( \eta , g;x, \xi \right) \in \Sigma \Gamma ^{m} _{K, 0, 1}\left[ \epsilon _0, N \right] $$;
  $$ R\left( \eta , g \right) \in \Sigma \mathcal {R}^{-\varrho } _{K, 0, 1}\left[ \epsilon _0, N \right] $$.
2. 4.57 where
  $\textsf{J}^0\left( \eta ; x \right) \in \mathcal {F}^{{\mathbb {R}}}_{K, 0, 1}\left[ \epsilon _0, N \right]$ and is explicitly defined in (4.40);
  with $$ \tilde{V}\left( 0;x \right) \equiv 1$$;
  $$ A_{\left[ 0 \right] }\left( \eta ;x, \xi \right) \in \Sigma \Gamma ^{0} _{K, 0, 1}\left[ \epsilon _0, N \right] $$;
  $$ R\left( \eta \right) \in \Sigma \mathcal {R}^{-\varrho } _{K, 0, 1}\left[ \epsilon _0, N \right] $$.

#### Proof

We use now the expression in Eq. (4.7) and Propositions 4.8 and 4.6 as well as Proposition 3.15 and obtain that4.58Notice that in order to deduce (4.58) we used the fact that , which stems immediately from the paralinearization provided in Proposition 4.8, Item 1, next we relabeled . Next we use Equations (4.10), (4.40) and (4.41) and obtain that

$$\begin{aligned} \begin{aligned} \textsf{K}^0\left( \eta ;x \right) + \frac{\eta _x}{r^2} \ \textsf{J}^0\left( \eta ;x \right) =&\ 0, \\ \textsf{K}^{\prime 0}\left( \eta ;x \right) + \frac{\eta _x}{r^2} \ \textsf{J}^{\prime 0}\left( \eta ;x \right) =&\ -\textsf{J}^0\left( \eta ;x \right) . \end{aligned} \end{aligned}$$

Thus, transforming (4.58) into (4.56). The proof of (4.57) is almost identical to the proof of (4.56) and is hence omitted. $\square$

### Proof of Proposition 4.2

We can finally paralinearize Eq. (1.9). We use Equations (4.56) and (4.57), we set $g = \partial _x \psi$ and Proposition 3.15 in order to obtain, from (1.9), that

4.59 $$\begin{aligned} \begin{aligned} \eta _t&= {{{\,\mathrm{Op^{BW}}\,}}\left( -\frac{\left| \xi \right| }{2} + A_{\left[ -2 \right] }\left( \eta ;x, \xi \right) \right) }\psi \\&\quad +{{{\,\mathrm{Op^{BW}}\,}}\left( \frac{1}{2}B_{\texttt{b}}\left( \eta , \psi ;x \right) \left| \xi \right| - \textrm{i}\ V_{\texttt{b}}\left( \eta , \psi ;x \right) \xi + A_{\left[ 0 \right] }\left( \eta , \psi ;x, \xi \right) \right) }\eta \\&\quad + R\left( \eta , \psi \right) \left[ \eta +\psi \right] , \end{aligned} \end{aligned}$$

where

4.60 $$\begin{aligned}&B_{\texttt{b}}\left( \eta , \psi ;x \right) \triangleq B_0 \left( \eta , \psi ;x \right) + {\texttt{b}}\tilde{B} \left( \eta ;x \right) , B_0 \left( \eta , \psi ;x \right) \triangleq \frac{ \psi _x}{1+2\eta } \textsf{J}^0\left( \eta ;x \right) ,\nonumber \\&\tilde{B} \left( \eta ;x \right) \triangleq \frac{\textsf{J}^0\left( \eta ;x \right) }{1+2\eta } \end{aligned}$$

andLet us now paralinearize the termWe apply Lemma 3.16 and obtain that4.61Recall that , so that we can apply Propositions 3.17 and 3.15 and obtain that4.62Next we apply the paralinearization stated in Proposition 4.8 and the composition theorems in Propositions 3.17 and 3.15 and obtain that4.63so, using (4.60), we obtain that4.64We invoke now Proposition 4.8 and obtain, using the notation introduced in (4.60), that4.65Notice that using Eqs. (4.62) and (4.61) we obtain that4.66From Equations (4.40) and (4.41) we obtain the relation

4.67 $$\begin{aligned} \textsf{J}^{\prime 0}\left( \eta ;x \right) {=\frac{2\left( 1+2\eta \right) ^4}{\left( \left( 1+2\eta \right) ^2+\eta _x^2 \right) ^2} - \left( \textsf{J}^0\left( \eta ;x \right) \right) ^2} \end{aligned}$$

so that using Eqs. (4.60), (4.67), (4.64) and (4.65) and denoting with

4.68 $$\begin{aligned} W_{\texttt{b}}\left( \eta , \psi ;x \right) \triangleq W_0\left( \eta , \psi ;x \right) + {\texttt{b}}\tilde{W}\left( \eta ;x \right) \in \Sigma \mathcal {F}^{{\mathbb {R}}}_{K, 0, 0}\left[ \epsilon _0, N \right] , \end{aligned}$$

where

4.69 $$\begin{aligned}&W_0\left( \eta , \psi ;x \right) \triangleq \frac{\psi _x\left( 1+2\eta \right) }{\left( 1+2\eta \right) ^2+\eta _x^2}\in \Sigma \mathcal {F}^{{\mathbb {R}}}_{K, 0, 1}\left[ \epsilon _0, N \right] , \nonumber \\&\tilde{W}\left( \eta ;x \right) \triangleq \frac{1+2\eta }{\left( 1+2\eta \right) ^2+\eta _x^2}\in \Sigma \mathcal {F}^{{\mathbb {R}}}_{K, 0, 0}\left[ \epsilon _0, N \right] , \end{aligned}$$

we obtain that4.70We thus insert (4.70) in (4.66) and obtain that4.71An iterated application of Lemma 3.19 gives us that4.72We can thus now plug Eqs. (4.72) and (4.71) into the second equation of (1.9) (recall that $\Omega$ is set in (2.21) in order to cancel the 0-homogeneous components of the transport) and obtain that

4.73 $$\begin{aligned} \begin{aligned} \psi _t&= {{{\,\mathrm{Op^{BW}}\,}}\left( -\frac{1}{2}B_{\texttt{b}}\left( \eta , \psi ;x \right) \left| \xi \right| - \textrm{i}\ V_{\texttt{b}}\left( \eta , \psi ;x \right) \xi + A_{\left[ -2 \right] }\left( \eta ;x, \xi \right) \right) }\psi \\&\quad +{{{\,\mathrm{Op^{BW}}\,}}\left( \gamma \left( 1+\texttt{f}\left( \eta ;x \right) \right) \left( \left| \xi \right| ^2-1 \right) + \frac{{\texttt{b}}^2}{\left( 1+2\eta \right) } -\frac{1}{2}\left( W_{\texttt{b}}^2\left( \eta , \psi ;x \right) - B_{\texttt{b}}^2\left( \eta , \psi ;x \right) \right) \left| \xi \right| + A_{\left[ 0 \right] }\left( \eta , \psi ;x, \xi \right) \right) }\eta \\&\quad + {\boldsymbol{R}}\left( \eta , \psi \right) \begin{bmatrix} \eta \\ \psi \end{bmatrix} \cdot \begin{bmatrix} 1 \\ 1 \end{bmatrix} . \end{aligned} \end{aligned}$$

Finally, we combine Eqs. (4.73) and (4.59) to obtain (4.1) after a renaming, if needed.

## Complex Hamiltonian Formulation of the Kelvin-Helmholtz Equations

We begin with the real Hamiltonian system (2.5), and introduce the following associated complex variables5.1Under this change of variables (2.5) is equivalent to5.2Defining  and noting that

$$\begin{aligned} \begin{bmatrix} \partial _\eta \\ \partial _\psi \end{bmatrix} = \frac{1}{\sqrt{2}} \begin{bmatrix} 1& 1\\ \textrm{i}&  -\textrm{i}\end{bmatrix} \begin{bmatrix} \partial _v \\ \partial _{\bar{v}} \end{bmatrix} , \end{aligned}$$

we obtain that (5.2) can be written as a Hamiltonian system in the complex coordinates (5.1) as

5.3 $$\begin{aligned} V_t = {\boldsymbol{J}}_{\mathbb {C}}\nabla _{U} H_{\mathbb {C}} \left( V \right) , \qquad V\triangleq \begin{bmatrix} v \\ \bar{v} \end{bmatrix} , \end{aligned}$$

where the *complex Poisson tensor* (cf. (2.5)) is defined as

5.4 $$\begin{aligned} {\boldsymbol{J}}_{\mathbb {C}} \triangleq -\textrm{i}{\boldsymbol{J}} = \begin{bmatrix} 0 &  \textrm{i}\\ -\textrm{i}&  0 \end{bmatrix} . \end{aligned}$$

Next we define the Fourier symbol

5.5 $$\begin{aligned} \begin{aligned}&\textsf{m}_{\gamma , {\texttt{b}}}\left( {\xi } \right) \triangleq \sqrt{\frac{\left| \xi \right| }{2\omega _{\gamma , {\texttt{b}}}\left( \xi \right) }} = \root 4 \of {\frac{\left| \xi \right| }{2\left( \gamma \left( \left| \xi \right| ^2 -1 \right) - {\texttt{b}}^2\left( \frac{{|\xi |}}{2}-1 \right) \right) }} \quad \in \tilde{\Gamma }^{-\frac{1}{4}}_0,\\&\textsf{m}_{\gamma , {\texttt{b}}}\left( {\xi } \right) ^{-1}= \root 4 \of {\frac{2\left( \gamma \left( {\xi }^2 -1 \right) - {\texttt{b}}^2\left( \frac{{|\xi |}}{2}-1 \right) \right) }{{|\xi |}}}\quad \in \tilde{\Gamma }^{\frac{1}{4}}_0. \end{aligned} \end{aligned}$$

Then, we consider the symplectic matrix

5.6 $$\begin{aligned} {\boldsymbol{S}}_{\gamma ,{\texttt{b}}}(D)\triangleq { \begin{bmatrix} \textsf{m}_{\gamma , {\texttt{b}}}\left( {D} \right) & 0 \\ 0 &  \textsf{m}_{\gamma , {\texttt{b}}}\left( {D} \right) ^{-1} \end{bmatrix} }\qquad \text {and}\qquad {\boldsymbol{\textsf{M}}} _{\gamma , {\texttt{b}}}\left( {D} \right) \triangleq {\boldsymbol{S}}_{\gamma ,{\texttt{b}}}(D)\circ \mathcal {C}. \end{aligned}$$

Notice that

5.7 $$\begin{aligned} \begin{aligned} {\boldsymbol{\textsf{M}}} _{\gamma , {\texttt{b}}}\left( {\xi } \right) \triangleq&\ \frac{1}{\sqrt{2}} \left[ \begin{array}{cc} \textsf{m}_{\gamma , {\texttt{b}}}\left( {\xi } \right) &  \textsf{m}_{\gamma , {\texttt{b}}}\left( {\xi } \right) \\ - \textrm{i}\textsf{m}_{\gamma , {\texttt{b}}}\left( {\xi } \right) ^{-1} &  \textrm{i}\textsf{m}_{\gamma , {\texttt{b}}}\left( {\xi } \right) ^{-1} \end{array} \right]&\in \left( \tilde{\Gamma }^{1/4}_0 \right) ^{2\times 2}, \\ {\boldsymbol{\textsf{M}}}_{\gamma , {\texttt{b}}}^{-1} \left( {\xi } \right) \triangleq&\ \frac{1}{\sqrt{2}} \left[ \begin{array}{cc} \textsf{m}_{\gamma , {\texttt{b}}}\left( {\xi } \right) ^{-1} &  \textrm{i}\ \textsf{m}_{\gamma , {\texttt{b}}}\left( {\xi } \right) \\ \textsf{m}_{\gamma , {\texttt{b}}}\left( {\xi } \right) ^{-1} &  -\textrm{i}\ \textsf{m}_{\gamma , {\texttt{b}}}\left( {\xi } \right) \end{array} \right]&\in \left( \tilde{\Gamma }^{1/4}_0 \right) ^{2\times 2}. \end{aligned} \end{aligned}$$

We define the *complex coordinates* *U* as

5.8 $$\begin{aligned} U\triangleq \begin{bmatrix} u \\ \bar{u} \end{bmatrix} \triangleq {\boldsymbol{\textsf{M}}}_{\gamma , {\texttt{b}}}^{-1} \left( {D} \right) \begin{bmatrix} \eta \\ \psi \end{bmatrix} =\mathcal {C}^{-1}\circ {\boldsymbol{S}}_{\gamma ,{\texttt{b}}}^{-1}(D) \begin{bmatrix} \eta \\ \psi \end{bmatrix} . \end{aligned}$$

### Notation 5.1

Let $K\in {\mathbb {N}}$, $\epsilon _0 > 0$, $m\in {\mathbb {R}}$, $N\in {\mathbb {N}}$ and $0\leqslant K'\leqslant K$. We work on a time interval $I=[0,T]$ for some fixed $T>0$ to be determined. This latter *is a priori* implicit but will correspond to $c\varepsilon ^{-(N+1)}.$ Moreover, from now on we denote with

- $A_{\left[ m;K' \right] }\left( U; x, \xi \right)$ any generic element in the space $\Sigma \Gamma ^{m}_{K, K', 1 }\left[ \epsilon _0, N \right]$, while ${\boldsymbol{A}}_{\left[ m;K' \right] }\left( U; x, \xi \right)$ is a generic element of $\left( \Sigma \Gamma ^{m}_{K, K', 1 }\left[ \epsilon _0, N \right] \right) ^{2\times 2}$ such that ${\boldsymbol{J}}_{\mathbb {C}}{{{\,\mathrm{Op^{BW}}\,}}\left( {\boldsymbol{A}}_{\left[ m;K' \right] }\left( U;x, \xi \right) \right) }$ is linearly Hamiltonian up to homogeneity *N* (cf. Definition A.2), whose explicit expression may vary from line to line. We use as well the simplified notation $A_{\left[ m;0 \right] }\left( U; x, \xi \right) \triangleq A_{\left[ m \right] }\left( U; x, \xi \right)$ and ${\boldsymbol{A}}_{\left[ m;0 \right] }\left( U; x, \xi \right) \triangleq {\boldsymbol{A}}_{\left[ m \right] }\left( U; x, \xi \right)$;
- $R_{\left[ K' \right] }\left( U \right)$ and ${\boldsymbol{R}}_{\left[ K' \right] }\left( U \right)$ any generic element in the space $\Sigma \mathcal {R}^{-\varrho }_{K, K', 1 }\left[ \epsilon _0, N \right]$ and $\left( \Sigma \mathcal {R}^{-\varrho }_{K, K', 1 }\left[ \epsilon _0, N \right] \right) ^{2\times 2}$ respectively, whose explicit expression may vary from line to line. We denote as well $R_{\left[ 0 \right] }\left( U \right) \triangleq R\left( U \right)$ and ${\boldsymbol{R}}_{\left[ 0 \right] }\left( U \right) \triangleq {\boldsymbol{R}}\left( U \right) .$

We prove the following:

### Proposition 5.2

(Kelvin-Helmholtz equations in complex Hamiltonian coordinates) Let $N\in {\mathbb {N}}$, $\gamma > 0$, ${\texttt{b}}\in {\mathbb {R}}$ and $\varrho \geqslant 0$, for any $K\in {\mathbb {N}}$ there exists $s_0 >0$ and $\epsilon _0 > 0$ such that if $\eta , \psi \in B^K_{s_0, {\mathbb {R}}}\left( I;\epsilon _0 \right)$ is a solution of Eq. (1.9), then *U* defined in (5.8) solves the complex Hamiltonian system

5.9 $$\begin{aligned} U_t = {\boldsymbol{J}}_{\mathbb {C}} \ {{{\,\mathrm{Op^{BW}}\,}}\left( {\boldsymbol{A}}_{\frac{3}{2}}\left( U;x \right) \ \omega _{\gamma , {\texttt{b}}}\left( \xi \right) + {\boldsymbol{A}}_{1}\left( U;x, \xi \right) + {\boldsymbol{A}}_{\frac{1}{2}}\left( U;x \right) \ \left| \xi \right| ^{\frac{1}{2}} + {\boldsymbol{A}}_{\left[ 0 \right] } \left( U;x, \xi \right) \right) } U +{\boldsymbol{R}}\left( U \right) U , \end{aligned}$$

where

- ${\boldsymbol{J}}_{\mathbb {C}}$ is defined in Eq. (5.4);
- ${\boldsymbol{A}}_{\frac{3}{2}}\left( U;x \right) \in \left( \Sigma \mathcal {F}^{{\mathbb {R}}}_{K, 0, 0}\left[ \epsilon _0, N \right] \right) ^{2\times 2}$ is defined as
  5.10 $$\begin{aligned} {\boldsymbol{A}}_{\frac{3}{2}}\left( U, x \right) \triangleq \begin{bmatrix} 0& 1\\ 1& 0 \end{bmatrix} + \frac{\texttt{f}\left( \left( {\boldsymbol{\textsf{M}}} _{\gamma , {\texttt{b}}}\left( D \right) U \right) _1 ;x \right) }{2} \begin{bmatrix} 1 &  1 \\ 1 &  1 \end{bmatrix} , \end{aligned}$$
  where $\texttt{f}\left( \eta ; x \right)$ is defined in Eq. (4.72);
- ${\boldsymbol{A}}_{1}\left( U;x, \xi \right) \in \left( \Sigma \Gamma ^1 _{K, 0, 1}\left[ \epsilon _0, N \right] \right) ^{2\times 2}$ is defined as
  $$\begin{aligned} {\boldsymbol{A}}_{1}\left( U;x,\xi \right) \triangleq - V_{{\texttt{b}}}\left( {\boldsymbol{\textsf{M}}} _{\gamma , {\texttt{b}}}\left( D \right) U;x \right) \xi \begin{bmatrix} 0& -1\\ 1& 0 \end{bmatrix} + \frac{B_{\texttt{b}}\left( {\boldsymbol{\textsf{M}}} _{\gamma , {\texttt{b}}}\left( \left| D \right| \right) U;x \right) }{2} \ \left| \xi \right| \begin{bmatrix} \textrm{i}& 0\\ 0&  -\textrm{i}\end{bmatrix}, \end{aligned}$$
  where $V_{{\texttt{b}}}\left( \eta , \psi ;x \right) \in \Sigma \mathcal {F}^{{\mathbb {R}}}_{K, 0, 1}\left[ \epsilon _0, N \right]$ and $B_{\texttt{b}}\left( \eta , \psi ; \xi \right) \in \Sigma \mathcal {F}^{{\mathbb {R}}}_{K, 0, 1}\left[ \epsilon _0, N \right]$ are functions in defined in Eqs. (4.5) and (4.60);
- ${\boldsymbol{A}}_{\frac{1}{2}}\left( U;x \right) \in \left( \Sigma \mathcal {F}^{{\mathbb {R}}}_{K, 0, 1}\left[ \epsilon _0, N \right] \right) ^{2\times 2}$ is defined as
  5.11 $$\begin{aligned} {\boldsymbol{A}}_{\frac{1}{2}} \left( U, x \right) \triangleq A_{\frac{1}{2}}\left( U;x \right) \ \begin{bmatrix} 1 &  1 \\ 1 &  1 \end{bmatrix} \triangleq \frac{1}{2} \frac{1}{\sqrt{2\gamma }} \left( \frac{ B^2_{\texttt{b}}\left( \textsf{M}U ; x \right) }{2} + \frac{{\texttt{b}}^2}{2} \texttt{f}\left( \eta ;x \right) - w_{\texttt{b}}\left( \textsf{M}U ; x \right) \right) \ \begin{bmatrix} 1 &  1 \\ 1 &  1 \end{bmatrix} , \end{aligned}$$
  where $w_{\texttt{b}}\left( \eta , \psi ; x \right)$ is defined in Eq. (4.3);
- ${\boldsymbol{A}}_{\left[ 0 \right] }\left( U;x, \xi \right)$ and ${\boldsymbol{R}}\left( U \right)$ are as in Notation 4.4.

*Proof.*

### Step 1

(Diagonalization of the linear part of (4.1)) We diagonalize the matrix defined in (2.15) as

5.12 $$\begin{aligned} {\boldsymbol{L}}_{\gamma , {\texttt{b}}}\left( \xi \right) = {\boldsymbol{\textsf{M}}}_{\gamma , {\texttt{b}}}\left( \xi \right) {\boldsymbol{D}}_{\gamma , {\texttt{b}}}\left( \xi \right) {\boldsymbol{\textsf{M}}}_{\gamma , {\texttt{b}}}^{-1}\left( \xi \right) ,\qquad {\boldsymbol{D}}_{\gamma , {\texttt{b}}}\left( \xi \right) \triangleq \textrm{i}\omega _{\gamma , {\texttt{b}}}\left( \xi \right) \begin{bmatrix} 1 &  0 \\ 0 &  -1 \end{bmatrix}\in \left( \tilde{\Gamma }^{\frac{3}{2}}_0 \right) ^{2\times 2} , \end{aligned}$$

where $\omega _{\gamma , {\texttt{b}}}\left( \xi \right) \in \tilde{\Gamma }^{\frac{3}{2}}_0$ is defined in Eq. (2.19). Defining *U* as in (5.8) where $$ \begin{bmatrix} \eta \\ \psi \end{bmatrix} $$ solves (2.14), the vector-valued complex function *U* solves the diagonal linear system

$$\begin{aligned} U_t = {\boldsymbol{D}}_{\gamma , {\texttt{b}}}\left( D \right) U . \end{aligned}$$

In particular thanks to the relation (5.8) combined with (5.5), which implies that $\textsf{m}_{\gamma , {\texttt{b}}}\left( \left| D \right| \right) \left[ 1 \right] =0$, assure us that if $\left( \eta , \psi \right) \in H^{s+\frac{1}{4}}_0\left( {\mathbb {T}};{\mathbb {R}} \right) \times {\dot{H}}^{s-\frac{1}{4}}\left( {\mathbb {T}};{\mathbb {R}} \right)$ then $u\in H^s_0\left( {\mathbb {T}};{\mathbb {C}} \right) \simeq {\dot{H}}^s\left( {\mathbb {T}};{\mathbb {C}} \right)$ for $s>0$.

Next, expanding (5.5) we have that

5.13 $$\begin{aligned} \textsf{m}_{\gamma , {\texttt{b}}}\left( \xi \right) = \frac{1}{\left( 2\gamma \left| \xi \right| \right) ^{\frac{1}{4}}} +\left( \frac{{\texttt{b}}}{2} \right) ^2 \frac{1}{\left( 2\gamma \left| \xi \right| \right) ^{\frac{5}{4}}} + \textsf{m}_{\gamma , {\texttt{b}}; -\frac{9}{4}}\left( \left| \xi \right| \right) , \end{aligned}$$

with $$ \textsf{m}_{\gamma , {\texttt{b}}; -\frac{9}{4}}\left( \left| \xi \right| \right) \in \tilde{\Gamma }^{-\frac{9}{4}}_0 $$.

### Notation 5.3

From now on we use the abbreviated notation $\textsf{M}\triangleq {\boldsymbol{\textsf{M}}}_{\gamma , {\texttt{b}}}\left( \left| D \right| \right)$ defined in (5.7) and $\textsf{m}\triangleq \textsf{m}_{\gamma , {\texttt{b}}}\left( \left| D \right| \right)$ defined in (5.8) for the sake of brevity.

### Step 2

(Reformulation of Eq. (4.1) in complex coordinates)

Recall the matrix of Fourier symbols ${\boldsymbol{L}}_{\gamma , {\texttt{b}}}$ in (2.14) and the matrix of symbols ${\boldsymbol{Q}}_{\gamma , {\texttt{b}}}$ in (4.2); we define that

5.14 $$\begin{aligned} \begin{aligned}{\boldsymbol{Q}} _{\gamma , {\texttt {b}}; \geqslant 1}\left( \eta , \psi ;x, \xi \right)&\triangleq {\boldsymbol{Q}}_{\gamma , {\texttt {b}}}\left( \eta , \psi ;x, \xi \right) - {\boldsymbol{L}}_{\gamma , {\texttt {b}}}\left( \xi \right) \\  &= \begin{bmatrix} 0&  0\\ \textsf {q}\left( \eta ,\psi ;x,\xi \right) &  0 \end{bmatrix} \in \Sigma \Gamma ^{2}_{K, 0, 1}\left[ \epsilon _0, N \right] , \end{aligned}\end{aligned}$$

where, from Eqs. (5.20) and (4.2), $\textsf{q}\left( U;x, \xi \right)$ is the real-valued symbol

5.15 $$\begin{aligned} \begin{aligned} \textsf{q}\left( \eta ,\psi ;x, \xi \right) =&\ \gamma \ \texttt{f}\left( \eta ;x \right) \left( \left| \xi \right| ^2-1 \right) - w_{\texttt{b}}\left( \eta , \psi ;x \right) \left| \xi \right| + {\texttt{b}}^2\left( \frac{1}{\left( 1+2\eta \right) }-1 \right) \\ =&\ \gamma \ \texttt{f}\left( \eta ;x \right) \left| \xi \right| ^2 - w_{\texttt{b}}\left( \eta , \psi ;x \right) \left| \xi \right| + \textsf{q}_0\left( \eta ,\psi ;x, \xi \right) , \end{aligned} \end{aligned}$$

with

$$\begin{aligned} \textsf{q}_0\left( \eta ,\psi ;x, \xi \right) \triangleq - \gamma \ \texttt{f}\left( \eta ;x \right) + {\texttt{b}}^2\left( \frac{1}{1+2\eta }-1 \right) \in \Sigma \Gamma ^0_{K, 0, 1}\left[ \epsilon _0, N \right] . \end{aligned}$$

Then, by (5.12), Eq. (4.1) becomes

5.16 $$\begin{aligned} \begin{aligned}&\begin{bmatrix} \eta \\ \psi \end{bmatrix} _t -\textsf{M}{\boldsymbol{D}}_{\gamma , {\texttt{b}}}\left( D \right) \textsf{M}^{-1} \begin{bmatrix} \eta \\ \psi \end{bmatrix} \\&= {{{\,\mathrm{Op^{BW}}\,}}\left( {\boldsymbol{Q}}_{\ \gamma , {\texttt{b}}; \geqslant 1}\left( \eta ,\psi ; x, \xi \right) + {\boldsymbol{B}}_{\texttt{b}}\left( \eta ,\psi ; x \right) \left| \xi \right| - \textrm{i}V_{{\texttt{b}}} \left( \eta , \psi ;x \right) \ \text {Id}_{{\mathbb {R}}^2}\ \xi + {\boldsymbol{A}}_{\left[ 0 \right] }\left( \eta ,\psi ; x, \xi \right) \right) } \begin{bmatrix} \eta \\ \psi \end{bmatrix} \\&\quad +{{{\,\mathrm{Op^{BW}}\,}}\left( {\boldsymbol{A}}_{\left[ 0 \right] }\left( \eta ,\psi ; x, \xi \right) \right) } + {\boldsymbol{R}}\left( \eta , \psi \right) \begin{bmatrix} \eta \\ \psi \end{bmatrix} . \end{aligned} \end{aligned}$$

Since $$ U=\textsf{M}^{-1} \begin{bmatrix} \eta \\ \psi \end{bmatrix} $$ we can derive the evolution equation for *U* multiplying (5.16) from the left for $\textsf{M}^{-1}$ obtaining

5.17 $$\begin{aligned} \begin{aligned}&U_t - {\boldsymbol{D}}_{\gamma , {\texttt{b}}}\left( D \right) U \\&= \textsf{M}^{-1} {{{\,\mathrm{Op^{BW}}\,}}\left( {\boldsymbol{Q}}_{\ \gamma , {\texttt{b}}; \geqslant 1}\left( \textsf{M}U; x, \xi \right) + {\boldsymbol{B}}_{\texttt{b}}\left( \textsf{M}U; x \right) \left| \xi \right| - \textrm{i}V_{{\texttt{b}}} \left( \textsf{M}U ;x \right) \ \text {Id}_{{\mathbb {R}}^2}\ \xi \right) }\textsf{M}U \\&\quad +\textsf{M}^{-1} {{{\,\mathrm{Op^{BW}}\,}}\left( {\boldsymbol{A}} _{\left[ 0 \right] }\left( \textsf{M}U; x, \xi \right) \right) }\textsf{M}U +\textsf{M}^{-1} {\boldsymbol{R}}\left( \textsf{M}U \right) \textsf{M}U. \end{aligned} \end{aligned}$$

Recall that $\textsf{M}^{-1}$ is explicitly defined in (5.7). We have the following relation:

5.18 $$\begin{aligned}&\textsf{M}^{-1} \begin{bmatrix} A_1 &  A_2 \\ A_3 &  A_4 \end{bmatrix} \textsf{M}\nonumber \\&\quad = \frac{1}{2} \begin{bmatrix} \textsf{m}^{-1} A_1 \textsf{m} + \textsf{m} A_4 \textsf{m}^{-1} + \textrm{i}\textsf{m} A_3 \textsf{m} - \textrm{i}\textsf{m}^{-1} A_2 \textsf{m}^{-1} &  \textsf{m}^{-1} A_1 \textsf{m} - \textsf{m} A_4 \textsf{m}^{-1} + \textrm{i}\textsf{m} A_3 \textsf{m} + \textrm{i}\textsf{m}^{-1} A_2 \textsf{m}^{-1} \\ \textsf{m}^{-1} A_1 \textsf{m} - \textsf{m} A_4 \textsf{m}^{-1} - \textrm{i}\textsf{m} A_3 \textsf{m} - \textrm{i}\textsf{m}^{-1} A_2 \textsf{m}^{-1} &  \textsf{m}^{-1} A_1 \textsf{m} + \textsf{m} A_4 \textsf{m}^{-1} - \textrm{i}\textsf{m} A_3 \textsf{m} + \textrm{i}\textsf{m}^{-1} A_2 \textsf{m}^{-1} \end{bmatrix}. \end{aligned}$$

Notice now that given

$$\begin{aligned} a \left( U;x,\xi \right) \in \Sigma \Gamma ^{m_a}_{K, 0, 1}\left[ \epsilon _0, N \right] ,  &   \alpha \left( \xi \right) \in \tilde{\Gamma }^{m_\alpha }_0,  &   \beta \left( \xi \right) \in \tilde{\Gamma }^{m_\beta }_0, \end{aligned}$$

applying Proposition 3.15 we have that

5.19 $$\begin{aligned} \begin{aligned}&\alpha \left( D \right) {{{\,\mathrm{Op^{BW}}\,}}\left( a\left( U;x,\xi \right) \right) }\beta \left( D \right) \\&\quad = {{{\,\mathrm{Op^{BW}}\,}}\left( a\left( U;x, \xi \right) \alpha \left( \xi \right) \beta \left( \xi \right) + \frac{1}{2\textrm{i}} \ \left( a\left( U;x, \xi \right) \right) _x \left( \alpha _\xi \left( \xi \right) \beta \left( \xi \right) - \alpha \left( \xi \right) \beta _\xi \left( \xi \right) \right) \right) } \\&\qquad +{{{\,\mathrm{Op^{BW}}\,}}\left( \Sigma \Gamma ^{m_a+m_\alpha +m_\beta -2}_{K, 0, 1}\left[ \epsilon _0, N \right] \right) } \\&\qquad + \Sigma \mathcal {R}^{-\varrho }_{K, 0, 1}\left[ \epsilon _0, N \right] . \end{aligned} \end{aligned}$$

Then, applying (5.18), we have that

5.20 $$\begin{aligned} \textsf{M}^{-1} {{{\,\mathrm{Op^{BW}}\,}}\left( {\boldsymbol{Q}}_{\ \gamma , {\texttt{b}}; \geqslant 1}\left( \textsf{M}U; x, \xi \right) \right) }\textsf{M}= \frac{\textrm{i}}{2} \ \textsf{m} \ {{{\,\mathrm{Op^{BW}}\,}}\left( \textsf{q}\left( U;x,\xi \right) \right) } \textsf{m} \ \begin{bmatrix} 1 &  1 \\ - 1 &  - 1 \end{bmatrix} , \end{aligned}$$

where $\textsf{q}\left( U;x,\xi \right)$ is the real valued symbol in (5.15) evaluated at $(\eta ,\psi )=\textsf{M}U$. We apply Equation (5.19) and obtain that

5.21 $$\begin{aligned} \textsf{m} \ {{{\,\mathrm{Op^{BW}}\,}}\left( \textsf{q}\left( U;x,\xi \right) \right) } \textsf{m} ={{{\,\mathrm{Op^{BW}}\,}}\left( \textsf{q}\left( U;x, \xi \right) \textsf{m}_{\gamma , {\texttt{b}}}^2\left( \xi \right) + A_{\left[ -\frac{1}{2} \right] }\left( U;x, \xi \right) \right) } + R\left( U \right) . \end{aligned}$$

Thus, combining Eqs (5.20) and (5.21), we obtain

5.22 $$\begin{aligned} \begin{aligned}&\textsf{M}^{-1} {{{\,\mathrm{Op^{BW}}\,}}\left( {\boldsymbol{Q}}_{\ \gamma , {\texttt{b}}; \geqslant 1}\left( \textsf{M}U; x, \xi \right) \right) }\textsf{M}\\&= \frac{\textrm{i}}{2} \ \left( {{{\,\mathrm{Op^{BW}}\,}}\left( \textsf{q}\left( U;x, \xi \right) \textsf{m}_{\gamma , {\texttt{b}}}^2\left( \xi \right) + A_{\left[ -\frac{1}{2} \right] }\left( U;x, \xi \right) \right) } + R\left( U \right) \right) \begin{bmatrix} 1 &  1 \\ - 1 &  - 1 \end{bmatrix} . \end{aligned} \end{aligned}$$

Next we apply Eqs. (5.18) and (5.19) to the matrix-valued symbol ${\boldsymbol{B}}_{\texttt{b}}\left( \textsf{M}U; x \right) \left| \xi \right|$ defined in (4.4) and obtain that

5.23 $$\begin{aligned} \begin{aligned}&\textsf{M}^{-1} {{{\,\mathrm{Op^{BW}}\,}}\left( {\boldsymbol{B}}_{\texttt{b}}\left( \textsf{M}U ; x \right) \left| \xi \right| \right) } \textsf{M}\\&= \frac{1}{2}{{{\,\mathrm{Op^{BW}}\,}}\left( B_{\texttt{b}}\left( \textsf{M}U ; x \right) \left| \xi \right| \right) } \begin{bmatrix} 0 &  1 \\ 1 &  0 \end{bmatrix} + \frac{\textrm{i}}{4} {{{\,\mathrm{Op^{BW}}\,}}\left( B^2_{\texttt{b}}\left( \textsf{M}U ; x \right) \left| \xi \right| \textsf{m}_{\gamma , {\texttt{b}}}^2 \left( \xi \right) \right) } \begin{bmatrix} 1 &  1 \\ -1 &  -1 \end{bmatrix} \\&\quad + {{{\,\mathrm{Op^{BW}}\,}}\left( {\boldsymbol{A}}_{\left[ 0 \right] }\left( U;x, \xi \right) \right) } + {\boldsymbol{R}}\left( U \right) . \end{aligned} \end{aligned}$$

The same procedure give s us the conjugations

5.24 $$\begin{aligned} \begin{aligned} \textsf{M}^{-1} {{{\,\mathrm{Op^{BW}}\,}}\left( \textrm{i}V_{{\texttt{b}}} \left( \textsf{M}U;x \right) \xi \right) }\text {Id}_{{\mathbb {R}}^2} \textsf{M}=&\ {{{\,\mathrm{Op^{BW}}\,}}\left( \textrm{i}V_{{\texttt{b}}} \left( \textsf{M}U;x \right) \xi \right) }\text {Id}_{{\mathbb {C}}^2} \\&+ {{{\,\mathrm{Op^{BW}}\,}}\left( {\boldsymbol{A}}_{\left[ 0 \right] }\left( U;x, \xi \right) \right) } + {\boldsymbol{R}}\left( U \right) , \\ \textsf{M}^{-1}{{{\,\mathrm{Op^{BW}}\,}}\left( {\boldsymbol{A}}_{\left[ 0 \right] }\left( \textsf{M}U ; x, \xi \right) \right) } \textsf{M}=&\ {{{\,\mathrm{Op^{BW}}\,}}\left( {\boldsymbol{A}}_{\left[ 0 \right] }\left( U;x, \xi \right) \right) } + {\boldsymbol{R}}\left( U \right) , \end{aligned} \end{aligned}$$

while Proposition 3.17 implies that

5.25 $$\begin{aligned} \textsf{M}^{-1}{\boldsymbol{R}}\left( \textsf{M}U \right) \textsf{M}\leadsto {\boldsymbol{R}}\left( U \right) . \end{aligned}$$

We plug Equations (5.22) to (5.25) in Equation (5.17) and obtain that

5.26 $$\begin{aligned} U_t-{\boldsymbol{D}}_{\gamma , {\texttt{b}}}\left( D \right) U = {{{\,\mathrm{Op^{BW}}\,}}\left( \sum _{\textsf{j}=0}^2 {\boldsymbol{A}}_{\frac{3-\textsf{j}}{2}}^{\text {NH}}\left( U;x,\xi \right) + {\boldsymbol{A}}_{\left[ 0 \right] }\left( U;x, \xi \right) \right) } U +{\boldsymbol{R}}\left( U \right) U, \end{aligned}$$

with

$$\begin{aligned} {\boldsymbol{A}}_{\frac{3-\textsf{j}}{2}}^{\text {NH}}\left( U;x,\xi \right) \in \left( \Sigma \Gamma ^{\frac{3-\textsf{j}}{2}}_{K, 0, 1}\left[ \epsilon _0, N \right] \right) ^{2\times 2}, \end{aligned}$$

and are explicitly defined as

5.27 $$\begin{aligned} \begin{aligned} {\boldsymbol{A}}_{\frac{3}{2}}^{\text {NH}}\left( U;x,\xi \right) \triangleq&\ \frac{\textrm{i}}{2} \ \textsf{q}\left( U;x, \xi \right) \textsf{m}_{\gamma , {\texttt{b}}}^2\left( \xi \right) \begin{bmatrix} 1 &  1 \\ - 1 &  - 1 \end{bmatrix}, \\ {\boldsymbol{A}}_{1}^{\text {NH}}\left( U;x,\xi \right) \triangleq&- \textrm{i}V_{{\texttt{b}}}\left( \textsf{M}U;x \right) \xi \begin{bmatrix} 1& 0\\ 0& 1 \end{bmatrix} + \frac{B_{\texttt{b}}\left( \textsf{M}U;x \right) }{2} \ \left| \xi \right| \begin{bmatrix} 0& 1\\ 1& 0 \end{bmatrix}, \\ {\boldsymbol{A}}_{\frac{1}{2}}^{\text {NH}}\left( U;x,\xi \right) \triangleq&\frac{\textrm{i}}{4} \ B^2_{\texttt{b}}\left( \textsf{M}U ; x \right) \left| \xi \right| \textsf{m}_{\gamma , {\texttt{b}}}^2 \left( \xi \right) \begin{bmatrix} 1 &  1 \\ - 1 &  - 1 \end{bmatrix}. \end{aligned} \end{aligned}$$

We now expand the symbols in (5.27) in decreasing para-differential orders using Eqs. (5.15) and (5.13), thus obtaining that

5.28 $$\begin{aligned} \begin{aligned} \textsf{q}\left( U;x, \xi \right) \textsf{m}_{\gamma , {\texttt{b}}}^2\left( \xi \right) =&\ \sqrt{\frac{\gamma }{2}} \ \texttt{f} \left( \eta ;x \right) \left| \xi \right| ^{\frac{3}{2}} \\&+ \frac{1}{\sqrt{2\gamma }}\left( \left( \frac{{\texttt{b}}}{2} \right) ^2 \texttt{f}\left( \eta ;x \right) - w_{\texttt{b}}\left( \textsf{M}U ; x \right) \right) \left| \xi \right| ^{\frac{1}{2}} \\&\quad + A_{\left[ -\frac{1}{2} \right] }\left( U;x, \xi \right) , \\ B^2_{\texttt{b}}\left( \textsf{M}U ; x \right) \left| \xi \right| \textsf{m}_{\gamma , {\texttt{b}}}^2\left( \xi \right) =&\ \frac{1}{\sqrt{2\gamma }} \ B^2_{\texttt{b}}\left( \textsf{M}U ; x \right) \left| \xi \right| ^{\frac{1}{2}} + A_{\left[ -\frac{1}{2} \right] }\left( U;x, \xi \right) . \end{aligned} \end{aligned}$$

Thus, inserting (5.28) in (5.27), we obtain that

$$\begin{aligned} \sum _{\textsf{j}=0}^2 {\boldsymbol{A}}_{\frac{3-\textsf{j}}{2}}^{\text {NH}}\left( U;x,\xi \right) = \sum _{\textsf{j}=0}^2 {\boldsymbol{A}}_{\frac{3-\textsf{j}}{2}}^{\text {H}}\left( U;x,\xi \right) +{\boldsymbol{A}}_{\left[ -\frac{1}{2} \right] } \left( U;x, \xi \right) , \end{aligned}$$

where

$$\begin{aligned} \begin{aligned} {\boldsymbol{A}}_{\frac{3}{2}}^{\text {H}}\left( U;x,\xi \right) \triangleq&\ \frac{\textrm{i}}{2} \ \sqrt{\frac{\gamma }{2}} \ \texttt{f} \left( \eta ;x \right) \left| \xi \right| ^{\frac{3}{2}} \begin{bmatrix} 1 &  1 \\ - 1 &  - 1 \end{bmatrix}, \\ {\boldsymbol{A}}_{1}^{\text {H}}\left( U;x,\xi \right) \triangleq&\ {\boldsymbol{A}}_{1}^{\text {NH}}\left( U;x,\xi \right) , \\ {\boldsymbol{A}}_{\frac{1}{2}}^{\text {H}}\left( U;x,\xi \right) \triangleq&\ \frac{\textrm{i}}{2} \frac{1}{\sqrt{2\gamma }} \left( \frac{ B^2_{\texttt{b}}\left( \textsf{M}U ; x \right) }{2} + \left( \frac{{\texttt{b}}}{2} \right) ^2 \texttt{f}\left( \eta ;x \right) - w_{\texttt{b}}\left( \textsf{M}U ; x \right) \right) \left| \xi \right| ^{\frac{1}{2}} \ \begin{bmatrix} 1 &  1 \\ - 1 &  - 1 \end{bmatrix}. \end{aligned} \end{aligned}$$

Therefore, (5.26) becomes

5.29 $$\begin{aligned} U_t-{\boldsymbol{D}}_{\gamma , {\texttt{b}}}\left( D \right) U = {{{\,\mathrm{Op^{BW}}\,}}\left( \sum _{\textsf{j}=0}^2 {\boldsymbol{A}}_{\frac{3-\textsf{j}}{2}}^{\text {H}}\left( U;x,\xi \right) + {\boldsymbol{A}}_{\left[ 0 \right] }\left( U;x, \xi \right) \right) } U +{\boldsymbol{R}}\left( U \right) U. \end{aligned}$$

We now further refine the expression deduced in (5.29) by expressing the leading term as a quasilinear perturbation of the unperturbed frequencies $\omega _{\gamma , {\texttt{b}}}\left( \xi \right)$, cf. (2.19). Using (2.20) we have that

$$\begin{aligned} {\boldsymbol{A}}^{\text {H}}_{\frac{3}{2}}\left( U;x, \xi \right) = \textrm{i}\ \left( \frac{\texttt{f}\left( \eta ;x \right) }{2} \omega _{\gamma , {\texttt{b}}}\left( \xi \right) + \frac{1}{\sqrt{2\gamma }} \frac{\texttt{f}\left( \eta ;x \right) }{2} \left( \frac{{\texttt{b}}}{2} \right) ^2 \left| \xi \right| ^{\frac{1}{2}} + {\boldsymbol{A}}_{\left[ -\frac{1}{2} \right] }\left( U;x, \xi \right) \right) \begin{bmatrix} 1 &  1 \\ - 1 &  - 1 \end{bmatrix} , \end{aligned}$$

so that, defining

$$\begin{aligned} {\boldsymbol{J}}_{\mathbb {C}} {\boldsymbol{A}}_{\frac{3}{2};\geqslant 1}\left( U, x \right) \triangleq&\ \frac{\texttt{f}\left( \eta ;x \right) }{2} \begin{bmatrix} 1 &  1 \\ - 1 &  - 1 \end{bmatrix} \ \textrm{i}\ , \\ {\boldsymbol{J}}_{\mathbb {C}} {\boldsymbol{A}}_{\frac{1}{2}} \left( U, x \right) \triangleq&\ \frac{1}{2} \frac{1}{\sqrt{2\gamma }} \left( \frac{ B^2_{\texttt{b}}\left( \textsf{M}U ; x \right) }{2} + 2\left( \frac{{\texttt{b}}}{2} \right) ^2 \texttt{f}\left( \eta ;x \right) - w_{\texttt{b}}\left( \textsf{M}U ; x \right) \right) \ \begin{bmatrix} 1 &  1 \\ - 1 &  - 1 \end{bmatrix} \ \textrm{i},\\ {\boldsymbol{J}}_{\mathbb {C}} {\boldsymbol{A}}_{1} \left( U;x, \xi \right) \triangleq&{\boldsymbol{A}}^{\text {H}}_{1}\left( U;x, \xi \right) , \end{aligned}$$

we transform (5.29) into

5.30 $$\begin{aligned} \begin{aligned}&U_t -{\boldsymbol{D}}_{\gamma , {\texttt{b}}}\left( D \right) U \\&= {\boldsymbol{J}}_{\mathbb {C}} \ {{{\,\mathrm{Op^{BW}}\,}}\left( {\boldsymbol{A}}_{\frac{3}{2};\geqslant 1}\left( U;x \right) \ \omega _{\gamma , {\texttt{b}}}\left( \xi \right) + {\boldsymbol{A}}_{1}\left( U;x, \xi \right) + {\boldsymbol{A}}_{\frac{1}{2}}\left( U;x \right) \ \left| \xi \right| ^{\frac{1}{2}} + {\boldsymbol{A}}_{\left[ 0 \right] }\left( U;x, \xi \right) \right) } U \\&\quad +{\boldsymbol{R}}\left( U \right) U . \end{aligned} \end{aligned}$$

Next, an explicit computation shows that

$$\begin{aligned} {\boldsymbol{D}}_{\gamma , {\texttt{b}}}\left( \xi \right) = \omega _{\gamma , {\texttt{b}}}\left( \xi \right) \ {\boldsymbol{J}}_{\mathbb {C}} \begin{bmatrix} 0& 1\\ 1& 0 \end{bmatrix} . \end{aligned}$$

Thus, defining

$$\begin{aligned} {\boldsymbol{A}}_{\frac{3}{2}}\left( U;x \right) \triangleq \begin{bmatrix} 0& 1\\ 1& 0 \end{bmatrix} + {\boldsymbol{A}}_{\frac{3}{2};\geqslant 1}\left( U, x \right) , \end{aligned}$$

it remains only to show that (5.30) is a complex Hamiltonian system and that ${\boldsymbol{J}}_\mathbb {C}{\boldsymbol{A}}_{\left[ 0 \right] }\left( U;x, \xi \right)$ is linearly symplectic. Indeed complex variables transformation ${\boldsymbol{\textsf{M}}}_{\gamma , {\texttt{b}}}^{-1}$ in (5.8) is the composition of the complex transformation $\mathcal {C}^{-1}$ in (5.1) and the real symplectic map ${\boldsymbol{S}}_{\gamma ,{\texttt{b}}}^{-1}(D)$, see (5.6). Then the equation for *U* has the complex Hamiltonian form in (5.3). Then we apply Lemma A.14 to each homogeneous component in (5.30). As the explicit positive order symbols are linearly symplectic by direct inspections, we eventually replace ${\boldsymbol{A}}_{\left[ 0 \right] }\left( U;x, \xi \right)$ as in (A.17) to get a linearly symplectic matrix of symbols. This conclude the proof.

$$\square $$

## Block Diagonalization and Reduction to Constant Coefficients

In this section, we perform spectrally localized, linearly symplectic, and bounded transformations to conjugate the complex Kelvin-Helmholtz system (5.9) into a diagonal constant-coefficient system, up to a smoothing remainder. Specifically, we prove the following.

### Proposition 6.1

Let $N\in {\mathbb {N}}$ and $\varrho > 3 \left( N+1 \right)$, there exists $\underline{K'} > 0$ such that for any $K > \underline{K'}$ there are $s_0 > 0$, $\epsilon _0 > 0$ such that, for any solution $U\in B^{K}_{s_0}\left( I;\epsilon _0 \right)$ solution of (5.9) there exists a real-to-real invertible matrix of spectrally localized maps ${\boldsymbol{B}}\left( U;t \right)$ such that

1. ${\boldsymbol{B}}\left( U;t \right) , \ {\boldsymbol{B}}\left( U;t \right) ^{-1} \in \left( \mathcal {S}^0_{K, \underline{K'}-1, 0}\left[ \epsilon _0, N \right] \right) ^{2\times 2}$ are linearly symplectic up to homogeneity *N*, according to Definition A.4. Moreover, ${\boldsymbol{B}}\left( U;t \right) -\text {Id}\in \left( \Sigma \mathcal {S}^{\frac{3}{2}\left( N+1 \right) }_{K, \underline{K'}-1, 1}\left[ \epsilon _0, N \right] \right) ^{2\times 2};$
2. The variable $W\triangleq {\boldsymbol{B}}\left( U;t \right) U$ solves 6.1 where
  $\omega _{\gamma , {\texttt{b}}}\left( \xi \right) \in \tilde{\Gamma }^{\frac{3}{2}}_0$ is defined in (2.19);
  and is independent of *x*;
  and is independent of *x*;
  and is independent of *x*;
  , is independent of *x* and such that ;
  $$\pmb {\textsf{R}}\left( U;t \right) \in \left( \Sigma \mathcal {R}^{-\varrho + 3\left( N+1 \right) }_{K, \underline{K'}, 1}\left[ \epsilon _0, N \right] \right) ^{2\times 2} $$.

The rest of the section is devoted to the proof of Proposition 6.1. The procedure is now rather classical so we state the main steps, for the missing details, we refer the interested reader to [20, Section 6].

### A Complex Alinhac Good Unknown

We first perform a change of variable, known as *Alinhac good unknown* that cancels the anti-diagonal terms of order one in (5.9).

#### Lemma 6.2

(Alinhac Good Unknown for the Kelvin-Helmholtz equations in complex coordinates) Let $N\in {\mathbb {N}}$, $\gamma > 0$, ${\texttt{b}}\in {\mathbb {R}}$ and $\varrho {\geqslant }0$, for any $K\in {\mathbb {N}}$ there exist $s_0 >0$ and $\epsilon _0 > 0$ such that if $U\in B^K_{s_0}\left( I;\epsilon _0 \right)$ solves (5.9) there exists a real-to-real matrix of operators $\textbf{G}(U)$ such that

1. ${\boldsymbol{G}}\left( U \right) , \ {\boldsymbol{G}}\left( U \right) ^{-1} \in \left( \mathcal {S}^0_{K,0, 0}\left[ \epsilon _0, N \right] \right) ^{2\times 2}$ are linearly symplectic, according to Definition A.3. Moreover, ${\boldsymbol{G}}\left( U \right) -\text {Id}\in \left( \Sigma \mathcal {S}^{-\frac{1}{2}}_{K, 0, 1}\left[ \epsilon _0, N \right] \right) ^{2\times 2}$;
2. The variable $U_0\triangleq {\boldsymbol{G}}\left( U \right) U$ solves
  6.2 $$\begin{aligned} \begin{aligned} \partial _t U_0&= {\boldsymbol{J}}_{\mathbb {C}} \ {{{\,\mathrm{Op^{BW}}\,}}\left( {\boldsymbol{A}}_{\frac{3}{2}}^{(0)}\left( U;x \right) \ \omega _{\gamma , {\texttt{b}}}\left( \xi \right) + {\boldsymbol{A}}_{\frac{1}{2}}^{(0)} \left( U;x \right) \ \left| \xi \right| ^{\frac{1}{2}} + {\boldsymbol{A}}_{\left[ 0;1 \right] }\left( U;x, \xi \right) \right) } U_0 \\&\quad - {{\,\mathrm{Op^{BW}_{vec}}\,}}\left( \textrm{i}V_{\texttt{b}}\left( \textsf{M}U;x \right) \xi \right) U_0 + {\boldsymbol{R}}\left( U \right) U_0 , \end{aligned} \end{aligned}$$
  where
  the symbols ${\boldsymbol{A}}_{\frac{3}{2}}^{(0)} \triangleq {\boldsymbol{A}}_{\frac{3}{2}}, \ \omega _{\gamma , {\texttt{b}}}$ and $V_{\texttt{b}}$ are the same as in the statement of Proposition 5.2 and ${\boldsymbol{A}}_{\left[ 0;1 \right] }$, ${\boldsymbol{R}}$ are as in Notation 5.1;
  ${\boldsymbol{A}}^{(0)}_{\frac{1}{2}} \in \left( \Sigma \mathcal {F}^{{\mathbb {R}}}_{K, 0, 1}\left[ \epsilon _0, N \right] \right) ^{2\times 2}$ is defined as
    $$\begin{aligned} {\boldsymbol{A}}_{\frac{1}{2}}^{\left( 0 \right) } \left( U; x \right) \triangleq A_{\frac{1}{2}}^{\left( 0 \right) }\left( U;x \right) \ \begin{bmatrix} 1 &  1 \\ 1 &  1 \end{bmatrix} \triangleq \frac{1}{2} \frac{1}{\sqrt{2\gamma }} \left( \frac{{\texttt{b}}^2}{2} \texttt{f}\left( \eta ;x \right) - w_{\texttt{b}}\left( \textsf{M}U ; x \right) \right) \ \begin{bmatrix} 1 &  1 \\ 1 &  1 \end{bmatrix} , \end{aligned}$$
    where $w_{\texttt{b}}\left( \eta , \psi ; x \right)$ is defined in Eq. (4.3).

#### Proof

The procedure that we use here is the same as the one proved in [20, Section 6.1], thus we omit to perform detailed computations, in particular the Item 1 is true by direct computations and the fact that ${\boldsymbol{G}}\left( U \right) -\text {Id}\in \left( \Sigma \mathcal {S}^0_{K,0,1}\left[ \epsilon _0, N \right] \right) ^{2\times 2}$. This latter fact is a consequence of Lemma 3.7. The only difference, compared to [20], is the symbol ${\boldsymbol{A}}_{\frac{1}{2}}\left( U;x \right) \left| \xi \right| ^{\frac{1}{2}} \in \Sigma \Gamma ^{\frac{1}{2}}_{K, 0, 1}\left[ \epsilon _0, N \right]$ appearing in (5.9) and defined explicitly in (5.11), which shall be treated in detail. Let us define the transformation

6.3 $$\begin{aligned} {\boldsymbol{G}}\left( U \right) \triangleq \text {Id}- \frac{\textrm{i}}{2} \begin{bmatrix} 1& 1\\ -1& -1 \end{bmatrix} {{{\,\mathrm{Op^{BW}}\,}}\left( B_{\texttt{b}}\left( \textsf{M}U;x \right) \textsf{m}_{\gamma , {\texttt{b}}}^2 \left( {\xi } \right) \right) }. \end{aligned}$$

The fact that ${\boldsymbol{G}}\left( U \right)$ is a nilpotent perturbation of the identity allows us to compute its inverse as

$$\begin{aligned} {\boldsymbol{G}}\left( U \right) ^{-1}\triangleq \text {Id}+ \frac{\textrm{i}}{2} \begin{bmatrix} 1& 1\\ -1& -1 \end{bmatrix} {{{\,\mathrm{Op^{BW}}\,}}\left( B_{\texttt{b}}\left( \textsf{M}U;x \right) \textsf{m}_{\gamma , {\texttt{b}}}^2 \left( {\xi } \right) \right) }. \end{aligned}$$

Thus, defining $U_0 \triangleq {\boldsymbol{G}}\left( U \right) U$, and using the conjugation rules

6.4 $$\begin{aligned} \begin{aligned}&{\boldsymbol{G}}\left( U \right) {\boldsymbol{J}}_{\mathbb {C}} {{{\,\mathrm{Op^{BW}}\,}}\left( {\boldsymbol{A}}_{\frac{3}{2}}\omega _{\gamma ,{\texttt{b}}}\left( \xi \right) \right) }{\boldsymbol{G}}\left( U \right) ^{-1}\\&\quad = {\boldsymbol{J}}_{\mathbb {C}} \\&{{{\,\mathrm{Op^{BW}}\,}}\left( {\boldsymbol{A}}_{\frac{3}{2}}\omega _{\gamma ,{\texttt{b}}}\left( \xi \right) - \textrm{i}\frac{B_{\texttt{b}}}{2}\left| \xi \right| \begin{bmatrix} 1& 0\\ 0& -1 \end{bmatrix} +\frac{B^2_{\texttt{b}}}{4}\ \textsf{m}^2_{\gamma , {\texttt{b}}}\left( \xi \right) \begin{bmatrix} 1& 1\\ 1& 1 \end{bmatrix} +{\boldsymbol{A}}_{\left[ 0 \right] } \right) } +{\boldsymbol{R}}\left( U \right) , \\&{\boldsymbol{G}}\left( U \right) {{\,\mathrm{Op^{BW}_{vec}}\,}}\left( -\textrm{i}V_{\texttt{b}}\xi \right) \text {Id}_{{\mathbb {C}}^2} {\boldsymbol{G}}\left( U \right) ^{-1} = {{\,\mathrm{Op^{BW}_{vec}}\,}}\left( -\textrm{i}V_{\texttt{b}}\xi \right) \text {Id}_{{\mathbb {C}}^2}, \\&{\boldsymbol{G}}\left( U \right) {\boldsymbol{J}}_{\mathbb {C}} {{{\,\mathrm{Op^{BW}}\,}}\left( \textrm{i}\frac{B_{\texttt{b}}}{2}\left| \xi \right| \begin{bmatrix} 1& 0\\ 0& -1 \end{bmatrix} \right) } {\boldsymbol{G}}\left( U \right) ^{-1}\\&\quad = {\boldsymbol{J}}_{\mathbb {C}} {{{\,\mathrm{Op^{BW}}\,}}\left( \textrm{i}\frac{B_{\texttt{b}}}{2}\left| \xi \right| \begin{bmatrix} 1& 0\\ 0& -1 \end{bmatrix} -\frac{B^2_{\texttt{b}}}{2}\ \textsf{m}^2_{\gamma , {\texttt{b}}}\left( \xi \right) \begin{bmatrix} 1& 1\\ 1& 1 \end{bmatrix} +{\boldsymbol{A}}_{\left[ 0 \right] } \right) } + {\boldsymbol{R}}\left( U \right) , \end{aligned} \end{aligned}$$

we get, using also Eqs. (6.4) and (5.13),

6.5 $$\begin{aligned} \begin{aligned}&{\boldsymbol{G}}\left( U \right) {\boldsymbol{J}}_{\mathbb {C}}{{{\,\mathrm{Op^{BW}}\,}}\left( {\boldsymbol{A}}_{\frac{3}{2}}\omega _{\gamma ,{\texttt{b}}}\left( \xi \right) + {\boldsymbol{A}}_1\right) } {\boldsymbol{G}}\left( U \right) ^{-1} \\&\quad = {\boldsymbol{J}}_{\mathbb {C}} {{{\,\mathrm{Op^{BW}}\,}}\left( {\boldsymbol{A}}_{\frac{3}{2}}\omega _{\gamma ,{\texttt{b}}}\left( \xi \right) -\frac{1}{\sqrt{2\gamma }}\frac{B^2_{\texttt{b}}}{4} \ \left| \xi \right| ^{\frac{1}{2}} \begin{bmatrix} 1& 1\\ 1& 1 \end{bmatrix} + {\boldsymbol{A}}_{\left[ 0 \right] } \right) }\\&\quad + {{\,\mathrm{Op^{BW}_{vec}}\,}}\left( -\textrm{i}V_{\texttt{b}}\xi \right) +{\boldsymbol{R}}\left( U \right) . \end{aligned} \end{aligned}$$

Due to the particular nilpotent structure of ${\boldsymbol{J}}_{\mathbb {C}}{{{\,\mathrm{Op^{BW}}\,}}\left( {\boldsymbol{A}}_{\frac{1}{2}}\left( U;x \right) \left| \xi \right| ^{\frac{1}{2}}\right) }$ and ${\boldsymbol{G}(U)}-\text {Id}$, the conjugation for the symbol of order 1/2 explicitly given by

6.6 $$\begin{aligned} {\boldsymbol{G}}\left( U \right) {\boldsymbol{J}}_{\mathbb {C}}{{{\,\mathrm{Op^{BW}}\,}}\left( {\boldsymbol{A}}_{\frac{1}{2}}\left( U;x \right) \left| \xi \right| ^{\frac{1}{2}}\right) } {\boldsymbol{G}}\left( U \right) ^{-1} = {\boldsymbol{J}}_{\mathbb {C}}{{{\,\mathrm{Op^{BW}}\,}}\left( {\boldsymbol{A}}_{\frac{1}{2}}\left( U;x \right) \left| \xi \right| ^{\frac{1}{2}}\right) } . \end{aligned}$$

Remark that there is a cancellation of the terms of the form $|\xi |^{\frac{1}{2}}$ between (5.11) and (6.5) leading to the expression of ${\boldsymbol{A}}^{(0)}_{\frac{1}{2}}$ in the statement. Moreover, we have

6.7 $$\begin{aligned} {\boldsymbol{G}}\left( U \right) U_t = \partial _t U_0 - \left( \partial _t {\boldsymbol{G}}\left( U \right) \right) {\boldsymbol{G}}\left( U \right) ^{-1} U_0. \end{aligned}$$

Hence, from Item 1 and Proposition 3.15, we have that

6.8 $$\begin{aligned}&\left( \partial _t {\boldsymbol{G}}\left( U \right) \right) {\boldsymbol{G}}\left( U \right) ^{-1} = {{{\,\mathrm{Op^{BW}}\,}}\left( {\boldsymbol{A}}_{\left[ -\frac{1}{2};1 \right] }\left( U;x, \xi \right) \right) }, \quad \nonumber \\&{\boldsymbol{A}}_{\left[ -\frac{1}{2};1 \right] }\triangleq - \frac{\textrm{i}}{2} \begin{bmatrix} 1& 1\\ -1& -1 \end{bmatrix} {\partial _t B_{\texttt{b}}\left( \textsf{M}U;x \right) \textsf{m}_{\gamma , {\texttt{b}}}^2 \left( {\xi } \right) } . \end{aligned}$$

The conjugations proved in Eqs. (6.5) to (6.8) and the computations performed in [20, Section 6.1] conclude thus the proof of Lemma 6.2. We remark that the zero-th order matrix ${\boldsymbol{A}}_{[0,1]}$ is the sum of the linearly Hamiltonian matrix ${\boldsymbol{A}}_{[-\frac{1}{2},1]}$ and the contributions coming from ${\boldsymbol{A}}_{[0]}$ in Eqs. (6.4) and (6.5). To prove that ${\boldsymbol{J}}_\mathbb {C}{\boldsymbol{A}}_{[0]}$ is linearly Hamiltonian up to homogeneity *N* we then apply Lemma A.15 to each homogeneous component of the operators in Eqs. (6.4) and (6.5) which are linearly Hamiltonian thanks to Lemma A.5. $\square$

### Diagonalization at the Highest Order

In the present section, we diagonalize the quasi-linear contribution

$$\begin{aligned} {\boldsymbol{J}}_{\mathbb {C}} {{{\,\mathrm{Op^{BW}}\,}}\left( {\boldsymbol{A}}^{(0)}_{\frac{3}{2}}\left( U;x \right) \omega _{\gamma , {\texttt{b}}}\left( \xi \right) \right) },\end{aligned}$$

which reduces to the diagonalization of the matrix

6.9 $$\begin{aligned} {\boldsymbol{J}}_{\mathbb {C}} {\boldsymbol{A}}^{(0)}_{\frac{3}{2}}\left( U;x \right) = \textrm{i}\begin{bmatrix} -\left( 1+\texttt{f}\left( U;x \right) \right) &  - \texttt{f}\left( U;x \right) \\ \texttt{f}\left( U;x \right) &  1+ \texttt{f}\left( U;x \right) \end{bmatrix} , \end{aligned}$$

where $\texttt{f}\left( U;x \right)$ is defined in (4.3). The eigenvalues of (6.9) are given by $\pm \textrm{i}\ \lambda \left( U;x \right)$ where

6.10 $$\begin{aligned} \lambda \left( U;x \right) \triangleq \sqrt{1+2\texttt{f}\left( U;x \right) } \end{aligned}$$

and we have that $\lambda \left( U;x \right) -1\in \Sigma \mathcal {F}^{{\mathbb {R}}}_{K, 0, 1}\left[ \epsilon _0, N \right]$. Defining

$$\begin{aligned} \texttt{h}\triangleq \frac{1+\texttt{f}+\lambda }{\sqrt{\left( 1+\texttt{f}+\lambda \right) ^2 - \texttt{f}^2}} \ ,  &   \texttt{g}\triangleq \frac{-\texttt{f}}{\sqrt{\left( 1+\texttt{f}+\lambda \right) ^2 - \texttt{f}^2}} , \end{aligned}$$

the symmetric and symplectic matrix

6.11 $$\begin{aligned} {\boldsymbol{F}} \triangleq \begin{bmatrix} \texttt{h} &  \texttt{g} \\ \texttt{g} &  \texttt{h} \end{bmatrix} \in \left( \Sigma \mathcal {F}^{{\mathbb {R}}}_{K, 0, 0}\left[ \epsilon _0, N \right] \right) ^{2\times 2}\ ,  &   {\boldsymbol{F}}^{-1} \triangleq \begin{bmatrix} \texttt{h} &  - \texttt{g} \\ - \texttt{g} &  \texttt{h} \end{bmatrix} \in \left( \Sigma \mathcal {F}^{{\mathbb {R}}}_{K, 0, 0}\left[ \epsilon _0, N \right] \right) ^{2\times 2} \ , \end{aligned}$$

which are such that

6.12 $$\begin{aligned} {\boldsymbol{F}} - \text {Id}, \ {\boldsymbol{F}}^{-1}-\text {Id}\in \left( \Sigma \mathcal {F}^{{\mathbb {R}}}_{K, 0, 1}\left[ \epsilon _0, N \right] \right) ^{2\times 2} \ , \end{aligned}$$

diagonalize (6.9) in the sense that

$$\begin{aligned} {\boldsymbol{F}}^{-1} {\boldsymbol{J}}_{\mathbb {C}} {\boldsymbol{A}}^{(0)}_{\frac{3}{2}} {\boldsymbol{F}} = \begin{bmatrix} -\textrm{i}\lambda &  0 \\ 0 &  \textrm{i}\lambda \end{bmatrix} . \end{aligned}$$

We prove the following result.

#### Lemma 6.3

Let $N\in {\mathbb {N}}$ and $\varrho > 0$, for any $K \in {\mathbb {N}}^*$ there are $s_0 > 0$, $\epsilon _0 > 0$ such that, for any solution $U \in B^{K}_{s_0}\left( I;\epsilon _0 \right)$ of (6.2) there exists a real-to-real invertible matrix of spectrally localized maps ${\boldsymbol{\Psi }}_1(U)$ such that

1. ${\boldsymbol{\Psi }}_1(U), \ {\boldsymbol{\Psi }}_1(U)^{-1} \in \left( \mathcal {S}^0_{K,0, 0}\left[ \epsilon _0 \right] \right) ^{2\times 2}$ are linearly symplectic as per Definition A.3. Moreover, ${\boldsymbol{\Psi }}_1(U) - \text {Id}\in \left( \Sigma \mathcal {S}^{0}_{K, 0, 1}\left[ \epsilon _0, N \right] \right) ^{2\times 2}.$
2. The variable $U_1 \triangleq {\boldsymbol{\Psi }}_1(U)U_0$ solves
  6.13 $$\begin{aligned} \begin{aligned} \partial _t U_1&= {{\,\mathrm{Op^{BW}_{vec}}\,}}\left( \textrm{i}\ \lambda \left( U;x \right) \omega _{\gamma , {\texttt{b}}}\left( \xi \right) - \textrm{i}\ V_{\texttt{b}}^{(1)}\left( U;x \right) \xi \right) U_1 \\&\quad + {\boldsymbol{J}}_{\mathbb {C}} {{{\,\mathrm{Op^{BW}}\,}}\left( {\boldsymbol{A}}_{\frac{1}{2}}^{(1)}\left( U;x \right) \left| \xi \right| ^{\frac{1}{2}} + {\boldsymbol{A}}_{\left[ 0;1 \right] }\left( U;x,\xi \right) \right) } U_1 + {\boldsymbol{R}}_{\left[ 1 \right] }\left( U \right) U_1, \end{aligned} \end{aligned}$$
  where
  $\omega _{\gamma , {\texttt{b}}}\left( \xi \right) \in \Gamma ^{\frac{3}{2}}_0$ is defined in (2.19);
  $\lambda \left( U;x \right)$ is defined in (6.10);
  $$V_{\texttt{b}}^{(1)} \left( U;x \right) \in \Sigma \mathcal {F}^{{\mathbb {R}}}_{K, 0,1}\left[ \epsilon _0, N \right] $$;
  $${\boldsymbol{A}}_{\frac{1}{2}}^{(1)} \left( U;x \right) \triangleq \left( \texttt{h}\left( U;x \right) + \texttt{g}\left( U;x \right) \right) ^2 {\boldsymbol{A}}_{\frac{1}{2}}^{(0)}\left( U;x \right) \in \left( \Sigma \mathcal {F}^{{\mathbb {R}}}_{K, 0,1}\left[ \epsilon _0, N \right] \right) ^{2\times 2}$$.

#### Proof

Arguing as in [20, Lemma 6.4] the 1-flow ${\boldsymbol{\Psi }}_1\left( U \right) \triangleq \left. {\boldsymbol{\Psi }}^\tau \left( U \right) \right| _{\tau =0}$ of

6.14 $$\begin{aligned} \left\{ \begin{aligned}&\partial _\tau {\boldsymbol{\Psi }}^\tau \left( U \right) = {\boldsymbol{J}}_{\mathbb {C}} {{{\,\mathrm{Op^{BW}}\,}}\left( \begin{bmatrix} \textrm{i}\ \log \left( \texttt{h}\left( U;x \right) + \texttt{g}\left( U;x \right) \right) &  0 \\ 0 &  -\textrm{i}\ \log \left( \texttt{h}\left( U;x \right) + \texttt{g}\left( U;x \right) \right) \end{bmatrix} \right) }{\boldsymbol{\Psi }}^\tau , \\&{\boldsymbol{\Psi }}^0\left( U \right) = \text {Id}\end{aligned} \right. \end{aligned}$$

is such that

6.15 $$\begin{aligned} {\boldsymbol{\Psi }}_1\left( U \right) = {{{\,\mathrm{Op^{BW}}\,}}\left( {\boldsymbol{F}}^{-1}\left( U;x \right) \right) } + {\boldsymbol{R}}\left( U \right) ,  &   {\boldsymbol{\Psi }}_1\left( U \right) ^{-1} = {{{\,\mathrm{Op^{BW}}\,}}\left( {\boldsymbol{F}} \left( U;x \right) \right) } + {\boldsymbol{R}}\left( U \right) . \end{aligned}$$

Since the generator in (6.14) is linearly Hamiltonian, Lemma A.6 ensures that ${\boldsymbol{\Psi }}_1\left( U \right)$ is a linearly symplectic, spectrally localized map in $\left( \Sigma \mathcal {S}^0_{K,K',1}[r,N] \right) ^{2\times 2}$. Thus, the new variable $U_1\triangleq {\boldsymbol{\Psi }}_1\left( U \right) U_0$ solves (cf. (6.2))

6.16 $$\begin{aligned} \partial _t U_1 = {\boldsymbol{\Psi }}_1\left( U \right) {\boldsymbol{J}}_{\mathbb {C}} \ {{{\,\mathrm{Op^{BW}}\,}}\left( {\boldsymbol{A}}_{\frac{3}{2}}^{(0)}\left( U;x \right) \ \omega _{\gamma , {\texttt{b}}}\left( \xi \right) + {\boldsymbol{A}}_{\frac{1}{2}}^{(0)} \left( U;x \right) \ \left| \xi \right| ^{\frac{1}{2}} + {\boldsymbol{A}}_{\left[ 0;1 \right] }\left( U;x, \xi \right) \right) } {\boldsymbol{\Psi }}_1\left( U \right) ^{-1} U_1 \nonumber \\ +\partial _t {\boldsymbol{\Psi }}_1\left( U \right) {\boldsymbol{\Psi }}_1\left( U \right) ^{-1} U_1 - {\boldsymbol{\Psi }}_1\left( U \right) {{{\,\mathrm{Op^{BW}}\,}}\left( \textrm{i}V_{\texttt{b}}\left( \textsf{M}U;x \right) \xi \right) } {\boldsymbol{\Psi }}_1\left( U \right) ^{-1} U_1 + {\boldsymbol{\Psi }}_1\left( U \right) {\boldsymbol{R}}\left( U \right) {\boldsymbol{\Psi }}_1\left( U \right) ^{-1} U_1.\nonumber \\ \end{aligned}$$

Following the same computations as in [20, Eq. (6.3)] we have that

6.17 $$\begin{aligned} \begin{aligned}&{\boldsymbol{\Psi }}_1\left( U \right) {\boldsymbol{J}}_{\mathbb {C}} \ {{{\,\mathrm{Op^{BW}}\,}}\left( {\boldsymbol{A}}_{\frac{3}{2}}^{(0)}\left( U;x \right) \ \omega _{\gamma , {\texttt{b}}}\left( \xi \right) \right) } {\boldsymbol{\Psi }}_1\left( U \right) ^{-1} \\&= {{{\,\mathrm{Op^{BW}}\,}}\left( \textrm{i}\lambda \left( U;x \right) \omega _{\gamma , {\texttt{b}}}\left( \xi \right) \begin{bmatrix} -1 &  0 \\ 0 &  1 \end{bmatrix} + A_{\left[ -\frac{1}{2};0 \right] }\left( U;x,\xi \right) \right) } + {\boldsymbol{R}}\left( U \right) . \end{aligned} \end{aligned}$$

Similar computations give us the conjugation

6.18 $$\begin{aligned} \begin{aligned}&{\boldsymbol{\Psi }}_1\left( U \right) {\boldsymbol{J}}_{\mathbb {C}} \ {{{\,\mathrm{Op^{BW}}\,}}\left( {\boldsymbol{A}}_{\frac{1}{2}}^{(0)}\left( U;x \right) \ \left| \xi \right| ^{\frac{1}{2}} \right) } {\boldsymbol{\Psi }}_1\left( U \right) ^{-1} \\&= {\boldsymbol{J}}_{\mathbb {C}}{{{\,\mathrm{Op^{BW}}\,}}\left( \left( \texttt{h}\left( U;x \right) + \texttt{g}\left( U;x \right) \right) ^2 {\boldsymbol{A}}^{(0)}_{\frac{1}{2}} \left( U;x \right) \ \left| \xi \right| ^{\frac{1}{2}} + A_{\left[ -\frac{1}{2}; 0 \right] }\left( U;x, \xi \right) \right) } + {\boldsymbol{R}}\left( U \right) \end{aligned} \end{aligned}$$

and

6.19 $$\begin{aligned} \begin{aligned} {\boldsymbol{\Psi }}_1\left( U \right) {{{\,\mathrm{Op^{BW}}\,}}\left( {\boldsymbol{A}}_{\left[ 0;1 \right] }\left( U;x,\xi \right) \right) } {\boldsymbol{\Psi }}_1\left( U \right) ^{-1} =&\ {{{\,\mathrm{Op^{BW}}\,}}\left( {\boldsymbol{A}}_{\left[ 0;1 \right] }\left( U;x,\xi \right) \right) } + {\boldsymbol{R}}\left( U \right) , \end{aligned} \end{aligned}$$

while from Eqs. (6.15) and (6.12) we obtain that

6.20 $$\begin{aligned} \partial _t {\boldsymbol{\Psi }}_1\left( U \right) {\boldsymbol{\Psi }}_1\left( U \right) ^{-1} = {{{\,\mathrm{Op^{BW}}\,}}\left( {\boldsymbol{A}}_{\left[ 0;1 \right] }\left( U;x, \xi \right) \right) } + {\boldsymbol{R}}_{\left[ 1 \right] }\left( U \right) , \end{aligned}$$

and standard symbolic computations (cf. Proposition 3.15 and Eq. (6.15)) give that

6.21 $$\begin{aligned}&{\boldsymbol{\Psi }}_1\left( U \right) {{\,\mathrm{Op^{BW}_{vec}}\,}}\left( \textrm{i}V_{\texttt{b}}\left( U;x \right) \xi \right) {\boldsymbol{\Psi }}_1\left( U \right) ^{-1} = {{\,\mathrm{Op^{BW}_{vec}}\,}}\left( \textrm{i}V_{\texttt{b}}^{(1)} \left( U;x \right) \xi \right) + {\boldsymbol{R}}\left( U \right) , \nonumber \\&V_{\texttt{b}}^{(1)} \left( U;x \right) \in \Sigma \mathcal {F}^{{\mathbb {R}}}_{K, 0, 1}\left[ \epsilon _0, N \right] . \end{aligned}$$

We conclude plugging Eqs. (6.17) to (6.21) in Eq. (6.16). We remark that the zero-th order matrix ${\boldsymbol{A}}_{[0,1]}$ is the sum of ${\boldsymbol{A}}_{[-\frac{1}{2};0]}$ form Eqs. (6.17) and (6.18) and ${\boldsymbol{A}}_{[0;1]}$ from Eqs. (6.19) and (6.20). To prove that ${\boldsymbol{J}}_\mathbb {C}{\boldsymbol{A}}_{[0;1]}$ is linearly symplectic we then apply Lemma A.15 to each homogeneous component of the spectrally localized operators in Eqs. (6.17) to (6.20), which are linearly Hamiltonian thanks to Lemma A.5. $\square$

### Reduction to Constant Coefficients at the Highest Order

#### Lemma 6.4

(Reduction of the highest order) Let $N \in {\mathbb {N}}$ and $\varrho > 2(N + 1)$. Then for any $K \in {\mathbb {N}}^*$ there are $s_0 > 0$, $\epsilon _0 > 0$ such that for any solution $U \in B^{K}_{s_0}\left( I;\epsilon _0 \right)$ of (6.13), there exists a real-to-real invertible matrix of spectrally localized maps ${\boldsymbol{\Psi }}_2(U)$ satisfying

1. ${\boldsymbol{\Psi }}_2(U),\, {\boldsymbol{\Psi }}_2(U) \in \left( \mathcal {S}^0_{K,0,0}[\epsilon _0] \right) ^{2\times 2}$ are linearly symplectic as per Definition A.3. Moreover, ${\boldsymbol{\Psi }}_2(U) - \text {Id}\in \left( \Sigma \mathcal {S}^{N+1}_{K,0,2}[\epsilon _0,N] \right) ^{2\times 2}$;
2. The variable $U_2 \triangleq {\boldsymbol{\Psi }}_2(U)U_1$ solves the system 6.22 where
  is a *x*-independent function in $\Sigma \mathcal {F}^{{\mathbb {R}}}_{K,0,2}[\epsilon _0,N]$ and $\omega _{\gamma ,{\texttt{b}}}(\xi )$ is defined in (2.19);
  $V_{\texttt{b}}^{(2)} (U;x)$ is a real valued function in $\left( \Sigma \mathcal {F}^{{\mathbb {R}}}_{K,1,1}[\epsilon _0,N] \right) ^2$;
  $$ {\boldsymbol{A}}_{\frac{1}{2}}^{(2)}\left( U;x \right) \triangleq A_{\frac{1}{2}}^{(2)}\left( U;x \right) \begin{bmatrix} 1& 1\\ 1& 1 \end{bmatrix} $$ where $$ A_{\frac{1}{2}}^{(2)}\left( U;x \right) \in \left( \Sigma \mathcal {F}^{{\mathbb {R}}}_{K, 0, 1}\left[ \epsilon _0, N \right] \right) ^{2\times 2} $$;
  $$\textbf{R}\left( U \right) \in \Sigma \mathcal {R}^{-\varrho +2\left( N+1 \right) }_{K, 1, 1}\left[ \epsilon _0,N \right] $$.

#### Proof

We refer the interested reader to [20, Lemma 6.7], the only difference being the conjugation of the term of order 1/2, which is achieved by standard paracomposition theorems, such as [61, Theorem 3.27]. Notice that conjugation worsens the regularizing properties of the smoothing operator in Eq. (6.22). $\square$

### Diagonalization Up to Smoothing Remainders

In this section, we block-diagonalize the Kelvin-Helmholtz system up to a smoothing remainder.

#### Lemma 6.5

(Diagonalization to arbitrary order) Let $N \in {\mathbb {N}}$ and $\varrho \gg N$. Then for any $n \in {\mathbb {N}}\cup \left\{ -1 \right\}$, there exists $K' \triangleq K'(n) {\geqslant }0$ such that for all $K \geqslant K' + 1$, there exist $s_0 > 0$ and $\epsilon _0 > 0$ such that for any solution $U \in B^{K}_{s_0, \mathbb {R}}\left( I;\epsilon _0 \right)$ of Eq. (6.22), there exists a real-to-real invertible matrix of spectrally localized maps ${\boldsymbol{\Phi }}_n(U)$ satisfying

1. ${\boldsymbol{\Phi }}_n(U),\, {\boldsymbol{\Phi }}_n(U)^{-1} \in \left( \mathcal {S}^0_{K,0,0}[\epsilon _0] \right) ^{2\times 2}$ are linearly symplectic up to homogeneity *N* as per Definition A.4. Moreover, ${\boldsymbol{\Phi }}_n(U) - \text {Id}\in \left( \Sigma \mathcal {S}^0_{K,K',1}[r,N] \right) ^{2\times 2}$;
2. The variable $U_{n+3} \triangleq {\boldsymbol{\Phi }}_n(U) U_{2}$ solves 6.23 where
  $$a^{\left( n \right) }_{\frac{1}{2}}\left( U;x \right) \in \mathcal {F}^{{\mathbb {R}}}_{K,0,1}\left[ \epsilon _0,N \right] $$,
  $a^{(n)}_0(U;x,\xi )$ is a symbol in $\Sigma \Gamma ^0_{K,K',1}[\epsilon _0,N]$ with $\text {Im}a^{(n)}_0(U;x,\xi ) \in \Gamma ^0_{K,K',N+1}[\epsilon _0]$,
  $$\textbf{R}\left( U \right) \in \Sigma \mathcal {R}^{-\varrho +2\left( N+1 \right) }_{K, 1, 1}\left[ \epsilon _0,N \right] $$.

*Proof*

#### Step 1

(Reduction of the term of order 1/2) Notice that the term of order 1/2 can be written as

$$\begin{aligned}&{\boldsymbol{J}}_{\mathbb {C}}{\boldsymbol{A}}_{\frac{1}{2}}^{(2)}\left( U;x \right) \left| \xi \right| ^{\left. 1 / 2 \right. } = - \textrm{i}a_{\left. 1 / 2 \right. }^{(-1)}\left( U;x \right) \begin{bmatrix} 1 &  1 \\ -1 &  -1 \end{bmatrix} \left| \xi \right| ^{\frac{1}{2}} , \\&a_{\left. 1 / 2 \right. }^{(-1)}\left( U;x \right) \in \Sigma \mathcal {F}^{{\mathbb {R}}}_{K, 0, 1}\left[ \epsilon _0, N \right] . \end{aligned}$$

Let nowand let $\left( {\boldsymbol{\Phi }}_{{\boldsymbol{F}}^{\left( -1 \right) }}^\tau \left( U \right) \right) _{\left| \tau \right| \leqslant 1}$ be the flow generated by ${{{\,\mathrm{Op^{BW}}\,}}\left( {\boldsymbol{F}}^{\left( -1 \right) }\left( U;x, \xi \right) \right) }$ with initial condition ${\boldsymbol{\Phi }}_{{\boldsymbol{F}}^{\left( -1 \right) }}^0 \left( U \right) = \text {Id}$ as per Lemma A.6. We denote with ${\boldsymbol{\Phi }}\left( U \right) \triangleq {\boldsymbol{\Phi }}_{{\boldsymbol{F}}^{\left( -1 \right) }}^1 \left( U \right)$. Since $\boldsymbol{F}^{(-1)}$ is linearly Hamiltonian, Lemma A.6 ensures that ${\boldsymbol{\Phi }}\left( U \right)$ is a linearly symplectic, spectrally localized map in $\left( \Sigma \mathcal {S}^0_{K,K',1}[r,N] \right) ^{2\times 2}$. Let us define

$$\begin{aligned} U_3\triangleq {\boldsymbol{\Phi }}_{{\boldsymbol{F}}^{\left( -1 \right) }}^1 \left( U \right) U_2 = {\boldsymbol{\Phi }} \left( U \right) U_2 , \end{aligned}$$

which is the solution of6.24The following conjugation rule apply (cf. [68, Lemma A.1]) setting $\textbf{F}\triangleq {{{\,\mathrm{Op^{BW}}\,}}\left( {\boldsymbol{F}}^{\left( -1 \right) }\left( U;x, \xi \right) \right) }$

6.25 $$\begin{aligned} {\boldsymbol{\Phi }}\left( U \right) {\boldsymbol{M}}\left( U \right) {\boldsymbol{\Phi }}\left( U \right) ^{-1} =&\ {\boldsymbol{M}} + \sum _{q=1}^L\frac{1}{q!} \text {Ad}^q_\textbf{F}\left[ \boldsymbol{M} \right] + \frac{1}{L!}\int _0^1 \left( 1-\tau \right) ^L {\boldsymbol{\Phi }}_{{\boldsymbol{F}}^{\left( -1 \right) }}^\tau \left( U \right) \text {Ad}^{L+1}_\textbf{F}\left[ \boldsymbol{M} \right] {\boldsymbol{\Phi }}_{{\boldsymbol{F}}^{\left( -1 \right) }}^\tau \left( U \right) ^{-1} \text {d}\tau , \end{aligned}$$

6.26 $$\begin{aligned} \left( \partial _t {\boldsymbol{\Phi }}\left( U \right) \right) {\boldsymbol{\Phi }}\left( U \right) ^{-1} =&\ {\boldsymbol{J}}_{\mathbb {C}}{{{\,\mathrm{Op^{BW}}\,}}\left( {\boldsymbol{A}}_{\left[ -1;1 \right] }\left( U;x, \xi \right) \right) } + {\boldsymbol{R}}_{\left[ 1 \right] }\left( U \right) . \end{aligned}$$

An application of Eq. (6.25) for $L> \frac{3+2\varrho }{4} -1$ combined with symbolic calculus considerations give us that6.27in which the off-diagonal terms of order 1/2 have been canceled. Applying Eqs. (6.27) to (6.26) to (6.24) (and renaming ${\boldsymbol{\Phi }} \left( U \right) \textbf{R} (U){\boldsymbol{\Phi }} \left( U \right) ^{-1}\leadsto \textbf{R} (U)$ in light of Proposition 3.17, Item 2 we obtain that6.28Notice that the conjugation rule in Eq. (6.25) does not modify the principal symbol of the transport term. Moreover the zero-th order matrix ${\boldsymbol{A}}_{[0,1]}$ is the sum of ${\boldsymbol{A}}_{[-1;1]}$ form Eq. (6.26) and ${\boldsymbol{A}}_{[0;1]}$ from Eq. (6.27). To prove that ${\boldsymbol{J}}_\mathbb {C}{\boldsymbol{A}}_{[0;1]}$ is linearly symplectic we then apply Lemma A.15 to each homogeneous components of the spectrally localized operators in Eqs. (6.26) and (6.27), which are linearly Hamiltonian thanks to Lemma A.5.

#### Step 2

(Reduction at non-positive order) We sketch the proof which is very similar to the one outlined in Step 1, we refer the interested reader to [20, Lemma 6.8]. The proof is performed by induction on $n\in {\mathbb {N}}$, the case $n=0$ is proven in Step 1, so that we can consider the case $n\leadsto n+1$. Suppose Eq. (6.23) holds, we have to find a bounded, lineary-symplectic transformation that pushes the off diagonal terms of ${\boldsymbol{J}}_{\mathbb {C}}{{{\,\mathrm{Op^{BW}}\,}}\left( {\boldsymbol{A}}_{\left[ -n;K'+1 \right] }\right) }$ to lower order, similarly as in was done in Step 1. Since the matrix ${\boldsymbol{A}}_{\left[ -n; K'+1 \right] }$ is of the form

6.29 $$\begin{aligned} {\boldsymbol{J}}_{\mathbb {C}}{\boldsymbol{A}}_{\left[ -n; K'+1 \right] } = {\boldsymbol{J}}_{\mathbb {C}}\begin{bmatrix} -\textrm{i}\bar{b}^\vee _{\left[ -n \right] } &  - \bar{a}^\vee _{\left[ -n \right] } \\ - {a} _{\left[ -n \right] } &  \textrm{i}\bar{b} _{\left[ -n \right] } \end{bmatrix},  &   {a} _{\left[ -n \right] }, \ \bar{b} _{\left[ -n \right] }\in \Sigma \Gamma ^{-n}_{K, K'+1, 1}\left[ \epsilon _0, N \right] , \end{aligned}$$

such cancellation is achieved via conjugation by the flow

$$\begin{aligned} \left\{ \begin{aligned}&\partial _\tau {\boldsymbol{\Phi }}_{{\boldsymbol{F}}^{(n)}}^\tau (U) = {{{\,\mathrm{Op^{BW}}\,}}\left( {\boldsymbol{F}}^{(n)}(U)\right) } {\boldsymbol{\Phi }}_{{\boldsymbol{F}}^{(n)}}^\tau (U), \\&{\boldsymbol{\Phi }}_{{\boldsymbol{F}}^{(n)}}^0(U) = \text {Id}, \end{aligned} \right.  &   {\boldsymbol{F}}^{(n)}(U) \triangleq \left[ \begin{array}{cc} 0 &  f_{-n-\frac{3}{2}} \\ \overline{f_{-n-\frac{3}{2}}^\vee } &  0 \end{array} \right] , \end{aligned}$$

and defining the variable $U_{n+4}\triangleq {\boldsymbol{\Phi }}_{{\boldsymbol{F}}^{\left( n \right) }}^1\left( U \right) U_{n+3}$ and we refer the reader to [20, Lemma 6.8] for further details. As the matrix in (6.29) is linearly Hamiltonian up to homogeneity *N* by inductive hypothesis, the explicitly defined generator $\boldsymbol{F}^{(n)}$ is linearly Hamiltonian up to homogeneity *N* as well. Thus Lemma A.6 ensures that ${\boldsymbol{\Phi }}_{{\boldsymbol{F}}^{(n)}}^\tau (U)$ is a linearly symplectic, spectrally localized map in $\left( \Sigma \mathcal {S}^0_{K,K',1}[r,N] \right) ^{2\times 2}$. The bounded, linearly symplectic transformation is thus defined as

$$\begin{aligned} {\boldsymbol{\Phi }}_n \left( U \right) \triangleq \prod _{\textsf{j}=-1}^n {\boldsymbol{\Phi }}_{{\boldsymbol{F}}^{\left( \textsf{j} \right) }}^1\left( U \right) . \end{aligned}$$

$$\square $$

We can thus apply Lemma 6.5 setting $n\triangleq n_1\left( \varrho \right) \geqslant -\varrho +2\left( N+1 \right)$ so that ${\boldsymbol{J}}_{\mathbb {C}}{{{\,\mathrm{Op^{BW}}\,}}\left( {\boldsymbol{A}}_{\left[ -n;K'+1 \right] }\right) }$ can be incorporated in the smoothing reminder $\textbf{R}\left( U \right)$, thus setting $Z\triangleq U_{n_1+4}$ we obtain that *Z* solves the evolution equation6.30

### Reduction to Constant Coefficients up to Smoothing Remainders

#### Notation 6.6

From now on we shall denote functions and symbols that are *x*-independent with calligraphic lower- and upper-case letters.

#### Lemma 6.7

Let $N \in {\mathbb {N}}$ and $\varrho >3(N+1)$. Then for any $n\in {\mathbb {N}}$ there is $K''\triangleq K''\left( \varrho ,n \right) >0$ such that for all $K\geqslant K''+1$ there are $s_0 >0$, $\epsilon _0>0$ such that for any solution $U \in B_{s_0,{\mathbb {R}}}^K(I;\epsilon _0)$ of (5.9), there exists a real-to-real invertible matrix of spectrally localized maps ${\boldsymbol{\Theta }}_n(U)$ such that

1. ${\boldsymbol{\Theta }}_n(U),{\boldsymbol{\Theta }}_n(U)^{-1}\in \left( \mathcal {S}_{K, K'',0}^0 [\epsilon _0] \right) ^{2\times 2}$ are linearly symplectic up to homogeneity *N* according to Definition A.4. Moreover, ${\boldsymbol{\Theta }}_n(U)-\text {Id}\in \left( \Sigma \mathcal {S}^{\frac{N+1}{2}}_{K,K'',1}[\epsilon _0,N] \right) ^{2\times 2}.$
2. If *Z* solves (6.30) then the variable $Z_{n}\triangleq {\boldsymbol{\Theta }}_n(U) Z$ solves 6.31 with the *x*–independent symbol 6.32 where
  ;
  the function  is *x*-independent;
  the function  is *x*-independent;
  the symbol  is *x*–independent and its imaginary part  is in $\Gamma ^{0}_{K,K'',N+1}[\epsilon _0]$;
  the symbol $a_{-\frac{n}{2}}(U;t, x,\xi )$ belongs to $\Sigma \Gamma ^{-\frac{n}{2}}_{K,K''+1,1}[\epsilon _0, N]$ and its imaginary part $\text {Im}a_{-\frac{n}{2}}(U;t, x,\xi )$ is in $\Gamma ^{-\frac{n}{2}}_{K,K''+1,N+1}[\epsilon _0]$;
  $\pmb { \textsf{R} }(U;t)$ is a real-to-real matrix of smoothing operators in $\left( \Sigma \mathcal {R}^{-\varrho +3(N+1)}_{K,K''+1, 1}[\epsilon _0, N] \right) ^{2\times 2}$.

#### Proof

The proof consist of a minor modification of the proof of [20, lemma 6.9], so we refer the interested reader to [20, lemma 6.9] for details. $\square$

Finally we prove Proposition 6.1.

#### Proof of Proposition 6.1

In Lemma 6.7 we set

$$\begin{aligned}n_2\triangleq n_2\left( \varrho ,N \right) \triangleq n \geqslant 2\left( \varrho + 3\left( N+1 \right) \right) ,\qquad \underline{K'}\triangleq K'' + 1\end{aligned}$$

and we define

$$\begin{aligned}W\triangleq Z_{n_2} = {\boldsymbol{B}}\left( U;t \right) U,\end{aligned}$$

where

$$\begin{aligned} {\boldsymbol{B}}\left( U;t \right) \triangleq {\boldsymbol{\Theta }}_{n_2}\left( U;t \right) \circ {\boldsymbol{\Phi }}_{n_1}\left( U;t \right) \circ {\boldsymbol{\Psi }}_2\left( U;t \right) \circ {\boldsymbol{\Psi }}_1\left( U;t \right) \circ {\boldsymbol{G}}\left( U;t \right) . \end{aligned}$$

At last we set  and we conclude. $\square$

## Hamiltonian Birkhoff Normal Form and Energy Estimate

In this section we finally prove the almost global existence Theorem 1.1. The previous reduction broke the Hamiltonian structure of the system. Therefore, a first step is to recover the complex Hamiltonian formulation through a symplectic correction. The corresponding result is stated in Proposition 7.1 and serves as the foundation for the subsequent normal form analysis. Then, in the technical subsection 7.1, we introduce the class of super-action preserving Hamiltonians that play a central role in the forthcoming analysis, as they do not contribute to the energy estimate. Next, in Section 7.2, we perform a Birkhoff normal form procedure, which extracts the Hamiltonian super-action preserving property up to homogeneity $N+1$, for a given fixed integer *N*. Finally, we implement in Section 7.3 the energy estimate allowing to conclude the desired Theorem.

The transformation ${\boldsymbol{B}}(U)$ in Proposition 6.1 destroyed the Hamiltonian property of the system. However ${\boldsymbol{B}}(U)$ was still linearly symplectic and therefore, we can recover the complex Hamiltonian structure by applying the abstract Darboux Theorem of Berti-Maspero-Murgante [20, Theorem 7.1], see also Theorem A.16. The corresponding result reads as follows:

### Proposition 7.1

(Hamiltonian reduction up to smoothing operators) Let $N \in \mathbb {N}$ and $\varrho >{\varrho (N)} \triangleq 3(N+1)+ \frac{3}{2} (N+1)^3$. Then, for any $K\geqslant \underline{K}'$ (fixed in Proposition 6.1) there are $s_0>0, \epsilon _0 > 0$, such that for any solution $U \in B_{s_0,\mathbb {R}}^K(I;\epsilon _0)$ of (5.9), there exists a real-to-real matrix of pluri–homogeneous smoothing operators $\pmb {R}(U)$ in $\left( \Sigma _1^N \tilde{\mathcal {R}}^{-\varrho '}_q \right) ^{2\times 2}$ for any $\varrho '\geqslant 0$, such that, defining

7.1 $$\begin{aligned} Z_0\triangleq \big ( \textrm{Id}+ \boldsymbol{R}( \Phi (U)) \big ) \Phi (U),\qquad \Phi (U)\triangleq \boldsymbol{B}(U;t)U, \end{aligned}$$

where $\boldsymbol{B}(U;t)$ is defined in Proposition 6.1, the following holds true:

- **i** **Symplecticity:** The non-linear map $\big ( \textrm{Id}+ \boldsymbol{R}( \cdot ) \big )\circ \Phi$ in (7.1) is symplectic up to homogeneity *N* according to Definition A.11.
- **ii** **Conjugation:** The variable $Z_0$ solves the *Hamiltonian system up to homogeneity N* (cf. Definition A.10). 7.2 where
  - $\boldsymbol{\omega }_{\gamma ,{\texttt{b}}}(D) \triangleq {{\,\mathrm{Op^{BW}_{vec}}\,}}\left( \omega _{\gamma ,{\texttt{b}}}(\xi )\right)$ is a matrix in the space $\left( \Gamma ^{3/2}_0 \right) ^{2\times 2}$;
  is a pluri-homogeneous, real valued symbol, independent of *x*, in $\Sigma _{2}^N \widetilde{\Gamma }^{\frac{3}{2}}_q$;
  is a non-homogeneous symbol, independent of *x*, in $\Gamma ^{\frac{3}{2}}_{K,\underline{K}',N+1}[\epsilon _0]$ with imaginary part  in $\Gamma ^{0}_{K, \underline{K}',N+1}[\epsilon _0]$;
  - $\boldsymbol{R}_{\leqslant N}(Z_0)$ is a real-to-real matrix of smoothing operators in $\left( \Sigma _{1}^N \widetilde{\mathcal {R}}^{-\varrho +\varrho (N)}_q \right) ^{2\times 2}$;
  - $\boldsymbol{R}_{>N}(U;t)$ is a real-to-real matrix of non-homogeneous smoothing operators in $\left( \mathcal {R}^{-\varrho +\varrho (N)}_{K,\underline{K}',N+1}[\epsilon _0] \right) ^{2\times 2}$.
- **iii** **Boundedness:** The variable $Z_0 = \textbf{M}_0(U;t)U$ with $\textbf{M}_{0}(U;t) \in \left( \mathcal {M}^0_{K,\underline{K}'-1,0}[\epsilon _0] \right) ^{2 \times 2}$ and for any $s \geqslant s_0$, there is $0< \epsilon _0(s)<\epsilon _0$, such that for any $U \in B_{s_0}^K(I;\epsilon _0)\cap C_{*\mathbb {R}}^{K}(I;{\dot{H}}^{s}(\mathbb {T}, \mathbb {C}^2))$, there is a constant $C\triangleq C_{s,K}>0$ such that, for all $k=0,\dots , K-\underline{K}'$,
  7.3 $$\begin{aligned} C^{-1}\Vert U\Vert _{k,s}\leqslant \Vert Z_0 \Vert _{k,s}\leqslant C \Vert U\Vert _{k,s} \, . \end{aligned}$$

### Super-Action Preserving Symbols and Hamiltonian

In this section we define the special class of “super–action preserving" $\texttt{SAP}$ homogeneous symbols and Hamiltonian which will appear in the Birkhoff normal form reduction of the Section 7.2.

#### Definition 7.2

*(*$\texttt{SAP}$ *monomial)* Let $p\in \mathbb {N}^*$. Given $(\mathbf {\jmath }, \mathbf {\sigma })= (j_a,\sigma _a)_{a=1,\dots , p} \in (\mathbb {Z}^*)^p \times \{ \pm \}^p$ we define the multi-index $(\alpha ,\beta ) \in \mathbb {N}^{\mathbb {Z}^*} \times \mathbb {N}^{\mathbb {Z}^*}$ with components, for any $k \in \mathbb {Z}^*$,

7.4 $$\begin{aligned} \begin{aligned}&\alpha _k(\mathbf {\jmath }, \mathbf {\sigma })\triangleq \# \big \{ a = 1, \ldots ,p \, : \, (j_a , \sigma _a) = (k,+) \big \} \, , \\&\beta _k(\mathbf {\jmath }, \mathbf {\sigma })\triangleq \# \big \{ a = 1, \ldots ,p \, : \, (j_a , \sigma _a) = (k,-) \big \} \, . \end{aligned} \end{aligned}$$

We say that a monomial of the form $z_{\mathbf {\jmath }}^{\mathbf {\sigma }} = z_{j_1}^{\sigma _1}\dots z_{j_p}^{\sigma _p}$ is super-action preserving if the associated multi-index $(\alpha ,\beta )= (\alpha (\mathbf {\jmath }, \mathbf {\sigma }),\beta (\mathbf {\jmath }, \mathbf {\sigma }))$ is super-action preserving according to Definition 2.6.

We now introduce the subset $\mathfrak {S}_p$ of the indexes of $\mathfrak {T}_p$ composed by super-action preserving indexes

7.5 $$\begin{aligned} \mathfrak S_p \triangleq \Big \{(\mathbf {\jmath }, \mathbf {\sigma }) \in {\mathfrak {T}}_p \quad \text {s.t.}\quad (\alpha (\mathbf {\jmath }, \mathbf {\sigma }), \beta (\mathbf {\jmath }, \mathbf {\sigma })) \in \mathbb {N}^{\mathbb {Z}^*} \times \mathbb {N}^{\mathbb {Z}^*} \ \text {in} \ (7.4) \ \text {are super action preserving} \Big \} \, . \end{aligned}$$

We remark that the multi-index $(\alpha , \beta )$ associated to $(\mathbf {\jmath }, \mathbf {\sigma }) \in (\mathbb {Z}^*\times \{\pm \})^p$ as in (7.4) satisfies $|\alpha +\beta |=p$ and

7.6 $$\begin{aligned} z_{\mathbf {\jmath }}^{\mathbf {\sigma }}= z^\alpha \bar{z}^\beta \triangleq \prod _{j \in \mathbb {Z}\setminus \{0\} } z_j^{\alpha _j}{\overline{z_j}}^{\beta _j} = \prod _{n \in \mathbb {N}} z_n^{\alpha _n}z_{-n}^{\alpha _{-n}} {\overline{z_n}}^{\beta _n}\overline{z_{-n}}^{\beta _{-n}}\, \, . \end{aligned}$$

It turns out

7.7 $$\begin{aligned} \mathbf {\sigma }\cdot \vec {\omega }_{\gamma ,{\texttt{b}}}(\vec {\jmath })  &   = \sigma _1 {\omega }_{\gamma ,{\texttt{b}}}({j_1})+\dots + \sigma _p{\omega }_{\gamma ,{\texttt{b}}}({j_p})= (\alpha - \beta ) \cdot \mathbf {\omega }_{\gamma ,{\texttt{b}}} \nonumber \\  &   = \sum _{k \in \mathbb {Z}^*} (\alpha _k- \beta _k) {\omega }_{\gamma ,{\texttt{b}}}(k) \, , \end{aligned}$$

where we denote

7.8 $$\begin{aligned} \mathbf {\omega }_{\gamma ,{\texttt{b}}}({\mathbf {\jmath }})\triangleq ( {\omega }_{\gamma ,{\texttt{b}}}({j_1}), \dots , {\omega }_{\gamma ,{\texttt{b}}}({j_p})),\qquad \vec {{\omega }}_{\gamma ,{\texttt{b}}}\triangleq \{ {\omega }_{\gamma ,{\texttt{b}}}(j)\}_{j\in \mathbb {Z}^*}. \end{aligned}$$

#### Remark 7.3

If the monomial $z_{\mathbf {\jmath }}^{\mathbf {\sigma }}$ is super–action preserving then, for any $j \in \mathbb {Z}^*$, the monomial $z_{\mathbf {\jmath }}^{\mathbf {\sigma }}z_j \bar{z}_j$ is super-action preserving as well.

For any $n \in \mathbb {N}^*$, we define the *super-action*

7.9 $$\begin{aligned} J_n \triangleq |z_n|^2 + |z_{-n}|^2. \end{aligned}$$

The following is Lemma 7.7 in [20].

#### Lemma 7.4

The Poisson bracket between a monomial $z_{\mathbf {\jmath }}^{\mathbf {\sigma }}$ and a super-action $J_n$, $n \in \mathbb {N}$, defined in (7.9), is

7.10 $$\begin{aligned} \left\{ z_{\mathbf {\jmath }}^{\mathbf {\sigma }}, J_n \right\} = \textrm{i}\big (\beta _n + \beta _{-n} - \alpha _n - \alpha _{-n} \big ) \, z_{\mathbf {\jmath }}^{\mathbf {\sigma }} \, , \end{aligned}$$

where $(\alpha , \beta )= (\alpha (\mathbf {\jmath }, \mathbf {\sigma }),\beta (\mathbf {\jmath }, \mathbf {\sigma }))$ is the multi-index defined in (7.4). In particular a super-action preserving monomial $z_{\mathbf {\jmath }}^{\mathbf {\sigma }}$ (according to Definition 7.2) Poisson commutes with any super-action $J_n$, $n \in \mathbb {N}$.

We now define a super-action preserving Hamiltonian.

#### Definition 7.5

*(*$\texttt{SAP}$ *Hamiltonian)* Let $p \in \mathbb {N}$. A $(p+2)$–homogeneous super-action preserving Hamiltonian $H^{(\texttt{SAP})}_{p+2}(Z)$ is a real function of the form

$$\begin{aligned} H^{(\texttt{SAP})}_{p+2}(Z)=\frac{1}{p+2} \sum _{\begin{array}{c} (\mathbf {\jmath }_{p+2},\mathbf {\sigma }_{p+2})\in {\mathfrak {S}}_{p+2} \end{array} } H_{\mathbf {\jmath }_{p+2}}^{\mathbf {\sigma }_{p+2}} z_{\mathbf {\jmath }_{p+2}}^{\mathbf {\sigma }_{p+2}} \end{aligned}$$

where $\mathfrak {S}_{p+2}$ is defined as in (7.5). A pluri-homogeneous super-action preserving Hamiltonian is a finite sum of homogeneous super-action preserving Hamiltonians. A Hamiltonian vector field is super-action preserving if it is generated by a super-action preserving Hamiltonian.

We now define a super-action preserving symbol.

#### Definition 7.6

*(*$\texttt{SAP}$ *symbol)* Let $p \in \mathbb {N}$ and $m \in \mathbb {R}$. For $p \geqslant 1$ a real valued, *p*–homogeneous *super-action preserving symbol* of order *m* is a symbol $\texttt{m}_p^{({\texttt{SAP}})}(Z;\xi )$ in $\widetilde{\Gamma }^m_p$, independent of *x*, of the form

$$\begin{aligned} \texttt{m}_p^{(\texttt{SAP})}(Z;\xi )= \sum _{({\vec {\jmath }_p},{\vec {\sigma }_p})\in {\mathfrak {S}}_p } M_{\vec {\jmath }_p}^{\vec {\sigma }_p}(\xi ) z_{\vec {\jmath }_p}^{\vec {\sigma }_p } \, . \end{aligned}$$

For $p = 0$ we say that any symbol in $\widetilde{\Gamma }^m_0$ is super-action preserving. A pluri-homogeneous super-action preserving symbol is a finite sum of homogeneous super-action preserving symbols.

#### Remark 7.7

A super-action preserving symbol has even degree *p* of homogeneity. Indeed, if $z_{\vec {\jmath }_p}^{\vec {\sigma }_p}$ is super- action preserving then $(\alpha ,\beta )$ defined in (7.4) satisfies $|\alpha | = |\beta |$ and $p= |\alpha +\beta | = 2| \alpha |$ is even.

Given a super-action preserving symbol we associate a super-action preserving Hamiltonian according to the following lemma (see Lemma 7.11 in [20]):

#### Lemma 7.8

Let $p \in \mathbb {N}$, $m \in \mathbb {R}$. If $(\texttt{m}^{({\texttt{SAP}})})_p(Z;\xi )$ is a *p*–homogeneous super-action preserving symbol in $\widetilde{\Gamma }^m_p$ according to Definition 7.6 then

$$\begin{aligned} H^{(\texttt{SAP})}_{p+2} (Z)\triangleq \text {Re}\left\langle {{{\,\mathrm{Op^{BW}}\,}}\left( (\texttt{m}^{({\texttt{SAP}})})_p(Z;\xi )\right) } z \ \bigg \vert \ \bar{z} \right\rangle _{\mathbb {R}} \end{aligned}$$

is a $(p+2)$–homogeneous super-action preserving Hamiltonian according to Definition 7.5.

### Birkhoff Normal Form Reduction

In this section we finally transform the system (7.2) into its Hamiltonian Birkhoff normal form, up to homogeneity *N*.

#### Proposition 7.9

(Hamiltonian Birkhoff normal form) Let $N \in \mathbb {N}$ and $0< \beta _1< \beta _2<4\left( 2+\sqrt{3} \right)$. Assume that the parameter $\beta = \frac{{\texttt{b}}^2}{\gamma } \in \left[ \beta _1,\beta _2 \right]$ is outside the zero measure set $\mathcal{B} \subset \left[ \beta _1,\beta _2 \right]$ defined in Proposition 2.7. Then, there exists ${\underline{\varrho }}={\underline{\varrho }}(N)>0$ such that, for any $\varrho \geqslant {\underline{\varrho }}$, for any $K \geqslant K'\triangleq \underline{K}' (\varrho )$ (defined in Proposition 6.1), there exists $\underline{s}_0 > 0$ such that, for any $s \geqslant \underline{s}_0$ there is ${\underline{\epsilon }}_0\triangleq {\underline{\epsilon }}_0(s)>0$ such that for all $0< \epsilon _0< {\underline{\epsilon }}_0(s)$ small enough, and any solution $U \in B_{\underline{s}_0}^K (I; \epsilon _0)\cap C^K_{*\mathbb {R}}(I; \dot{H}^{s}(\mathbb {T};\mathbb {C}^2))$ of the complex Kelvin-Helmholtz system (5.9), there exists a non–linear map $\mathcal {F}_{\texttt{nf}}(Z_0)$ such that

- **i** **Symplecticity:** $\mathcal {F}_{\texttt{nf}}(Z_0)$ is symplectic up to homogeneity *N* (Definition A.11);
- **ii** **Conjugation:** If $Z_0$ solves the system (7.2) then the variable $Z\triangleq \mathcal {F}_{\texttt{nf}}(Z_0)$ solves the *Hamiltonian system up to homogeneity N* (cfr. Definition A.10) 7.11 where
  - $H^{(\texttt{SAP})}_\frac{3}{2}(Z)$ is the super-action preserving Hamiltonian  with a pluri-homogeneous super-action preserving symbol  in $\Sigma _2^N \widetilde{\Gamma }_q^{\frac{3}{2}}$, according to Definition 7.6;
  - ${\boldsymbol{J}}_\mathbb {C}\nabla H^{(\texttt{SAP})}_{-\varrho }(Z)$ is a super-action preserving, Hamiltonian, smoothing vector field in $\Sigma _{3}^{N+1} \widetilde{{\mathfrak X }}^{-\varrho + {\underline{\varrho }}}_q$ (see 7.5);
  is a non-homogeneous symbol in $\Gamma ^{\frac{3}{2}}_{K,\underline{K}',N+1}[\epsilon _0]$ with imaginary part  in $\Gamma ^{0}_{K,\underline{K}',N+1}[\epsilon _0]$;
  - $\pmb {R}_{>N}(U;t)$ is a real-to-real matrix of non-homogeneous smoothing operators in $\left( \mathcal {R}^{-\varrho + {\underline{\varrho }}}_{K,\underline{K}',N+1}[\epsilon _0] \right) ^{2\times 2}$.
- **iii** **Boundedness:** There exists a constant $C\triangleq C_{s,K}>0$ such that for all $0\leqslant k\leqslant K$ and any $Z_0\in B_{\underline{s}_0}^K(I;\epsilon _0)\cap C^{K}_{*\mathbb {R}}(I;\dot{H}^{s}(\mathbb {T}, \mathbb {C}^2))$ one has
  7.12 $$\begin{aligned} C^{-1} \Vert Z_0 \Vert _{k,s}\leqslant \Vert \mathcal {F}_{\texttt{nf}}(Z_0) \Vert _{k,s} \leqslant C\Vert Z_0 \Vert _{k,s} \end{aligned}$$
  and
  7.13 $$\begin{aligned} {C}^{-1} \Vert U(t) \Vert _{\dot{H}^s} \leqslant \Vert Z (t) \Vert _{\dot{H}^s} \leqslant C \Vert U(t) \Vert _{\dot{H}^s} \, , \quad \forall t \in I. \end{aligned}$$

#### Notation 7.10

From now on we denote with $\boldsymbol{R}_p^\texttt{H}$ any smoothing remainder in $\widetilde{ \mathcal {R}}^{-\varrho }_p$ such that $\boldsymbol{R}_p^\texttt{H}(Z)Z$ is a $p+1$-homogeneous Hamiltonian vector field, namely $\boldsymbol{R}_p^\texttt{H}(Z)Z= \boldsymbol{J}_\mathbb {C}\nabla H_{p+2}(Z)$ for some $p+2$ homogeneous Hamiltonian as per DefinitionA.8.

#### Proof of Proposition 7.9

The proof consists in *N* steps and is analogous to the proof of Proposition 7.12 in [20]. For completeness, we only sketch the main steps and we refer to [20] for a detailed proof.

We first reduce the quadratic terms of the vector field in (7.2).

**Step **1**: Elimination of the quadratic smoothing remainder in equation** (7.2).

The *x*-independent symbol  in (7.2) belongs to $\Sigma _2^N \widetilde{\Gamma }^{\frac{3}{2}}_q$ and the only quadratic component of the vector field in (7.2) is $\boldsymbol{R}_1^\texttt{H}(Z_0)Z_0$ where (recall the notation in (3.18))

7.14 $$\begin{aligned} \boldsymbol{R}_1^\texttt{H}(Z_0)\triangleq \mathcal {P}_1[{\boldsymbol{R}}_{\leqslant N}(Z_0)] \in \left( \widetilde{\mathcal {R}}_1^{-\varrho +\varrho (N)} \right) ^{2\times 2}. \end{aligned}$$

Since the system (7.2) is Hamiltonian up to homogeneity *N*, $\boldsymbol{R}_1^\texttt{H}(Z_0)Z_0$ is a Hamiltonian vector field that we expand in Fourier coordinates as (recall (3.19))

7.15 $$\begin{aligned} \big (\boldsymbol{R}_1^\texttt{H}(Z_0)Z_0\big )_k^\sigma = \sum _{ \begin{array}{c} (j_1, j_2, k, \sigma _1, \sigma _2, - \sigma ) \in \mathfrak T_3 \end{array} } X_{j_1,j_2,k}^{\sigma _1,\sigma _2,\sigma } (z_0)_{j_1}^{\sigma _1} (z_0)_{j_2}^{\sigma _2}. \end{aligned}$$

In order to remove $\boldsymbol{R}_1^\texttt{H}(Z_0) Z_0$ from (7.2), we perform the change of variable s $Z_1 = \texttt{F}^{(1)}_{\leqslant N}(Z_0)$ where $\texttt{F}^{(1)}_{\leqslant N}(Z_0)$ is the map given by Lemma A.17 relative to a Hamiltonian smoothing vector field

7.16 $$\begin{aligned} \big ({\boldsymbol{G}}_1^\texttt{H}(Z_0)Z_0\big )_k^\sigma = \sum _{ \begin{array}{c} (j_1, j_2, k, \sigma _1, \sigma _2, - \sigma ) \in {\mathfrak {T}}_3 \end{array}}G_{j_1,j_2,k}^{\sigma _1,\sigma _2,\sigma } (z_0)_{j_1}^{\sigma _1} (z_0)_{j_2}^{\sigma _2}, \quad \boldsymbol{G}_1^\texttt{H}(Z_0)\in \left( \mathcal {R}^{-\varrho '}_1 \right) ^{2\times 2}\nonumber \\ \end{aligned}$$

for some $\varrho '>0$ to be determined. Since $\boldsymbol{G}_1^\texttt{H}(Z)Z$ is a Hamiltonian vector field, Lemma A.17 implies, by construction, that $\texttt{F}^{(1)}_{\leqslant N}$ is symplectic up to homogeneity *N* and it has the form

7.17 $$\begin{aligned} Z_1 \triangleq \texttt{F}^{(1)}_{\leqslant N}(Z_0) = Z_0 + {\boldsymbol{G}}_1^\texttt{H}(Z_0)Z_0+ \boldsymbol{F}_{\geqslant 2}(Z_0) Z_0 , \qquad \boldsymbol{F}_{\geqslant 2}(Z_0) \in \left( \Sigma _{2}^N \widetilde{\mathcal {R}}^{-\varrho '}_q \right) ^{2\times 2}.\nonumber \\ \end{aligned}$$

Applying Lemma A.12, we obtain that the variable $Z_1$ solves a system which is Hamiltonian up to homogeneity *N*. We compute it using Lemma A.20. Its assumption **(A)** at page 66 holds since $Z_0$ solves (7.2). Then Lemma A.20 implies that the variable $Z_1$ solves7.18where

$\bullet$  is a real valued symbol, independent of *x*, in $\Sigma _{2}^N \widetilde{\Gamma }_q^{\frac{3}{2}}$;

$\bullet$  is a non-homogeneous real valued symbol, independent of *x*, in $\Gamma ^\frac{3}{2}_{K,\underline{K}',N+1}[\epsilon _0]$ with imaginary part  in $\Gamma ^0_{K,\underline{K}', N+1}[\epsilon _0]$;

$\bullet$ $\boldsymbol{R}_1^\texttt{H}(Z_1)$ is defined in (7.14) and $\boldsymbol{G}_1^+(Z_1)Z_1\in \widetilde{{\mathfrak X }}^{-\varrho '+\frac{3}{2}}_{2}$ has Fourier expansion, by (B.15) and (7.16),

7.19 $$\begin{aligned} (\boldsymbol{G}^+_1(Z_1)Z_1)_{k}^\sigma =\!\!\! \!\!\! \!\!\! \sum _{ (j_1, j_2, k, \sigma _1, \sigma _2, - \sigma ) \in {\mathfrak {T}}_3 } \!\!\! \!\!\! \!\!\! \textrm{i}\big (\sigma _1\omega _{\gamma ,{\texttt{b}}}(j_1)+\sigma _2 \omega _{\gamma ,{\texttt{b}}}(j_2)- \sigma \omega _{\gamma ,{\texttt{b}}}(k)\big )G_{j_1,j_2,k}^{\sigma _1,\sigma _2,\sigma } (z_1)_{j_1}^{\sigma _1} (z_1)_{j_2}^{\sigma _2};\nonumber \\ \end{aligned}$$

$\bullet$ $\boldsymbol{R}_{\geqslant 2}^+(Z_1)$ is a matrix of pluri-homogeneous smoothing operators in $\left( \Sigma _2^N \tilde{\mathcal {R}}^{-\varrho + {\underline{\varrho }}(2)}_q \right) ^{2\times 2}$ where

7.20 $$\begin{aligned} -\varrho +{\underline{\varrho }}(2) \triangleq -\varrho '+\frac{3}{2} \ ; \end{aligned}$$

$\bullet$ $\boldsymbol{R}_{>N}^+(U;t)$ is a matrix of non-homogeneous smoothing operators in $\left( \mathcal {R}^{-\varrho + {\underline{\varrho }}(2)}_{K,\underline{K}',N+1}[\epsilon _0] \right) ^{2\times 2}$.

By (7.15), (7.19) we solve

7.21 $$\begin{aligned} \boldsymbol{R}_1^\texttt{H}(Z_1)Z_1 + \boldsymbol{G}_1^+(Z_1)Z_1 = 0 \end{aligned}$$

by taking

7.22 $$\begin{aligned} G_{j_1,j_2,k}^{\sigma _1,\sigma _2,\sigma }\triangleq {\left\{ \begin{array}{ll} 0, &  \text{ if } \ (j_1,j_2,k,\sigma _1,\sigma _2,-\sigma ) \notin {\mathfrak {T}}_3, \\ \displaystyle - {\frac{ X_{j_1,j_2,k}^{\sigma _1,\sigma _2,\sigma }}{\textrm{i}\big (\sigma _1\omega _{\gamma ,{\texttt{b}}}(j_1)+\sigma _2 \omega _{\gamma ,{\texttt{b}}}({j_2})- \sigma \omega _{\gamma ,{\texttt{b}}}({k})\big )}}, &  \text{ if } (j_1,j_2,k,\sigma _1,\sigma _2,-\sigma ) \in {\mathfrak {T}}_3 \, . \end{array}\right. }\nonumber \\ \end{aligned}$$

The previous expression and the fact that the vector field ${\boldsymbol{R}}_1^\texttt{H}(Z_0)Z_0$ is Hamiltonian justifies a posteriori that indeed ${\boldsymbol{G}}_1^\texttt{H}(Z_0)Z_0$ is Hamiltonian. Moreover, combining (7.14) and Proposition 2.7, taking $\beta \in \left[ \beta _1,\beta _2 \right] \setminus \mathcal {B}$, we get that ${\boldsymbol{G}}_1^\texttt{H}(Z_0)Z_0\in \widetilde{{\mathfrak X }}^{-\varrho '}_2$ with $\varrho ' \triangleq \varrho - \varrho (N) - \tau$. Thus, by Eq. (7.20), we get ${\underline{\varrho }}(2)=\varrho (N)+\tau +\frac{3}{2}$.

**Step** $p\geqslant 2$: We now assume that the system is in normal form up to degree $p-1$ of homogeneity. Next, we illustrate how to perform the normal form transformation on the terms of degree *p*. Specifically, we assume that $Z_{p-1}$ solves7.23where $\big (H^{(\texttt{SAP})}_{\frac{3}{2}}\big )_{\leqslant p+1}$, $\big (H^{(\texttt{SAP})}_{-\varrho }\big )_{\leqslant p+1}$ are pluri-homogeneous super-action preserving Hamiltonians,  and expands as7.24Moreover  and $\boldsymbol{R}_{\geqslant p}^\texttt{H}\in \Sigma _{p}^N \mathcal {R}_q^{-\varrho +\underline{\varrho }(p)}$. We reduce the homogeneous component

 into its resonant normal form. First of all we define the intermediate variable

7.25 $$\begin{aligned} W\triangleq \Phi _p(Z_{p-1})\triangleq \pmb {\mathcal {G}}_{g_p}^1(Z_{p-1})Z_{p-1}, \end{aligned}$$

where $\pmb {\mathcal {G}}_{g_p}^1(Z_{p-1})$ is the time 1-linear flow generated by ${{\,\mathrm{Op^{BW}_{vec}}\,}}\left( \textrm{i}g_p\right)$, where $g_p$ is the Fourier multiplier

7.26 $$\begin{aligned} g_p(Z_{p-1};\xi )\triangleq \sum \limits _{(\vec {\jmath }_p, \vec {\sigma }_p) \in {\mathfrak {T}}_p} \texttt{G}_{\vec {\jmath }_p}^{\vec {\sigma }_p}(\xi )(z_{p-1})_{\vec {\jmath }_p}^{\vec {\sigma }_p} \in \widetilde{\Gamma }_p^{\frac{3}{2}}. \end{aligned}$$

Then Lemma A.19 implies that the variable *W* defined in (7.25) solves7.27where $g_p^+$ expands as

7.28 $$\begin{aligned} g^+_p(W;\xi )= \sum _{ (\vec {\jmath }_p, \vec {\sigma }_p) \in {\mathfrak {T}}_p}&\textrm{i}\mathbf {\sigma }_p \cdot \vec {\omega }_{\gamma ,{\texttt{b}}}(\vec {\jmath }_p)\texttt{G}_{\vec {\jmath }_p}^{\vec {\sigma }_p}(\xi )w_{\vec {\jmath }_p}^{\vec {\sigma }_p}, \end{aligned}$$

while $\boldsymbol{R}_{\geqslant p}\in \left( \Sigma _1^N \tilde{\mathcal {R}}^{-\varrho +\underline{\varrho }(p)+c(N,p)}_q \right) ^{2\times 2}$, $\boldsymbol{R}_{>N}\in \left( \mathcal {R}^{-\varrho +\underline{\varrho }(p)+c(N,p)}_{K,K',N+1}[\epsilon _0] \right) ^{2\times 2}$ for some $c(N,p)>0$. In order to get a *p*-homogeneous super-action preserving normal form symbol, we solve the homological equationwhere $\mathfrak {S}_p$ has been introduced in (7.5). One solution is obtained by choosing

7.29 $$\begin{aligned} \texttt{G}_{\vec {\jmath }_p}^{\vec {\sigma }_p}(\xi ) \triangleq {\left\{ \begin{array}{ll} 0, &  \text{ if } (\vec {\jmath }_p, \vec {\sigma }_p)\in \mathfrak {S}_p,\\ \displaystyle {\frac{\texttt{D}_{\vec {\jmath }_p}^{\vec {\sigma }_p}(\xi )}{\textrm{i}\mathbf {\sigma }_p \cdot \vec {\omega }_{\gamma ,{\texttt{b}}}(\vec {\jmath }_p)}}, &  \text{ if } (\vec {\jmath }_p, \vec {\sigma }_p)\not \in \mathfrak {S}_p . \end{array}\right. } \end{aligned}$$

The previous expression (7.29) and the fact that  is real valued justifies a posteriori that indeed $g_p$ is real valued. Moreover, combining estimate (3.5) for  and Proposition 2.7, taking $\beta \in \left[ \beta _1,\beta _2 \right] \setminus \mathcal {B}$, we get that $g_p$ satisfies the corresponding estimate (3.5) with $\mu \leadsto \mu +\tau$. Now, we observe that, by Lemma A.13,7.30with Hamiltonian7.31which is super-action preserving by Lemma 7.8, and a matrix of smoothing operators $\boldsymbol{R}_p(W)$ in $\left( \widetilde{\mathcal {R}}^{-\varrho '}_p \right) ^{2\times 2}$ for any $\varrho ' \geqslant 0$. Therefore (7.27) becomes7.32where (see (7.23),(7.31))

7.33 $$\begin{aligned} \big (H^{(\texttt{SAP})}_{\frac{3}{2}}\big )_{\leqslant p+2}\triangleq \big (H^{(\texttt{SAP})}_{\frac{3}{2}}\big )_{\leqslant p+1} + \big (H_{\frac{3}{2}}^{(\texttt{SAP})}\big )_{p+2}, \end{aligned}$$

while $\boldsymbol{R}_{\geqslant p}$ is a new smoothing remainder in $\left( \Sigma _1^N \tilde{\mathcal {R}}^{-\varrho +\underline{\varrho }(p)+c(N,p)}_q \right) ^{2\times 2}$. Note that the new system (7.32) is not Hamiltonian up to homogeneity *N* (unlike system (7.23) for $Z_{p-1}$), since the map $\Phi _p(Z_{p-1}) = \pmb {\mathcal {G}}^1_{g_p}(Z_{p-1}) Z_{p-1}$ in (7.25) is not symplectic up to homogeneity *N*. By Lemma A.7 we only know that $\pmb {\mathcal {G}}^1_{g_p}(Z_{p-1})$ is linearly symplectic. We now apply Theorem A.16 to find a correction of $\Phi _p(Z_{p-1})$ which is symplectic up to homogeneity *N*. The assumptions of Theorem A.16 are verified applying Lemma A.7, therefore there exists $\boldsymbol{R}_{\leqslant N}^{(p)} \in \left( \Sigma _p^N \widetilde{\mathcal {R}}^{-\varrho -\frac{3}{2}}_q\right) ^{2\times 2}$ such that the variable

7.34 $$\begin{aligned} V \triangleq \mathcal {C}_N^{(p)}(W)\triangleq \big ( \textrm{Id}+ \boldsymbol{R}_{\leqslant N}^{(p)}(W) \big ) W = \big ( \textrm{Id}+ \boldsymbol{R}_{\leqslant N}^{(p)}(\Phi _p(Z_{p-1})) \big ) \Phi _p(Z_{p-1})\nonumber \\ \end{aligned}$$

is symplectic up to homogeneity *N*, thus solves a system which is Hamiltonian up to homogeneity *N*. Since *W* solves (7.32) then Lemma A.20 implies that $V =W + {\boldsymbol{R}}_{\leqslant N}^{(p)}(W)W$ solves7.35where , $\boldsymbol{J}_\mathbb {C}\nabla \left( H_{-\varrho }\right) _{p+2}\in \mathfrak {X}^{-\varrho +\underline{\varrho }(p)+ C(N,p)}_p$, $\boldsymbol{R}_{\geqslant p+1}\in \Sigma _{p}^N \widetilde{\mathcal {R}}^{-\varrho +\underline{\varrho }(p)+ C(N,p)}$. Notice that the Hamiltonian structure of $\boldsymbol{J}_\mathbb {C}\nabla \left( H_{-\varrho }\right) _p(V)$ is justified a posteriori by the fact that the *p*-homogeneous component of the vector field in (7.35) is Hamiltonian and the term in its first line is Hamiltonian, thus we deduce the Hamiltonianity by difference. Thus we can proceed as in Step 1. First we Fourier expand $\boldsymbol{J}_\mathbb {C}\nabla \left( H_{-\varrho }\right) _{p+2}$ as

7.36 $$\begin{aligned} \big ( \boldsymbol{J}_\mathbb {C}\nabla \left( H_{-\varrho }\right) _{p+2}(V)\big )_k^\sigma = \!\!\!\!\!\!\!\!\!\!\!\!\!\!\! \sum _{ (\vec {\jmath }_{p+1}, k, \vec {\sigma }_{p+1}, - \sigma ) \in {\mathfrak {T}}_{p+2}} \!\!\!\!\!\!\!\!\!\!\!\!\!\! X_{\vec {\jmath }_{p+1},k}^{\vec {\sigma }_{p+1},\sigma } v_{\vec {\jmath }_{p+1}}^{\vec {\sigma }_{p+1}} \, . \end{aligned}$$

Then we use Lemma A.17 to generate a symplectic up to homogeneity *N* map $\mathcal {F}_{\leqslant N}^{(p)}$ associate to the Hamiltonian smoothing vector field

7.37 $$\begin{aligned} \big (\boldsymbol{G}_p(V)V\big )_k^\sigma = \!\!\!\!\!\!\!\!\! \sum _{ (\vec {\jmath }_{p+1}, k, \vec {\sigma }_{p+1}, - \sigma ) \in {\mathfrak {T}}_{p+2}} \!\!\!\!\!\!\!\! G_{\vec {\jmath }_{p+1},k}^{\mathbf {\sigma }_{p+1},\sigma } v_{\vec {\jmath }_{p+1}}^{\vec {\sigma }_{p+1}}. \end{aligned}$$

Applying Lemma A.20 the analogue of the homological equation (7.21) is

7.38 $$\begin{aligned}&\boldsymbol{J}_\mathbb {C}\nabla \left( H_{-\varrho }\right) _{p+2}(Z_p) + \boldsymbol{G}_p^+(Z_p)Z_p = {\boldsymbol{J}}_\mathbb {C}\nabla \left( H^{(\texttt{SAP})}_{-\varrho }\right) _p(Z_p)\nonumber \\&\quad \triangleq \!\!\!\sum _{{(\mathbf {\jmath }_{p+1}, k, \mathbf {\sigma }_{p+1}, - \sigma ) \in {\mathfrak {S}}_{p+2}} } \!\!\!\!\!\!\!\!\!\!\! X_{\vec {\jmath }_{p+1},k}^{\vec {\sigma }_{p+1}, \sigma } \, (z_p)_{\vec {\jmath }_{p+1}}^{\vec {\sigma }_{p+1}} \end{aligned}$$

where the vector field $\boldsymbol{G}_p^+(Z_p)Z_p$ is explicitly given by

$$\begin{aligned} (\boldsymbol{G}_p^+(Z_p)Z_p)_k^\sigma \triangleq \!\!\!\!\!\!\!\!\!\!\!\! \sum _{(\vec {\jmath }_{p+1}, k, \vec {\sigma }_{p+1}, - \sigma ) \in {\mathfrak {T}}_{p+2}} \!\!\!\!\!\!\!\!\!\!\!\!\!\!\!\!\!\!\!\!\!\!\! \textrm{i}\big (\vec {\sigma }_{p+1}\cdot \vec {\omega }_{\gamma ,{\texttt{b}}}(\vec {\jmath }_{p+1})- \sigma \omega _{\gamma ,{\texttt{b}}}(k)\big )G_{\vec {\jmath }_{p+1},k}^{\vec {\sigma }_{p+1},\sigma } (z_p)_{\vec {\jmath }_{p+1}}^{\vec {\sigma }_{p+1}} \ . \end{aligned}$$

Then a solution of (7.38) is given by

7.39 $$\begin{aligned} G_{\vec {\jmath }_{p+1},k}^{\vec {\sigma }_{p+1},\sigma } \triangleq {\left\{ \begin{array}{ll} 0 ,&  \text{ if } (\mathbf {\jmath }_{p+1}, k, \mathbf {\sigma }_{p+1}, - \sigma )\in \mathfrak {S}_{p+2},\\ -\displaystyle {\frac{ X_{\vec {\jmath }_{p+1},k}^{\vec {\sigma }_{p+1}, \sigma } }{\textrm{i}(\vec {\sigma }_{p+1}\cdot \vec {\omega }_{\gamma ,{\texttt{b}}}(\vec {\jmath }_{p+1})- \sigma \omega _{\gamma ,{\texttt{b}}}(k))}} ,&  \text{ if } (\mathbf {\jmath }_{p+1}, k, \mathbf {\sigma }_{p+1}, - \sigma ) \not \in \mathfrak {S}_{p+2} . \end{array}\right. } \end{aligned}$$

The previous expression and the fact that the vector field $\boldsymbol{J}_\mathbb {C}\nabla \left( H_{-\varrho }\right) _{p+2}$ is Hamiltonian justifies a posteriori that indeed $G_p(Z_p)Z_p$ is Hamiltonian. Moreover, since $\boldsymbol{J}_\mathbb {C}\nabla \left( H_{-\varrho }\right) _{p+2}$ is $\varrho _p$-smoothing, we apply Proposition 2.7, taking $\beta \in \left[ \beta _1,\beta _2 \right] \setminus \mathcal {B}$ and we get that $\boldsymbol{G}_p(Z_p)Z_p\in \widetilde{{\mathfrak X }}^{-\varrho _{p+1}}_2$ with $\varrho _{p+1}\triangleq \varrho _p - c(N) - \tau .$ $\square$

### Energy Estimate and Proof of the Main Theorem

#### Remark 7.11

We can derive local existence for (5.9) following the computations in the main result of [71] and exploiting the fact that the local existence result of [71] uses the very same paradifferential functional setting as in the present manuscript. Minor modifications in the works [14, 18] can also provide local existence for the setting of (1.4).

The following result, analogous to [68, Lemma 6.3], enables to bound the norms $\Vert \partial _t^k U(t) \Vert _{s-\frac{3}{2} k}$ of the time derivatives of a solution *U*(*t*) of (5.9) by $\Vert U(t)\Vert _s$.

#### Lemma 7.12

Let $K\in \mathbb {N}$. There exists $s_0> 0$ such that for any $s\geqslant s_0$, any $\epsilon \in \left( 0, \overline{\epsilon _0}\left( s \right) \right)$ small, if *U* belongs to $B^0_{s_0, \mathbb {R}}\left( I;\epsilon \right) \cap C^{0}_*\left( I; \dot{H}^{s}\left( {\mathbb {T}};\mathbb {C}^2 \right) \right)$ and solves (5.2) then $U\in C^K_{* \mathbb {R}}\left( I; \dot{H}^s(\mathbb {T};\mathbb {C}^2)\right)$ and there exists $C_1 \triangleq C_1\left( s, K \right) \geqslant 1$ such that

$$\begin{aligned}\left\| U\left( t \right) \right\| _s \leqslant \left\| U\left( t \right) \right\| _{K, s} \leqslant C_1 \left\| U\left( t \right) \right\| _s,\qquad \forall t \in I.\end{aligned}$$

#### Proof

The proof is inductive and follows the same line of [68, Lemma 6.3], so we sketch only the first step. As $\Vert U(t)\Vert _s\leqslant \Vert U\left( t \right) \Vert _{K,s}$ is obvious in view of the definition (3.3), it remains to prove the second inequality. We start by estimating $\Vert \partial _t U(t)\Vert _{s-\frac{3}{2}}$. Using equation (5.2) for *U*(*t*) and then (3.13) with $k=K'=p=0$, $m=\frac{3}{2}$ and estimate (3.16) with $m\leadsto -\varrho$ and $k=0$, we get

$$\begin{aligned} \Vert \partial _t U(t)\Vert _{s-\frac{3}{2}}&\leqslant \left\| {{{\,\mathrm{Op^{BW}}\,}}\left( {\boldsymbol{A}}_{\frac{3}{2}}\left( U;x \right) \ \omega _{\gamma , {\texttt{b}}}\left( \xi \right) + {\boldsymbol{A}}_{1}\left( U;x, \xi \right) + {\boldsymbol{A}}_{\frac{1}{2}}\left( U;x \right) \ \left| \xi \right| ^{\frac{1}{2}} + {\boldsymbol{A}}_{\left[ 0 \right] } \left( U;x, \xi \right) \right) } U\right\| _{s-\frac{3}{2}} \\&\quad +\left\| {\boldsymbol{R}}\left( U \right) U\right\| _{s-\frac{3}{2}} \\&\lesssim \Vert U(t)\Vert _{s}. \end{aligned}$$

The estimates for $k \geqslant 2$ follow in a similar manner, additionally using estimates (3.13) and (3.16) with $k \geqslant 1$ to handle higher-order derivatives.

$$\square $$

We fix now the parameters appearing in Proposition 7.9. In its statement, we set $\varrho \triangleq \underline{\varrho }(N)$ and $K \triangleq \underline{K'}(\varrho )$, which implies the existence of the constant $\underline{s_0} > 0$ which we increase, if necessary, to fit the range of Lemma 7.12, such that for any $s \geqslant \underline{s_0}$, and any fixed $0 < \epsilon _0 \leqslant \min \{ \overline{\epsilon _0}(s), \underline{\epsilon _0}(s) \}$, where $\underline{\epsilon _0}(s)$ is defined in Proposition 7.9, and $\overline{\epsilon _0}(s)$ in Lemma 7.12, the conclusions of Proposition 7.9 and Lemma 7.12 hold. Therefore, one can obtain the following energy estimate. Notice that the time-reversibility of the Kelvin-Helmholtz system allows us to restrict the discussion to positive times $t>0.$

#### Lemma 7.13

(Energy estimate) Let *U*(*t*) be a solution of equation (5.9) in $B^{K}_{\underline{s_0}}\left( I;\epsilon _0 \right) \cap C^{K}_*\left( I; \dot{H}^{s}\left( {\mathbb {T}};\mathbb {C}^2 \right) \right)$. Then there exists $\bar{C}_2 \left( s \right) > 1$ such that

7.40 $$\begin{aligned} \left\| U\left( t \right) \right\| _s^2 \leqslant \bar{C}_2 \left( s \right) \left( \left\| U\left( 0 \right) \right\| _s^2 + \int _0^t\left\| U\left( \tau \right) \right\| _{s_0}^{N+1}\left\| U\left( \tau \right) \right\| _s^2 \text {d}\tau \right) , \quad \forall 0< t < T. \end{aligned}$$

#### Proof

The variable *Z* defined in Proposition 7.9 solves the normal form Equation (7.11). Then, denoting by $H^{(\texttt{SAP})}\triangleq H^{(\texttt{SAP})}_{\frac{3}{2}}+H^{(\texttt{SAP})}_{-\varrho }$ we have thatwhere we used Lemma 7.4 and the fact that  is in $\Gamma ^0_{K, K', N+1}[\epsilon _0]$ and ${\boldsymbol{R}}_{>N}\left( U;t \right)$ is in $\mathcal {M}^{0}_{K, K', N+1}[\epsilon _0]$. Integrating in time the above inequality, then using Eq. (7.13) and 7.12, we obtain (7.40). $\square$

The energy estimate (7.40) and the equivalence

7.41 $$\begin{aligned} \left\| \eta \left( t \right) \right\| _{H^{s+\frac{1}{4}}_0} + \left\| \psi \left( t \right) \right\| _{\dot{H} ^{s-\frac{1}{4}}} \sim \left\| U\left( t \right) \right\| _s, \end{aligned}$$

which is a consequence of (5.8), imply, by a standard bootstrap argument, Theorem 1.1. For detailed computations we refer the interested reader to [20, Section 8] or [68, Section 6.4].

## Data Availability

No data has been used in this work.

## Hamiltonian Formalism

In this appendix we establish the Hamiltonian formalism for the problem at hand, following [20, Section 3].

### Linearly Hamiltonian Systems

In this section, we provide the linear Hamiltonian framework needed for our analysis. The linear Hamiltonian structure is the natural algebraic property that emerges from the paralinearization of a Hamiltonian system.

#### Definition A.1

*(Linearly Hamiltonian paradifferential operator)* A real-to-real matrix of paradifferential complex operators is *linearly Hamiltonian* if it is of the form

A.1 $$\begin{aligned}&{\boldsymbol{J}}_{\mathbb {C}}{{{\,\mathrm{Op^{BW}}\,}}\left( {\boldsymbol{B}}\right) },\qquad {\boldsymbol{B}}\triangleq \left[ \begin{array}{cc} b_1\left( U;t,x,\xi \right) &  b_2\left( U;t,x,\xi \right) \\ \overline{b_2\left( U;t,x,-\xi \right) } &  \overline{b_1\left( U;t,x,-\xi \right) } \end{array} \right] ,\nonumber \\&\left\{ \begin{aligned}&b_1\left( U;t,x,-\xi \right) = b_1\left( U;t,x,\xi \right) , \\&b_2\left( U;t,x,\xi \right) \in {\mathbb {R}}. \end{aligned} \right. \end{aligned}$$

In other words, $b_1$ is even in $\xi$ and $b_2$ is real valued. This is equivalent to require that the matrix valued paradifferential operator ${{{\,\mathrm{Op^{BW}}\,}}\left( {\boldsymbol{B}}\right) }$ is symmetric.

#### Definition A.2

*(Linearly Hamiltonian operator up to homogeneity* *N**)* A real-to-real matrix of spectrally localized maps ${\boldsymbol{J}}_{\mathbb {C}} \textbf{B} (U;t)$ in $\left( \Sigma \mathcal{S}_{K,K',p}[\epsilon _0,N] \right) ^{2\times 2}$ is *linearly Hamiltonian up to homogeneity N* if its pluri-homogeneous component $\mathcal {P}_{\leqslant N} (\textbf{B} (U;t))$ (defined through (3.18)) is symmetric, namely

$$\begin{aligned} \mathcal {P}_{\leqslant N} (\textbf{B}(U;t)) = \mathcal {P}_{\leqslant N} \left( \textbf{B}(U;t)^\intercal \right) . \end{aligned}$$

In particular, a real-to-real matrix of paradifferential operators is linearly Hamiltonian up to homogeneity *N* if it has the form (cfr. (A.1))

A.2 $$\begin{aligned}&{\boldsymbol{J}}_{\mathbb {C}} {{{\,\mathrm{Op^{BW}}\,}}\left( \begin{matrix} b_1(U;t, x,\xi ) &  b_2(U; t, x, \xi ) \\ \overline{b_2(U;t, x, -\xi )} &  \overline{b_1(U; t, x, -\xi )} \end{matrix}\right) }, \nonumber \\&\ {\left\{ \begin{array}{ll} b_1(U;t, x, - \xi ) - b_1(U;t, x, \xi ) \in \Gamma ^m_{K,K',N+1}[\epsilon _0], \\ \text {Im}b_2(U;t, x,\xi ) \in \Gamma ^{m'}_{K,K',N+1}[\epsilon _0] \end{array}\right. } \end{aligned}$$

for some $$m, m' $$ in $$ \mathbb {R}$$.

#### Definition A.3

*(Linearly symplectic map)* A real-to-real linear transformation $\mathcal {A}$ is linearly symplectic if $\mathcal {A}^* \Omega _{\mathbb {C}} = \Omega _{\mathbb {C}}$, where $\Omega _{\mathbb {C}}$ is defined as

A.3 $$\begin{aligned} \Omega _{\mathbb {C}} \left( \begin{bmatrix} u_1 \\ \bar{u}_1 \end{bmatrix} , \begin{bmatrix} u_2 \\ \bar{u}_2 \end{bmatrix} \right) \triangleq \left\langle {\boldsymbol{E}}_{\mathbb {C}} \begin{bmatrix} u_1 \\ \bar{u}_1 \end{bmatrix} \ \bigg \vert \ \begin{bmatrix} u_2 \\ \bar{u}_2 \end{bmatrix} \right\rangle _{{\mathbb {R}}}, \qquad {\boldsymbol{E}}_{\mathbb {C}}\triangleq {\boldsymbol{J}}_{\mathbb {C}}^{-1}, \end{aligned}$$

namely

$$\begin{aligned}\mathcal {A}^\top {\boldsymbol{E}}_{\mathbb {C}}\mathcal {A}= {\boldsymbol{E}}_{\mathbb {C}}.\end{aligned}$$

#### Definition A.4

*(Linearly symplectic map up to homogeneity* *N**)* A real-to-real matrix of spectrally localized maps $\textbf{S}(U;t)$ in $\left( \Sigma \mathcal {S}_{K,K',0}[r,N] \right) ^{2\times 2}$ is *linearly symplectic up to homogeneity N* if

A.4 $$\begin{aligned} \textbf{S}(U;t)^\top \, {\boldsymbol{E}}_\mathbb {C}\, \textbf{S}(U;t)= {\boldsymbol{E}}_\mathbb {C}+ S_{>N}(U;t) , \end{aligned}$$

where $$ {\boldsymbol{E}}_\textbf{C}$$ is the symplectic operator defined in (A.3) and $$S_{>N}(U;t) $$ is a matrix of spectrally localized maps in $$ \left( \mathcal {S}_{K,K',N+1}[\epsilon _0] \right) ^{2\times 2}$$.

Linearly symplectic maps up to homogeneity *N* preserve the linear Hamiltonian structure up to homogeneity *N*. The following result is borrowed from [20, Lemma 3.9].

#### Lemma A.5

Let ${\boldsymbol{J}}_\mathbb {C}{\boldsymbol{B}}(U;t)$ be a linearly Hamiltonian operator up to homogeneity *N* (Definition A.2) and ${\boldsymbol{S}}(U;t)$ be an invertible map, linearly symplectic to homogeneity *N* (Definition A.4). Then the operators ${\boldsymbol{S}}(U;t) {\boldsymbol{J}}_\mathbb {C}{\boldsymbol{B}}(U;t) \boldsymbol{S}(U;t)^{-1}$ and $(\partial _t{\boldsymbol{S}}(U;t)) {\boldsymbol{S}}^{-1}(U;t)$ are linearly Hamiltonian up to homogeneity *N*.

We consider the flow of a linearly Hamiltonian up to homogeneity *N* paradifferential operator. The following is Lemma 3.16 of [20].

#### Lemma A.6

(Linear symplectic flow) Let $p \in \mathbb {N}$, $N, K,K' \in \mathbb {N}$ with $K'\leqslant K$, $m \leqslant 1$, $r>0$. Let

$$\begin{aligned}{\boldsymbol{J}}_\mathbb {C}{{{\,\mathrm{Op^{BW}}\,}}\left( {\boldsymbol{B}}\right) }={{{\,\mathrm{Op^{BW}}\,}}\left( \begin{bmatrix} \overline{ \textrm{i}b_2^\vee } &  \overline{\textrm{i}b_1^\vee }\\ \textrm{i}b_1 &  \textrm{i}b_2 \end{bmatrix}\right) }\end{aligned}$$

be a linearly Hamiltonian operator up to homogeneity *N* (Definition A.2) where ${\boldsymbol{B}}$ is a matrix of symbols

$$\begin{aligned} {\boldsymbol{B}}\triangleq {\boldsymbol{B}}(\tau ,U; t, x, \xi )\triangleq \begin{pmatrix} b_1(\tau , U; t, x, \xi ) &  b_2(\tau ,U; t, x, \xi ) \\ \overline{b_2(\tau ,U; t, x, -\xi )} &  \overline{b_1(\tau ,U; t , x, -\xi )}\end{pmatrix}, \qquad {\left\{ \begin{array}{ll} b_1 \in \Sigma \Gamma ^{0}_{K,K',p}[r,N] , \\ b_2\in \Sigma \Gamma ^{m}_{K,K',p}[r,N] , \end{array}\right. } \end{aligned}$$

with $$b_1^\vee - b_1 $$ in $$ \Gamma ^0_{K,K',N+1}[\epsilon _0]$$ and the imaginary part $$ Im b_2 $$ in $$ \Gamma ^0_{K,K', N+1}[\epsilon _0]$$ (cfr. (A.2)) uniformly in $$ |\tau | \leqslant 1 $$. Then there exists $$ s_0 >0$$ such that, for any $$ U\in B_{s_0,\mathbb {R}}^{K}(I;r) $$, the system

has a unique solution $\pmb {\mathcal {G}}^{\tau }_{\boldsymbol{B}}(U)$ defined for all $| \tau | \leqslant 1$, satisfying the following properties:

(i) **Boundedness:** For any $s \in \mathbb {R}$ the linear map $\pmb {\mathcal {G}}^{\tau }_{{\boldsymbol{B}}}(U;t)$ is invertible and $\pmb {\mathcal {G}}_{{\boldsymbol{B}}}^\tau (U;t)$ and $\pmb {\mathcal {G}}_{{\boldsymbol{B}}}^\tau (U;t)^{-1}$ are non-homogeneous spectrally localized maps in $\left( \mathcal {S}_{K,K',0}^0 [\epsilon _0] \right) ^{2\times 2}$ according to Definition 3.20.
(ii) **Linear symplecticity:** The map $\pmb {\mathcal {G}}^\tau _{{\boldsymbol{B}}}(U;t)$ is linearly symplectic up to homogeneity *N* (Definition A.4). If ${\boldsymbol{J}}_\mathbb {C}{{{\,\mathrm{Op^{BW}}\,}}\left( \textbf{B}\right) }$ is linearly Hamiltonian (Definition A.1), then $\pmb {\mathcal {G}}^{\tau }_{{\boldsymbol{B}}}(U;t)$ is linearly symplectic (Definition A.3).
(iii) **Homogeneous expansion:** $\pmb {\mathcal {G}}_{{\boldsymbol{B}}}^\tau (U;t)$ and its inverse are spectrally localized maps and $\pmb {\mathcal {G}}_{{\pmb B}}^\tau (U;t)^{\pm }- \textrm{Id}$ belong to $\left( \Sigma \mathcal {S}_{K,K',p}^{(N+1) m_0}[r, N] \right) ^{2\times 2}$ with $m_0 \triangleq \max (m,0)$, uniformly in $|\tau | \leqslant 1$.

The flow generated by a Fourier multiplier satisfies similar properties. The following is Lemma 3.17 of [20]:

#### Lemma A.7

(Flow of a Fourier multiplier) Let $p \in \mathbb {N}$ and $g_p(Z;\xi )$ be a *p*–homogeneous, *x*-independent, real symbol in $\widetilde{\Gamma }^{\frac{3}{2}}_p$. Then, the flow $\pmb {\mathcal {G}}_{g_p}^\tau (Z)$ defined by

A.5 $$\begin{aligned} \partial _\tau \pmb {\mathcal {G}}_{g_p}^\tau (Z)= {{\,\mathrm{Op^{BW}_{vec}}\,}}\left( \textrm{i}g_p(Z;\xi )\right) \pmb {\mathcal {G}}_{g_p}^\tau (Z) \, , \qquad \pmb {\mathcal {G}}_{g_p}^0(Z)=\textrm{Id}\, , \end{aligned}$$

is well defined for any $|\tau | \leqslant 1$ and satisfies the following properties:

- (*i*) **Boundedness:** For any $K\in \mathbb {N}$ and $r>0$ the flow $\, \pmb {\mathcal {G}}^{\tau }_{g_p}(Z)$ and its inverse $\,\pmb {\mathcal {G}}^{-\tau }_{g_p}(Z)$ are real-to-real diagonal matrix of spectrally localized maps in $\left( \mathcal {S}_{K,0,0}^0[\epsilon _0] \right) ^{2\times 2}$.
- (*ii*) **Linear symplecticity:** The flow map $\pmb {\mathcal {G}}^\tau _{g_p}(Z)$ is linearly symplectic (Definition A.3).
- (*iii*) **Homogeneous expansion:** The flow map $\pmb {\mathcal {G}}^{\tau }_{g_p}(Z)$ and its inverse $\pmb {\mathcal {G}}^{-\tau }_{g_p}(Z)$ are matrices of spectrally localized maps such that $\pmb {\mathcal {G}}^{\pm \tau }_{g_p}(Z) -\textrm{Id}$ belong to $\left( \Sigma \mathcal {S}^{\frac{3}{2} (N+1)}_{K,0,p}[r,N] \right) ^{2\times 2}$, uniformly in $|\tau | \leqslant 1$.

### Non-linear Hamiltonian Systems

We first give the definition of *p*-homogeneous Hamiltonian functions.

#### Definition A.8

*(Homogeneous Hamiltonian)* Let $p \in \mathbb {N}$, a $p+2$-homogeneous Hamiltonian is a function defined on $\dot{H}^\infty (\mathbb {T};\mathbb {C}^2)$ with values in $\mathbb {C}$ which is real valued for any $U\in \dot{H}^{\infty }_\mathbb {R}(\mathbb {T};\mathbb {C}^2)$ of the form, c.f. (3.19)

A.6 $$\begin{aligned} H_{p+2}(U)= \langle M_p(U)U,U\rangle = \sum _{ (\mathbf {\jmath }, \mathbf {\sigma }) \in {\mathfrak {T}}_{p+2}} H_{\mathbf {\jmath }}^{\mathbf {\sigma }} \, u_{\mathbf {\jmath }}^{\mathbf {\sigma }},  &   M_p(U)\in \left( \widetilde{\mathcal {M}}_p^m \right) ^{2\times 2} \end{aligned}$$

for some $m\in \mathbb {R}$ and complex valued coefficients $H_{\mathbf {\jmath }}^{\mathbf {\sigma }}= H_{j_1,\ldots , j_{p+2}}^{\sigma _1,\ldots , \sigma _{p+2}}$. A pluri-homogeneous Hamiltonian is a polynomial of the form

A.7 $$\begin{aligned} H(U)= \sum _{p=0}^N H_{p+2}(U), \end{aligned}$$

where, for $p=0, \ldots , N$, $H_{p+2}$ is a $p+2$-homogeneous Hamiltonian.

From the definition the coefficients in (A.6) satisfy the following:

1. **Symmetry restrictions:** for any $\mathbf {\sigma }= (\sigma _1,\ldots ,\sigma _{p+2})\in \left\{ \pm \right\} ^{p+2}$ and $\mathbf {\jmath }= (j_1,\ldots ,j_{p+2})\in (\mathbb {Z}^*)^{p+2},$ one has
  A.8 $$\begin{aligned} \mathbf{Reality condition:}\text{} \ \ \overline{H_{\mathbf {\jmath }}^{\mathbf {\sigma }}}= H_{\mathbf {\jmath }}^{-\mathbf {\sigma }}, \quad \quad {\textbf {Momentum condition:}} \ \ H_{\mathbf {\jmath }}^{\mathbf {\sigma }} \ne 0 \implies \mathbf {\sigma }\cdot \mathbf {\jmath }=0.\nonumber \\ \end{aligned}$$
2. **Polynomial bound:** There are $\mu , C>0$ and $m\in \mathbb {R}$ such that
  $$\begin{aligned} \left| H_{\mathbf {\jmath }}^{\mathbf {\sigma }}\right| \leqslant C \textrm{max}_3( |j_1|, \ldots , |j_{p+2}|)^\mu \textrm{max}_2( |j_1|, \ldots , |j_{p+2}|)^m. \end{aligned}$$

#### Remark A.9

In view of the momentum condition $\mathbf {\sigma }\cdot \mathbf {\jmath }=0$ in (A.8), one has the equivalence

$$\begin{aligned} \textrm{max}( |j_1|, \ldots , |j_{p+2}|)\sim \textrm{max}_2( |j_1|, \ldots , |j_{p+2}|). \end{aligned}$$

We now define the class of Hamiltonian systems up to homogeneity *N* that we shall use in Section 7.

Let $K, K' \in \mathbb {N}$ with $K'\leqslant K$, $r>0$ and $U \in B_{s_0}^K(I;r)$. Let

A.9 $$\begin{aligned} Z \triangleq \textbf{M}_0(U;t)U \quad \text { with }\quad \textbf{M}_0(U;t) \in \left( \mathcal {M}^0_{K,K',0}[\epsilon _0] \right) ^{2\times 2}\, . \end{aligned}$$

#### Definition A.10

*(Hamiltonian system up to homogeneity* *N**)* Let $N, K, K' \in \mathbb {N}$ with $K \geqslant K'+1$ and assume (A.9). A *U*–dependent system

A.10 $$\begin{aligned} \partial _t Z = {\boldsymbol{J}}_\mathbb {C}\nabla H(Z) + {\boldsymbol{M}}_{> N}(U;t)[U] \end{aligned}$$

is *Hamiltonian up to homogeneity N* if

$\bullet$ *H*(*Z*) is a pluri-homogeneous Hamiltonian as in (A.7);

$\bullet$ ${\boldsymbol{M}}_{> N}(U;t)$ is a matrix of non-homogeneous operators in $\left( \mathcal {M}_{K,K'+1,N+1}[\epsilon _0] \right) ^{2\times 2}$.

We shall perform nonlinear changes of variables which are symplectic up to homogeneity *N* according to the following definition.

#### Definition A.11

*(Symplectic map up to homogeneity* *N**)* Let $p, N \in \mathbb {N}$ with $p \leqslant N$. We say that

A.11 $$\begin{aligned} \mathcal {D}(Z;t) = {\boldsymbol{M}}(Z;t)Z \qquad \text {with} \qquad {\boldsymbol{M}}(Z;t) - \textrm{Id}\in \left( \Sigma \mathcal {M}_{K,K',p}[r,N] \right) ^{2\times 2} \, ,\nonumber \\ \end{aligned}$$

is *symplectic up to homogeneity N*, if its pluri-homogeneous component $\mathcal {D}_{\leqslant N}(Z)\triangleq \big ( \mathcal {P}_{\leqslant N} M(Z;t) \big ) Z$ satisfies

A.12 $$\begin{aligned} \left[ \textrm{d}_Z \mathcal {D}_{\leqslant N}(Z)\right] ^\top \, {\boldsymbol{E}}_\mathbb {C}\, \textrm{d}_Z \mathcal {D}_{\leqslant N}(Z) = {\boldsymbol{E}}_\mathbb {C}+ {\boldsymbol{E}}_{>N}(Z) \quad \text {with} \quad {\boldsymbol{E}}_{>N}(Z) \in \left( \Sigma _{N+1}\widetilde{\mathcal {M}}_q \right) ^{2\times 2} \, .\nonumber \\ \end{aligned}$$

A symplectic map up to homogeneity *N* transforms a Hamiltonian system up to homogeneity *N* into another Hamiltonian system up to homogeneity *N*.

#### Lemma A.12

Let $p, N \in \mathbb {N}$ with $p \leqslant N$, $K, K' \in \mathbb {N}$ with $K \geqslant K'+1$. Let $Z \triangleq \textbf{M}_0(U;t)U$ as in (A.9). Assume $\mathcal {D}(Z;t) = M(Z;t)Z$ is a symplectic map up to homogeneity *N* (Definition A.11) such that

A.13 $$\begin{aligned} M(Z;t) - \text {Id}\in \left\{ \begin{aligned}&\left( \Sigma \mathcal {M}_{K,K',p}[r,N] \right) ^{2\times 2},&\text {if} \quad \textbf{M}_0(U;t) = \textrm{Id}\, , \\&\left( \Sigma \mathcal {M}_{K,0,p}[\breve{r},N] \right) ^{2\times 2} \, ,&\, \forall \breve{r} > 0 \quad \text {otherwise} \, . \end{aligned} \right. \end{aligned}$$

If *Z*(*t*) solves a *U*-dependent Hamiltonian system up to homogeneity *N* (Definition A.10), then the variable $W \triangleq \mathcal {D}(Z;t)$ solves another *U*-dependent Hamiltonian system up to homogeneity *N* (generated by the transformed Hamiltonian).

The following is Lemma 3.19 in [20]:

#### Lemma A.13

Let $p \in \mathbb {N}$, $m \in \mathbb {R}$ and $a (U;x, \xi )$ a real valued homogeneous symbol in $\widetilde{\Gamma }^m_p$. Then the Hamiltonian vector field generated by the Hamiltonian

$$\begin{aligned} H (U)\triangleq \text {Re}\left\langle {\boldsymbol{A}}(U)u \ \bigg \vert \ \bar{u} \right\rangle _{\mathbb {R}}, \qquad {\boldsymbol{A}}(U)\triangleq {{{\,\mathrm{Op^{BW}}\,}}\left( a(U;x, \xi )\right) }, \end{aligned}$$

is

$$\begin{aligned} {\boldsymbol{J}}_\mathbb {C}\nabla H (U) = {{\,\mathrm{Op^{BW}_{vec}}\,}}\left( \textrm{i}a (U;x, \xi )\right) U + {\boldsymbol{R}}(U) U, \end{aligned}$$

where ${\boldsymbol{R}}(U)$ is a real-to-real matrix of homogeneous smoothing operators in $\left( \widetilde{\mathcal {R}}^{-\varrho }_p \right) ^{2\times 2}$ for any $\varrho \geqslant 0$.

The following is Lemma 3.20 in [20]:

#### Lemma A.14

Let $p \in \mathbb {N}$, $m \in \mathbb {R}$ and $\varrho \geqslant 0$. Let

A.14 $$\begin{aligned} X(U) = {\boldsymbol{J}}_\mathbb {C}{{{\,\mathrm{Op^{BW}}\,}}\left( {\boldsymbol{A}}(U;x,\xi )\right) }U + {\boldsymbol{R}}(U)U = {\boldsymbol{J}}_\mathbb {C}\nabla H(U) \end{aligned}$$

be a $(p+1)$-homogeneous Hamiltonian vector field, where

A.15 $$\begin{aligned} {\boldsymbol{A}}(U;x,\xi ) = \begin{pmatrix} a(U;x,\xi ) &  b(U;x,\xi ) \\ \overline{b(U;x,-\xi )}&  \overline{a(U;x,-\xi )} \end{pmatrix} \end{aligned}$$

is a matrix of symbols in  and ${\boldsymbol{R}}(U)$ is a real-to-real matrix of smoothing operators in . Then, we may write that

A.16 $$\begin{aligned} X(U) = {\boldsymbol{J}}_\mathbb {C}{{{\,\mathrm{Op^{BW}}\,}}\left( {\boldsymbol{A}}_1(U;x,\xi ) \right) }U + {\boldsymbol{R}}_1(U)U, \end{aligned}$$

where the matrix of paradifferential operators ${{{\,\mathrm{Op^{BW}}\,}}\left( {\boldsymbol{A}}_1(U;x,\xi )\right) }$ is symmetric, with matrix of symbols

A.17 $$\begin{aligned} {\boldsymbol{A}}_1(U;x,\xi ) = \frac{1}{2} \begin{pmatrix} a + a^\vee &  b + \overline{b} \\ \overline{b}^\vee + b^\vee &  \overline{ a+ a^\vee } \end{pmatrix} \end{aligned}$$

and ${\boldsymbol{R}}_1(U)$ is another real-to-real matrix of smoothing operators in .

The following is Lemma 3.21 in [20]:

#### Lemma A.15

Let $p \in \mathbb {N}$, $m \in \mathbb {R}$ and $\varrho \geqslant 0$. Let ${\boldsymbol{S}}(U)$ be a matrix of spectrally localized homogeneous maps in $\left( \widetilde{\mathcal {S}}_{p} \right) ^{2\times 2}$ which is linearly Hamiltonian of the form

A.18 $$\begin{aligned} {\boldsymbol{S}}(U) = {\boldsymbol{J}}_\mathbb {C}{{{\,\mathrm{Op^{BW}}\,}}\left( {\boldsymbol{A}}(U;x,\xi )\right) } + {\boldsymbol{R}}(U) \, , \end{aligned}$$

where ${\boldsymbol{A}}(U;x,\xi )$ is a real-to-real matrix of symbols in $\left( \widetilde{\Gamma }^m_p \right) ^{2\times 2}$ as in (A.15), and ${\boldsymbol{R}}(U)$ is a real-to-real matrix of smoothing operators in . Then, we may write that

$$\begin{aligned} {\boldsymbol{S}}(U) = {\boldsymbol{J}}_\mathbb {C}{{{\,\mathrm{Op^{BW}}\,}}\left( {\boldsymbol{A}}_1(U;x,\xi )\right) } + {\boldsymbol{R}}_1(U), \end{aligned}$$

where the matrix of symbols ${\boldsymbol{A}}_1(U;x,\xi )$ in  has the form (A.17) and ${\boldsymbol{R}}_1(U)$ is another matrix of real-to-real smoothing operators in . In particular the homogeneous operator ${\boldsymbol{J}}_\mathbb {C}{{{\,\mathrm{Op^{BW}}\,}}\left( {\boldsymbol{A}}_1(U)\right) }$ is linearly Hamiltonian.

### Symplectic Corrections

In this section we provide a symplectic correction to two different class of maps: linearly symplectic spectrally localized maps and smoothing perturbations of the identity. The main result is Theorem 7.1 in [20] that we state below.

#### Theorem A.16

Let $p, N \in \mathbb {N}$ with $p \leqslant N$, $K, K' \in \mathbb {N}$ with $K'+1 \leqslant K$, $r >0$. Let $Z = \textbf{M}_0(U;t)U$ with $\textbf{M}_0(U;t) \in \left( \mathcal {M}^0_{K,K',0}[\epsilon _0]\right) ^{2\times 2}$. Assume that *Z*(*t*) solves a Hamiltonian system up to homogeneity *N*. Consider that

A.19 $$\begin{aligned} \Phi (Z)\triangleq {\boldsymbol{B}}(Z;t)Z, \end{aligned}$$

where

- ${\boldsymbol{B}}(Z;t)-\textrm{Id}$ is a matrix of spectrally localized maps in
  A.20 $$\begin{aligned} {\boldsymbol{B}}(Z;t) - \text {Id}\in {\left\{ \begin{array}{ll} \left( \Sigma \mathcal {S}_{K,K',p}[r,N]\right) ^{2\times 2}, \qquad \qquad \text {if} \quad \textbf{M}_0(U;t) = \textrm{Id}\, , \\ \left( \Sigma \mathcal {S}_{K,0,p}[\breve{r},N]\right) ^{2\times 2} \, , \, \forall \breve{r} > 0 \quad \text {otherwise} \, . \end{array}\right. } \end{aligned}$$
- ${\boldsymbol{B}}(Z;t)$ is linearly symplectic up to homogeneity *N*, according to Definition A.4.

Then, there exists a real-to-real matrix of pluri–homogeneous smoothing operators ${\boldsymbol{R}}_{\leqslant N}( \cdot )$ in $\left( \Sigma _p^N \widetilde{\mathcal {R}}^{-\varrho }_q\right) ^{2\times 2}$, for any $\varrho >0$, such that the non-linear map

$$\begin{aligned} Z_+ \triangleq \big ( \textrm{Id}+ {\boldsymbol{R}}_{\leqslant N}(\Phi (Z) )\big ) \Phi (Z) \end{aligned}$$

is symplectic up to homogeneity *N* and thus $$Z_+ $$ solves a system which is Hamiltonian up to homogeneity *N*.

Given a map of the form $U \mapsto U+ {\boldsymbol{J}}_\mathbb {C}\nabla H_{p+2}(U)$ where ${\boldsymbol{J}}_\mathbb {C}\nabla H_{p+2}(U)$ is a Hamiltonian, smoothing vector field, we find a correction which is symplectic up to homogeneity *N*.

#### Lemma A.17

Let $p, N \in \mathbb {N}$ with $p \leqslant N$. Let $Y_{p+1}(U)= {\boldsymbol{J}}_\mathbb {C}\nabla H_{p+2}(U)$ be a homogeneous Hamiltonian smoothing vector field in $\widetilde{{\mathfrak X }}_{p+1}^{-\varrho }$ for some $\varrho \geqslant 0$. Then there is a map of the form

A.21 $$\begin{aligned} \mathfrak {F}_{\leqslant N}(U)= U + Y_{p+1}(U) + F_{\geqslant (p+2)}(U), \qquad F_{\geqslant (p+2)}(U)\in \Sigma _{p+2}^N \widetilde{\mathfrak X}^{-\varrho }_q ,\nonumber \\\end{aligned}$$

which is symplectic up to homogeneity *N* (Definition A.11).

#### Proof

It is a direct consequence of Lemmata 2.27 and 3.14 in [62]. A careful examination of the proofs reveals that $F_{\geqslant (p+2)}(U)$ actually belongs to $\Sigma _{2p+1}^N \widetilde{\mathfrak X}^{-\varrho }_q$. However, since this stronger result is not required for our purposes, we prefer to state the weaker conclusion $F_{\geqslant (p+2)}(U) \in \Sigma _{p+2}^N \widetilde{\mathfrak X}^{-\varrho }_q$. $\square$

#### Remark A.18

The map $\mathfrak {F}_{\leqslant N}(U)$ in (A.21) is indeed the truncation up to homogeneity *N* of the time-one flow generated by the Hamiltonian vector field ${\boldsymbol{J}}_\mathbb {C}\nabla H_{p+2}(U)$.

## Auxiliary Flows and Conjugations

The following conjugation Lemmata A.19 and A.20 are used in the nonlinear Hamiltonian Birkhoff normal form reduction performed in Section 7. Their proof can be found in Appendix A of [20].

The following hypothesis shall be assumed in both Lemmata A.19 and A.20:

**Assumption (A)**:   Assume $Z \triangleq \textbf{M}_0(U;t)U$ where $\textbf{M}_0(U;t) \in \left( \mathcal {M}^0_{K,K',0}[\epsilon _0] \right) ^{2\times 2}$, $U \in B^K_{s_0, \mathbb {R}}(I;\epsilon _0)$ for some $\epsilon _0,s_0>0$ and $0\leqslant K' \leqslant K$. Let $N \in \mathbb {N}$ and assume that *Z* solves the system

B.1 $$\begin{aligned} \partial _t Z = {{\,\mathrm{Op^{BW}_{vec}}\,}}\left( \textrm{i}\omega _{\gamma ,{\texttt{b}}}(\xi )+\textrm{i}a_{\leqslant N}(Z;\xi )+ \textrm{i}a_{>N}(U;t,\xi )\right) Z + \boldsymbol{R}_{\leqslant N}(Z)Z+ \boldsymbol{R}_{>N}(U;t)U , \end{aligned}$$

where

- $a_{\leqslant N} (Z;\xi )$ is a real valued pluri-homogeneous symbol, independent of *x*, in $\Sigma _2^N \widetilde{\Gamma }^{\frac{3}{2}}_q$;
- $a_{>N} (U;t,\xi )$ is a non-homogeneous symbol, independent of *x*, in $\Gamma ^{\frac{3}{2}}_{K,K',N+1}[\epsilon _0]$ with imaginary part $\textrm{Im} \, a_{>N} (U;t,\xi )$ in $\Gamma ^{0}_{K,K',N+1}[\epsilon _0]$;
- $\boldsymbol{R}_{\leqslant N} (Z)$ is a real-to-real matrix of pluri-homogeneous smoothing operators in $\left( \Sigma _1^N \tilde{\mathcal {R}}^{-\varrho }_q \right) ^{2\times 2}$;
- $\boldsymbol{R}_{>N} (U;t)$ is a real-to-real matrix of non-homogeneous smoothing operators in $\left( \mathcal {R}^{-\varrho }_{K,K',N+1}[\epsilon _0] \right) ^{2\times 2}$.

### Lemma A.19

(Conjugation under the flow of a Fourier multiplier) Assume **(A)** at page 66. Let $g_p(Z;\xi )$ be a *p*–homogeneous real symbol independent of *x* in $\widetilde{\Gamma }^{\frac{3}{2}}_p$, $p \geqslant 2$, that we expand as

B.2 $$\begin{aligned} g_p(Z;\xi ) = \sum _{ (\vec {\jmath }_p, \vec {\sigma }_p) \in {\mathfrak {T}}_p } G_{\vec {\jmath }_p}^{\vec {\sigma }_p}(\xi ) z_{\vec {\jmath }_p}^{\vec {\sigma }_p} \, , \qquad \overline{G_{\vec {\jmath }_p}^{-\vec {\sigma }_p}}(\xi )= G_{\vec {\jmath }_p}^{\vec {\sigma }_p}(\xi )\in \mathbb {C}, \end{aligned}$$

and denote by $\pmb {\mathcal {G}}_{g_p}(Z)\triangleq \pmb {\mathcal {G}}_{g_p}^1(Z)$ the time 1-flow defined in (A.5) generated by ${{\,\mathrm{Op^{BW}_{vec}}\,}}\left( \textrm{i}g_p(Z;\xi )\right)$. If *Z*(*t*) solves system (B.1), then the variable

B.3

solves the system

B.4 $$\begin{aligned} \partial _t W= &   \textrm{i}\boldsymbol{\omega }_{\gamma ,{\texttt{b}}}(D) W +{{\,\mathrm{Op^{BW}_{vec}}\,}}\left( \textrm{i}a_{\leqslant N}^+(W;\xi )+ \textrm{i}a_{>N}^+(U;t,\xi )\right) W \nonumber \\  &   + \boldsymbol{R}_{\leqslant N}^+(W)W+ \boldsymbol{R}^+_{>N}(U;t)U , \end{aligned}$$

where

- $a^+_{\leqslant N} (W;\xi )$ is a real valued pluri-homogeneous symbol, independent of *x*, in $\Sigma _2^N \widetilde{\Gamma }^{\frac{3}{2}}_q$, with components
  B.5 $$\begin{aligned} \begin{aligned}&\mathcal {P}_{\leqslant p-1} [a^+_{\leqslant N}(W;\xi )] = \mathcal {P}_{\leqslant p-1}[a_{\leqslant N}(W;\xi )] \, , \\&\mathcal {P}_{ p} \left[ a^+_{\leqslant N}(W;\xi )\right] = \mathcal {P}_{ p} \left[ a_{\leqslant N}(W;\xi ) \right] + g^+_p(W;\xi ), \end{aligned} \end{aligned}$$
  where $g^+_p(W;\xi ) \in \widetilde{\Gamma }^{\frac{3}{2}}_p$ is the real, *x*-independent symbol
  B.6 $$\begin{aligned} g^+_p(W;\xi )\triangleq \sum _{(\vec {\jmath }_p, \vec {\sigma }_p) \in {\mathfrak {T}}_p}\textrm{i}\big (\vec {\sigma }_{p}\cdot \vec {\omega }_{\gamma ,{\texttt{b}}}(\vec {\jmath }_p) \big ) G_{\vec {\jmath }_p}^{\vec {\sigma }_p}(\xi ) w_{\vec {\jmath }_p}^{\vec {\sigma }_p} ; \end{aligned}$$
- $a^+_{>N} (U;t,\xi )$ is a non-homogeneous symbol, independent of *x*, in $\Gamma ^{\frac{3}{2}}_{K,K',N+1}[\epsilon _0]$ with imaginary part $\textrm{Im} \, a_{>N}^+ (U;t,\xi )$ belonging to $\Gamma ^{0}_{K,K',N+1}[\epsilon _0]$;
- $\boldsymbol{R}^+_{\leqslant N}(W)$ is a real-to-real matrix of pluri–homogeneous smoothing operators in $\left( \Sigma _1^N \tilde{\mathcal {R}}^{-\varrho \! +\! c(N,p)}_q \right) ^{2\!\times \! 2}$ for some $c(N,p) >0$ (depending only on *N*, *p*) and fulfilling
  B.7 $$\begin{aligned} \mathcal {P}_{\leqslant p} [\boldsymbol{R}^+_{\leqslant N}(W)] = \mathcal {P}_{\leqslant p}[\boldsymbol{R}_{\leqslant N}(W)] \, ; \end{aligned}$$
- $\boldsymbol{R}^+_{>N}(U;t)$ is a real-to-real matrix of non-homogeneous smoothing operators in $\left( \mathcal {R}^{-\varrho +c(N,p)}_{K,K',N+1}[\epsilon _0] \right) ^{2\times 2}$.

The next lemma describes how a system is conjugated under a smoothing perturbation of the identity.

### Lemma A.20

(Conjugation under a smoothing perturbation of the identity) Assume **(A)** at page 66. Let $F_{\leqslant N}(Z)$ be a real-to-real matrix of pluri-homogeneous smoothing operators in $\left( \Sigma _p^N \widetilde{\mathcal {R}}_q^{-\varrho '} \right) ^{2\times 2}$ for some $\varrho ' \geqslant 0$. If *Z*(*t*) solves (B.1) then the variable

B.8 $$\begin{aligned} W\triangleq \mathcal {F}_{\leqslant N}(Z)\triangleq Z + F_{\leqslant N}(Z)Z \end{aligned}$$

solves

B.9 $$\begin{aligned} \partial _t W&= \textrm{i}\boldsymbol{\omega }_{\gamma ,{\texttt{b}}}(D) W +{{\,\mathrm{Op^{BW}_{vec}}\,}}\left( \textrm{i}a^+_{\leqslant N}(W;\xi )+ \textrm{i}a^+_{>N}(U;t,\xi )\right) W \nonumber \\&\quad + \boldsymbol{R}^+_{\leqslant N}(W)W+ \boldsymbol{R}^+_{>N}(U;t)U, \end{aligned}$$

where

- $a^+_{\leqslant N} (W;\xi )$ is a real valued pluri-homogeneous symbol, independent of *x*, in $\Sigma _2^N \widetilde{\Gamma }^{\frac{3}{2}}_q$, with components
  B.10 $$\begin{aligned} \mathcal {P}_{\leqslant p+1} [a^+_{\leqslant N}(W;\xi )] = \mathcal {P}_{\leqslant p+1}[a_{\leqslant N}(W;\xi )] \, ; \end{aligned}$$
- $a^+_{>N} (U;t,\xi )$ is a non-homogeneous symbol, independent of *x*, in $\Gamma ^{\frac{3}{2}}_{K,K',N+1}[\epsilon _0]$ with imaginary part $\textrm{Im} \, a_{>N}^+ (U;t,\xi )$ belonging to $\Gamma ^{0}_{K,K',N+1}[\epsilon _0]$;
- $\boldsymbol{R}^+_{\leqslant N}(W)$ is a real-to-real matrix of pluri–homogeneous smoothing operators in $\left( \Sigma _1^N \tilde{\mathcal {R}}^{-\varrho _*}_q \right) ^{2\times 2}$, $\varrho _* \triangleq \min {(\varrho , \varrho '-\frac{3}{2})}$ ($\varrho \ge 0$ is the smoothing order in Assumption **(A)** at page 66), with components
  B.11 $$\begin{aligned} \mathcal {P}_{\leqslant p-1} [\boldsymbol{R}^+_{\leqslant N}(W)] = \mathcal {P}_{\leqslant p-1}[\boldsymbol{R}_{\leqslant N}(W)] \ , \end{aligned}$$
  and, denoting $\boldsymbol{F}_p(W) \triangleq \mathcal {P}_p(\boldsymbol{F}_{\leqslant N}(W))$ in $\left( \widetilde{\mathcal {R}}^{-\varrho '}_p \right) ^{2\times 2}$, one has
  B.12 $$\begin{aligned} \mathcal {P}_{ p} [\boldsymbol{R}^+_{\leqslant N}(W)] = \mathcal {P}_p[\boldsymbol{R}_{\leqslant N}(W)]&+d_W \big (\boldsymbol{F}_{p}(W)W\big ) \left[ i\boldsymbol{\omega }_{\gamma ,{\texttt{b}}}(D)\right] -i\boldsymbol{\omega }_{\gamma ,{\texttt{b}}}(D)\boldsymbol{F}_{p}(W); \end{aligned}$$
- $\boldsymbol{R}^+_{>N}(U;t)$ is a real-to-real matrix of non-homogeneous smoothing operators in $\left( \mathcal {R}^{-\varrho _*}_{K,K',N+1}[\epsilon _0] \right) ^{2\times 2}$.

In addition, if $\mathcal {F}_{\leqslant N}(Z)$ in (B.8) is the symplectic up to homogeneity *N* map associated to a Hamiltonian vector field $\boldsymbol{G}_p(Z)Z= \boldsymbol{J}_\mathbb {C}\nabla H_{p+2}(Z)$ as per Lemma A.17, where $\left( \boldsymbol{G}_p(Z)\in \widetilde{\mathcal {R}}^{-\varrho '}_p \right) ^{2\times 2}$ has Fourier expansion

B.13 $$\begin{aligned} \big (\boldsymbol{G}_p(Z)Z\big )^\sigma _k= \!\!\!\!\!\!\!\!\!\!\!\!\!\!\!\!\!\! \sum _{ (\mathbf {\jmath }_{p+1}, k, \vec {\sigma }_{p+1}, - \sigma ) \in {\mathfrak {T}}_{p+2} } \!\!\!\!\!\!\!\!\!\!\!\!\!\!\!\!\!\! G_{\mathbf {\jmath }_{p+1},k}^{\vec {\sigma }_{p+1},\sigma } z_{\vec {\jmath }_{p+1}}^{\vec {\sigma }_{p+1}} \, , \end{aligned}$$

then (B.12) reduces to

B.14 $$\begin{aligned} \mathcal {P}_{ p} [\boldsymbol{R}^+_{\leqslant N}(W)] = \mathcal {P}_{p}[\boldsymbol{R}_{\leqslant N}(W)] + \boldsymbol{G}_p^+(W), \end{aligned}$$

where $\boldsymbol{G}_p^+(W)\in \left( \widetilde{\mathcal {R}}^{-\varrho '+\frac{3}{2}}_p \right) ^{2\times 2}$ is the smoothing operator with Fourier expansion

B.15 $$\begin{aligned} \begin{aligned}&(\boldsymbol{G}_p^+(W)W)^\sigma _k= \!\!\!\!\!\!\!\!\!\!\!\!\!\!\!\!\!\! \sum _{ (\mathbf {\jmath }_{p+1}, k, \vec {\sigma }_{p+1}, - \sigma ) \in {\mathfrak {T}}_{p+2} } \!\!\!\!\!\!\!\!\!\!\!\!\!\!\!\!\!\! \textrm{i}\big ( \vec {\sigma }_{p+1}\cdot \vec {\omega }_{\gamma ,{\texttt{b}}}(\vec {\jmath }_{p+1})- \sigma {\omega }_{\gamma ,{\texttt{b}}}(k)\big )G_{ \vec {\jmath }_{p+1},k}^{\vec {\sigma }_{p+1},\sigma } \, w_{\vec {\jmath }_{p+1}}^{\vec {\sigma }_{p+1}} \,. \end{aligned}\nonumber \\ \end{aligned}$$
